# Abstracts Accepted by Title

**DOI:** 10.1155/1990/34262

**Published:** 1990


### BT001 THE DEFINITIVE BILIARY SURGERY IN THE EARLY OPERATION FOR ACUTE FULMINANT PANCREATITIS

A.Agorogiannis and I.Agorogianni

Surgical Department,General Hospital of Larisa, Larisa-Greece

During the last 12 years (Jan.1978 to Dec 1989) at the surgical department of our Institution 2220 patients were operated for gallstone desease of the biliary system.Among then 224 patients (10.09%) were operated and had gallstone acute pancreatitis (AP).Cholelithiasis and AP were documented in all patients by operation,clinical, x-rays and biochemical findings.Of the 224 patients in 47 the operation was done early and without improvement of their AP.This was done into the first week of their treatment (group A patients).In this group of patients the operation was done as a urgent procedure and it was also for the definitive treatment of their gallstone desease (i.e. cholecystectomy,choledochotomy usually,necrosectomy-debridement of the necrotic pancreatic and peripancreatic tissue in some patients and multiple external drainage to all of the patients. In many of them the diagnosis for the first time was put during laparatomy.The laparatomy was done with the misdiagnosis of acute cholecystitis,perforated peptic ulcer or mesenteric infarction.In this group A of patients we had 9 deaths and the mortality rate was 19%. Onc hundred and senenty seven patients were operated for their biliary desease ellectively,after the crisis of AP had subsided with the conservative treatment.Usually this group of patients (group B), was operated at the sameadmission to the Hospital and around the 15th day from the onset of the symptoms.In these group of patients we had no deaths (mortality 0%).

The timing of biliary surgery remains controversial in patients with acute panreatitis associated with cholelithiasis.We usually operate on these patients after the 15th day since the onset of their symptoms and always at the same admission to the Hospital.When there is no improvement with the conservative treatment of acute pancreatitis and when the diagnosis is put for the first time during laparatomy, we conclude in this parer,that the definitive surgery for the lithiasis of the biliary tract must be done at the same time.This technicaly has not difficulties for the experienced surgeon,does not adds to the complications and mortality,saves the patient by a second operation and protects him of recurrences of AP during the waitting time.

### BT002 GALLBLADDER CANCER : THE PLACE OF PALLIATIVE SURGERY.

M C ALDRIDGE, D CASTAING, H BISMUTH
Hepatobiliary Surgery and Liver Transplant Research Unit
South Paris Faculty of Medicine, Hopital Paul Brousse,
94800 Villejuif, FRANCE.

Between 30% and 60% of patients with gallbladder cancer present with obstructive jaundice. Indeed, as many as 25% of patients with 'hilar cholangiocarcinoma' in fact have gallbladder cancers which have spread to involve the hilar region. The relief of jaundice may be either surgical (bypass or intubation) or endoscopic (prosthesis). Endoscopic treatment is popular but the quality of survival may be poor as a 30-day mortality of over 20%, early cholangitis and tube blockage are not uncommon. We present our experience with surgical palliation.
Between 1964-1988, 55 jaundiced patients with advanced gallbladder cancer presented as 'hilar cancer'. They comprised 36 women and 19 men of median age 60 yr (range 30-86 yr). All underwent surgical palliation by biliary-enteric bypass by intrahepatic anastomosis (segment III duct), hepatoanastomosis (Longmire) or surgical intubation (T-tube or U-tube) (TABLE). Gastrojejunostomy was performed for duodenal involvement in 22 patients (40%). Quality of survival was assessed by 'COMFORT INDEX' (duration of well-being ÷ duration of survival x 100%), ideal palliation being 100%.

| SURGERY | n | 30-day mortality | SURVIVAL 6 months | SURVIVAL 12 months | COMFORT INDEX 100% 6 months | COMFORT INDEX 100% 12 months |
|---|---|---|---|---|---|---|
| INTRAHEPATIC ANASTOMOSIS | 29 | 2 (6.9%) | 52% | 22% | 67% | 46% |
| HEPATOANASTOMOSIS or SURGICAL INTUBATION | 26 | 5 (19.2%) | 39% | 6.5% | 19% | 0% |

Relief of obstructivE jaundice using an intrahepatic anastomosis (segment III duct) provides good palliation with a low operative mortality. The high operative mortality and poor survival of patients undergoing surgical intubation reflect the more advanced nature of their disease. Duodenal involvement is common and is easily bypassed as part of surgical palliation.

### BT003 INDICATORS OF PROGNOSIS IN PRIMARY LIVER CANCER

A. Altendorf-Hofmann, R. Stangl, J. Scheele
Department of Surgery, University Hospital, Erlangen, FRG

Primary liver cancer (PLC) in Western populations comprises a variety of malignancies with an inconsistent biologic behaviour. In contrast to the Japanese experience, it is less frequently associated with viral hepatitis, and consequent cirrhosis. Moreover, both cancer and cirrhosis may differ geographically (Adson 1988).

The records of 163 patients treated for PLC from 1970 through 1988 were reviewed retrospectively. 63 patients (39 %) underwent hepatic resection, which was classified "potentially curative" in 43 cases (26 %). Liver cirrhosis was present in 30 % of patients undergoing resection, and in 40 % of those who did not. The overall incidence was 38 %, being 51 % in 107 hepatocellular cancer patients, and 15 % in 65 other malignancies.

Treatment included common hepatectomies in 36 patients, segment-orientated resections in 11 patients, and non-anatomical procedures in 16 cases. Significant extrahepatic surgery was simultaneously performed in 14 patients ( hilar resection 3, portal vein resection 1, vena cava replacement of 1, total gastrectomy 2, partial gastrectomy 2, various minor procedures 7). There was an unacceptable high mortality of 19 % (12 cases), mainly associated with cirrhosis (n=8), and emergency procedures (n=1).

Excluding 30 day mortality, median survival time was 53 months for curative interventions, 7 months following palliative procedures, and 4 months in surgically untreated patients. Corresponding five-year survival figures were 47 %, 16 %, and 4 %, respectively. Of the 100 patients who did not undergo surgical treatment, one pediatric patient is in complete remission ten years after systemic chemotherapy of an undifferentiated carcinoma, whereas non of the others survived four years yet.

Within the curative group, there was no significant difference between solitary and multiple lesions (49% vs. 36%), or between HCC and other malignancies (45% vs.49%). In contrast, liver cirrhosis was associated with early multifocal recurrence in eight of nine patients, and resulted in a maximum survival time of 63 months. Considering this poor outcome, the significant operative risk, and the potential benefit of transplantation in highly selected cases, we became uncertain about the value of hepatic resection in this particular group of patients.

References:
Adson MA. In Blumgart LH, Churchill Livingstone, 1988: 1153-1166
Ringe B, Wittekind C, Bechstein WO, et al: Ann Surg 1989; 209: 88-98

**BT004** EXPERIENCE WITH 160 PANCREATIC AND AMPULLARY CARCINOMAS

I. Baca, I. Klempa, J. Menzel

Allgemein-Chirurgische Klinik, ZKH

St.-Jürgen-Straße, 2800 Bremen,FRG

Long-time survival rates in cases of pancreatic cancer are still different in international reports. An analysis of surgical procedures, complications and long-time survival of those patients, who underwent operation with palliative or curative intent in our department was therefore done from 1983 to 1989. The group consisted of 81 men (63±11 years) and 79 women (65±11 years). 132 patients suffered from pancreatic duct carcinoma and 28 from ampullary carcinoma. Operative procedures for curative intent were done as follows:

| Location | Resectability rate n(%) | Part. pancreatect. | Dist.res. | Local exc. |
|---|---|---|---|---|
| Ampulla | 20 (71) | 16 | - | 4 |
| Pancreas | 50 (37) | 37 | 12 | 1 |
| Total | 70 (43) | 53 | 12 | 5 |

Postoperative mortality rate was 5,7 % in curative and 10 % in palliative operations. Overall morbidity was 35%, with no significant difference between curative and palliative procedures. 14 patients (9%) had to undergo reintervention. Five-years-survival of 66 patients with radical surgery was 18 % in cases with ductal carcinoma and 25% with ampullary carcinoma (Kaplan-Meier-method). On the contrary, no patient with palliative procedure survived more than 3 years.

In our experience morbidity is still high, whereas mortality has decreased in the last years in spite of a more aggressive approach. Survival rates are far from being satisfactory, but extensive surgery provides some hope.

**BT005** # THE BILIARY-ENTERIC ANASTOMOSIS

**C.Battersby - Royal Brisbane Hospital**
**Australia.**

Indications for biliary-enteric anastomosis include malignant and benign stricture, multiple stones in the bile duct, retained or recurrent bile duct stone, and chronic inflammatory disease (e.g. sclerosing cholangitis and chronic pancreatitis).

A variety of biliary structures (e.g. left hepatic duct, bile duct, gall bladder) has been anastomosed to parts of the G.I. tract including stomach, duodenum and jejunum. "Although there is general consensus concerning the need for these procedures in selected patients, there is no consensus concerning which technique is preferable, or under what circumstances internal drainage should be used." (1) The appearance of endoscopic stenting procedures and endoscopic sphincterotomy has provided other alternatives.

Choice of procedure will depend upon the site and type of pathology present, and the age of and fitness of the patient, as well as the size of the biliary system (e.g. Roux-en-Y hepatic jejunostomy is the procedure of choice for benign high biliary stricture in a fit patient).

Recommendations are :

(1) The bile duct is superior to the gall bladder for anastomosis.

(2) Side-to-side anastomosis is technically easier and less catastrophic if there is a leak.

(3) The length of the Roux loop of jejunum should be at least 50cm.

(4) The loop is best brought up retrocolic or retrogastric.

(5) Transhepatic stenting is not usually necessary.

(6) Antibiotic prophylaxis is wise.

References:

1. Jordan, G.L.Jnr. **Current Problems in Surgery** 1982 Vol.19:No.12:p.758

BT006

### MAJOR HEPATIC SURGERY FOR BENIGN LIVER DISEASE

G.Belli,G.Romano,A.Monaco,M.F.Armellino,A.D'Agostino; University of Naples,II^ Faculty of Medicine and Surgery; General Surgery and Organ Transplantations; Italy

Benign lesions of the liver are rare but are found more often today than heretofore because of improvements in diagnostic imaging. The successful results of hepatic resections for malignant liver tumours parallel increasing acceptance of major hepatic surgery as appropriate management of benign liver diseases(1-2).The indications for major hepatic surgery in 18 patients operated on from 1985 to 1989 and results are presented. There were 6 male and 12 female;the average age was 53.3 years (Range:42-67). Hepatic resections for trauma are excluded.

Eight hydatid cysts, five hemangioma and five benign tumours were removed. Four right hepatectomy; one extended right hepatectomy ; two left hepatectomy; four bisegmentectomy (segments II-III : two ; segments IV-V : two); two wedge resections and five cysto-pericystectomy without open the cyst were performed.

Cystopericystectomy has been considered mayor hepatic surgery because it poses equivalent operative problems (Pringle manoeuvre,digitoclasia, intraoperative hemorrage, etc.).

Overall morbidity rate was 22.2% with no postoperative mortality. The most frequent complication was persistent pleural effusion which required thoracentesis in two patients; none required reoperation.

Major hepatic surgery is an effective treatment for benign lesions and can be accomplished with acceptable morbidity and mortality provided that carefull selection of the patients and standardized operative technique are used.

1) M.A.Adson "Primary hepatocellular cancers-Western experience" in: L.H.Blumgart 1988 (eds),Surgery of the liver and biliary tract ; Churchill Livingstone, Edinburgh: 1153-1165

2) J.H.Foster "Benign liver tumours" in: L.H.Blumgart 1988 (eds), Surgery of the liver...;Churchill Livingstone,Edinburgh:1115-1127

### BT007 STENTING OF BILIARY TRACT FOR BILE-DUCT STRICTURE

A. Bilge
Erciyes University,Medical School,Kayseri,Turkey

Bypass of bile ducts for malignant or benign (post-cholecystectomy stricture and secondary cholangitis) stricture is facilitated by stenting biliary tree. Between 1984-1989 we have undertaken 16 such operations on 16 patients (8 M, 8F) with a median age of 46.8 years (range 25-71). There were 6 malignant and 10 benign strictures of the bile duct.

In 6 malignant strictures; 4 transtumoral stenting, 2 cholangiojejunostomy over stent were conducted on patients with obstructive jaundice. In 5 patients with post-cholecystectomy stricture; 2 reconstruction of common hepatic duct stenosis, one reconstruction of biliodigestive stoma (hepaticojejunostomy), one reconstruction of hepaticojejunostomy stoma plus cholangiojejunostomy were carried out over stent. In three patients with secondary cholangitis due to intrabiliary rupture of hydatid cyst, transhepatic stent was used for daily washing out the biliary tree. In one patient, stricture of common hepatic ducts due to injection of formalin into hydatid cyst cavity was repaired with flap of cystic duct over stent. In one patient with intrahepatic stone, bilateral transhepatic tube splint was put to prevent obstruction of bile ducts with retained stones.

Transhepatic stents were hold continuously up to the end of the life in patients with malignant stricture.The others were removed at a median of 6 months (range1-12 months). Clinical and laboratory findings showed that,sufficient and efective bile flow was obtained in all patients.There were three postoperative deaths. One patient developed hemorhage due to stenting.

In conclusion surgical transhepatic tube splint is convenient to solve obstructive jaundice in malignant stricture. It provides a chance for chemotherapy. Tube splint also prevents early bile leakage and stenosis of reconstructive procedures for benign stricture, and provides to wash out biliary tree in patients with cholangitis.

BT008 EXPERIMENTAL PANCREATIC ASCITES

E Botta, P Vigano, F Esposito, P Premoli

Via S Carlo No 17, Lurate Caccivio

Como, Italy

Pancreatic ascites is a rather ill defined clinical entity, the pathogenesis of which is poorly assessed. Experimental reports dealing mainly with open duct pancreatic transplant failed to demonstrate tyrue pancreatic ascites in several animal species.

We decided to prepare some new experimental modes to ascertain:

1 The feasibility of producing pancreatic ascites in animals without any other cause of peritoneal effusion (lymphatic or hepatic damage)
2 The possible relevance of enzymatic activation in the disease.

As in our first experiment with rats we did not obtain a clear-cut answer we decided to employ pigs, because of the opening of the Wirsung duct into the duodenum completely independent from the bile duct opening. Two groups of animals were employed. In the first the pancreatic duct was severed a few mm from the duodenum, the distal intraduodenal segment ligated and the proximal one left open in the peritoneal cavity (unactivated juice). In the second group a segment of few cm of duodenum distal to the bile duct opening was isolated, leaving the pancreatic duct opening in it untouched, and left free to pour pancreatic secretion, activated from contact with duodenal mucosa, in the peritoneum. The continuity of the duodenum was then re-established.

All the animals with unactivated pancreatic juice pouring into the peritoneal cavity showed acute necrotic pancreatitis, diffuse or focal, but not peritoneal effusion; while the animals of the second group (activated juice) showed peritoneal effusion (400 to 1900 ml) with very high amylase activity.

### BT009 EXTRAHEPATIC BILIARY TRACT INJURIES BY BLUNT TRAUMA

D Bouras, S Samoilis, G Papadakis
General Hospital KAT, Athens, Greece

Trauma to the extrahepatic biliary tract is rare but, if over looked or improperly managed, may be assosiated with significant morbidity and mortality.
Among 1100 patients(1970-1988) undergoing laparotomy for acute blunt trauma, there were 5 (0.45%) injuries to the extrahepatic biliary tract. 4 of them were due to Road Traffic Accident. In 2 cases common bile duct was injuried, in 2 cases right and in 1 case right and common hepatic duct. The indications for operation was shock due to an associated injury. Associated intra-abdominal trauma was always present. Common bile duct ruptures were treated by cholopeptic anastomoses while the ruptures in bile ducts was treated by using stiches and common bile duct drainage by a T-tube. Rostoperative complications were two biliary leak. In one case the right hepatic duct injury was overlooked at the time of initial abdominal exploration. The mortality was due to associated injuries, 2 out of 5 patients died, their postoperative course were characterized by multiorgan failure. We conclude that in extrahepatic bile duct trauma, early recognition of the injury is essential if serious morbidity and mortality are to be avoided.

### BT010 PECULIARITIES OF LASER PANCREATIC RESECTION

E. I. Brekhov, A. N. Severtsev,
I. Y. Kuleshov. Surgical Clinic,
Hospital No. 51, Moscow, USSR

Though at present a great number of studies are dedicated to pancreatic surgery, there are still a lot of problems concerning the surgery of this organ. The use of laser scalpel (CO2-laser) in pancreatic transection is very promising due to the fact that the laser beam can seal blood vessels and pancreatic ducts and sterilise the surface of the resected organ. A number of experimental and clinical studies show the advantage of laser scalpel over the conventional instruments (scalpel, electrocautery knife). Unfortunately, according to bibliography, only 28 pancreatic resections have been performed in 5 surgical institutes in the USSR which give no opportunity to assess the peculiarities of this organ surgery. The aim of our study is to establish the influence of such factors as: complete hemostasis only by means of laser beam and in combination with electrocoagulation and vessel litigation, unsutured pancreatic stump after its transection with CO2 laser beam, pancreatic stump plastics, suturing of the main pancreatic duct.

The work is performed in the Surgical Clinic and based on the results of the observation of 36 patients with'laser' distal pancreatic resection. All the patients were subdivided into 6 groups and the results were compared in the following way: 1. Complete hemostasis due to the laser irradiation and 2. hemostasis due to the combination of laser irradiation, electrocoagulation and suture material; 3. Suture of pancreatic stump and 4. giving up the suture; 5. Pancreatic stump plastics and 6. giving up the pancreatic stump plastics. In neither case the significant difference was demonstrated ($P>0.05$). The comparison of the results of the groups with the ligature of the main pancreatic duct and without it showed that the latter group gave better results ($P<0.001$). On the basis of the obtained data, one can see that the use of laser scalpel unifies and simplifies many technical questions of pancreatic surgery.

BT011

### LOW-ENERGY LASER IRRADIATION IN COMPLEX TREATMENT OF MECHANICAL JAUNDICE

B.S.Briskin, A.K.Polonsky, I.M.Aliev, B.A.Razygrin

The Semashko Moscow Medical Institute

80 patients with mechanical jaundice due to choledocholithiasis were irradiated with low-energy laser over the near infrared range of spectrum. The Soviet AMLT-01 device was used. The output power of irradiation is 15 mvt, power density - 5m Vt/cm$^2$. The irradiation was given before decompression of biliary tracts during 3-5 days and after decompression the patients had got 7-10 courses of irradiation with 5 minutes exposition. The duration of jaundice was more 10 days, blood bilirubinemia exceeded 10 mkmol/l. The effectiveness of treatment evaluated according to clinical picture of the disease, dynamic of biochemical values, rheohepatography.

Laser therapy before biliary tracts decompression gave positive effects resulting in improving of metabolism and liver microcirculation, which normalized hepatocyte function suggesting beneficial conditions for biliary tract surgery. After decompression Laser therapy increased the effect of treatment, contributing to disappearance of jaundice, improving liver function and decreasing complication rate and lethality.

### BTO12 MANAGEMENT OF SPLENIC TRAUMA

CJ Cahill Westminster Hospital, JA Pain King's College Hospital, London UK.

A postal questionnaire was sent to a random 25% sample of all UK General surgeons (249 surgeons) in 1988. 236 (95%) replied, and 203 of these performed upper abdominal surgery.

61% of these attempt to salvage a damaged spleen, and a further 5% sometimes do so. The remaining 32% do not attempt splenic repair.

Following splenectomy, 28% reimplant splenic tissue, and 59% give Pneumococcal vaccine. Only 28% give prophylactic antibiotics to adults, and 59% give them to children. 74% of surgeons did not specify the duration of antibiotic usage, and the remainder stated periods from one month to 10 years, or up to a specified age for children.

These figures suggest increasing awareness of the importance of the spleen from attempts at salvage, but persisting uncertainty about management of the patient after splenectomy.

### BT013 HEMORRHAGE IN CHRONIC PANCREATITIS: EXPERIENCE IN SIX CASES

F. Callejas Neto; J.C. Pareja; E.A. Chaim; L.S. Leonardi; I.F.S.F. Boin

Unicamp Medical School, Campinas-SP,Brazil

Six patients with hemorrhage associated to chronic pancreatitis were studied during the period 1980 to 1989. All the patients the pancreatitis was associated with alcoholism.

Three patients had hematemesis and melena; of these two showed bleeding from the wirsung duct, observing blood comming from the papilla of Vater during duodenoscopy, and the other from a pseudoaneurism rupturing into the stomach an abdominal cavity. In the other three patients, the hemorrhage occurred in the pancreatitic cysts.

The Blood vessels where the bleeding occurred were the splenic artery in four patients and the gastroduodenal artery in two patients.

The pre-operative diagnosis was estabilished in five cases by endoscopy, selective arteriography, ultrassonography, CT or ERCP. In one patient the diagnosis was made in the course of the operation.

Three patients underwent distal pancreatectomy with splenectomy and aneurismectomy. In the three other patients ligation of pseudoaneurysm in was performed together with the side to side pancreatojejunostomy derivation.

In these six cases there were no complications after surgery and no mortality.

BT014 HEPATIC RESECTION FOR PRIMARY OR SECONDARY TUMORS: DIAGNOSTIC AND THERAPEUTICAL ASPECTS IN OUR EXPERIENCE

G. Carrella, D. De Anna*, F. Buccoliero, GC. Pansini, P. Carcoforo*, S. Uzzau*, V. Bresadola, F. Bresadola*.

University of Ferrara and Udine*. School of Medicine, Department of Surgery, Institute of "Clinica Chirurgica" and "Chirurgia Generale*". ITALY.

Resectional treatment for primary and secondary liver tumors is greatly increasing in many countries of the world.
The improvement of understanding of hepatic surgical anatomy and new diagnostic techniques have contributed to progress in this field.
Particularly the AA believe that intraoperative ultrasonography offers several advantages to better define the limits of resectability of tumors identifing also small intraparenchimal lesions sometime differently undetectable.
This report analyzes an experience of 46 consecutive liver resections for primary or secundary tumors.
Primary tumors ( 22 cases ) were 19 hepatocellular carcinomas (14 in cirrhotics) ,2 cholangiocarcinomas and 1 hepatoblastoma. ;Type of hepatic resection performed were: right hepatectomy n. 2 (9,1% ),left hepatectomy n. 4(18,2%),trisegmentectomy n. 1(4,5%), bisegmentectomy n.9 (40,9%), segmentectomy n. 5(22,8%), subsegmentectomy n. 1 (4,5%).
24 patients underwent resection of hepatic metastases from colorectal cancer. Types of resectional procedure were: right hapatectomy n. 4(16,5%),left hepatectomy n. 3 (15%),trisegmentectomy n.5(15%),bisegmentectomy n.5(20,8%),segmentectomy n.5(20,8%),metastatectomy n. 4(16,7%).
Overall operative mortality rate was 6,5%(3 cases of hepatocellular carcinomas in cirrhosis).
The AA evaluate longterm survival rate of patients treated according to the stage of the disease,the extent of neoplastic liver involvment and the type of resection performed.
Their series confirm that resectional hepatic procedure offers the prospect of long term survival to a significant percentage of patients.

### BT015 CHRONIC PANCREATITIS - SURGICAL OPTIONS IN 32 CASES

F. Castro Sousa. Coimbra, Portugal

Chronic pancreatitis is a theme of controversy; about diagnostic criteria, prognosis and therapeutic options.

Between 1981-1988, 32 patients (27 men, 5 women) with a mean age of 54±15 years with chronic pancreatitis were surgically treated in our department. Alcohol abuse was present in 26 patients and biliary stones in 11. Jaundice or duodenal obstruction were responsible for surgical treatment in six cases, pseudo-cysts in nine, pseudo-neoplasic lesions of the pancreatic head in three, quilous ascitis in two and resistance to medical treatment in 12. A pancreatic resection was undertaken in 9 patients (3 Whipple procedures, 6 splenopancreatectomies); a Puestow procedure was used in 5 patients, a wirsungoplasty and esfincteroplasty in a case of pancreas divisum; ten patients received isolated biliary tract procedures, associated in two with a gastroenterostomy. Pseudocysts were treated by internal or external diversion, resection or a Puestow operation.

Three patients died in the post-operative period; five presented complications (infections or upper gastrointestinal bleeding). Long term follow-up (25 patients) showed a marked improvement in more than 80% of the patients. One died four years after a Wipple procedure with an unresectable hepatoma.

This study seems to show that surgical options in the treatment of chronic pancreatitis should be the result of careful clinical evaluation of the patient, a criterious interpretation of imaging techniques and of a meticulous preoperative exploration of the anatomic lesions.

BT016 COMPARATIVE STUDY OF PAPILLOSPHINCTEROPLASTY (PSP) AND CHOLEDOCHODUODENOSTOMY (CDS) IN THE TREATMENT OF BILIARY LITHIASIS.

E.A. Chaim; L.S. Leonardi; F. Callejas Neto

Unicamp Medical School,Campinas-SP,Brazil

The authors analysed 40 patients with lithiasis of the common bile duct: 20 were submitted to PSP, and 20 submitted to CDS.

The PSP were employed when the diameter of the common bile duct was approximately 1.5cm, and the CDS was prefered when the diameter was more than 2.0cm.

The transduodenal PSP was performed with a 1.5cm section starting at the papillary ostium, to assure adequate biliary drainage. The supraduodenal CDS was performed using latero-lateral anastomosis, after transverse choledochotomy close to the duodenum, to remove all the biliary stones above the papilla. The postoperative complications in both groups were insignificant.

The late follow up from two to seven years using clinical and laboratory tests of the liver, X-rays and ultrasonography showed that both procedures are efficacions, since the exact indications of each one are respected.

References:

Shaw SJ, Rimmer S. Surg Gynecol Obstet 1987; 164:351.

Bithencourt GV, Benitez SE. Rev Esp Ap Dig 1985;67:517.

Jones SA, Smith LL, Keller TB, Toergenson EJ. Arch Surg 1963; 86:1014.

BT017

### SPLENO-INTRAHEPATIC PORTAL SHUNT FOR PORTAL VEIN OBSTRUCTION

Victor T.K. Chen, J. Wei, Y.C. Liu
Department of Surgery, Tri-Service General Hospital, National Defense Medical Center, Taipei, Taiwan, R.O.C.

A 19 years old male received subtotal gastrectomy for duodenal ulcer perforation. Unfortunally, iatrogenic transection of hepatoduodenal ligment happened in outside hospital. This poor patient was transferred to our hospital, and we finished reconstruction of portal vein with a Gor-Tex graft and cholecystectomy and tube choledochostomy 10 hours later.

The patient developed esophageal variceal bleeding because of Gor-Tex thrombosis. We performed a new technique to resolve the portal vein obstruction, we did splenectomy and mobilized splenic vein to its junction with superior mesenteric vein, and dissected left portal vein branch intraparenchymally, then end to side spleno-intrahepatic portal shunt was made.

The shunt is the most physiologic surgical procedure for treatment of portal vein obstruction.

References:

Warren WD., Millikan WJ Jr., Henderson JM., Rasheed ME., Salam AA., Annals of Surgery. 199(6):694-702, 1984 Jun.
Henderson JM., Millikan WJ., Galambos JT., Warren WD., British Journal of Surgery. 71(10):745-9, 1984 Oct.
Kooiman AM., Kootstra G., Zwierstra RP., Netherlands Journal of Surgery. 34(3):97-103, 1982 Jul.
Fonkalsrud EW., Zeitschrift Fur Kinderchirurgie. 35(2):57-61, 1982 Feb.

BT018

### EMERGENCY LIVER RESECTION FOR RUPTURED HEPATOCELLULAR CARCINOMA

D. Cherqui, N. Rotman, Q.A. N'Guyen Dinh,

M. Kracht, P.L. Fagniez

Service de Chirurgie Générale et Digestive,

Hôpital Henri Mondor, 94010 Créteil, France

Spontaneous tumour rupture is a severe complication of hepatocellular carcinoma (HCC) with a mortality rate over 75% in most series.

From 1983 to 1989, 34 patients with HCC were operated on at our Institution, 30 of whom had liver cirrhosis (88%). Five patients (14%) presented with an acute haemoperitoneum due to HCC spontaneous rupture. All patients had non alcoholic liver cirrhosis. Two patients had right upper quadrant pain during the week preceding the acute rupture. On admission, all patients had abdominal pain and tenderness and were hypotensive.

Emergency laparotomy was carried out in all 5 patients. Liver resection was performed in 4 cases : wedge resection (2 cases), segmentectomy (1 case), and right extended hepatectomy (1 case). One patient died of liver failure 10 days after surgery. Among the 3 other patients, 1 survived 6 months, another 12 months and the last one is alive 2 years after right extended hepatectomy but has now recurrent tumour in his remaining liver. The last patient had a non resectable bulky central tumour. Hepatic artery ligation was performed, but haemostasis could not achieved and the patient died intraoperatively.

These data suggest that emergency liver resection for ruptured HCC is effective in the control of bleeding and may permit prolonged survival. It could be the treatment of choice when technically feasible.

### BT019 SURGERY OF PANCREATIC AND BILIARY CANCER

S. Chikoteev, V. Shanturov

Institute of Surgery, Irkutsk, USSR

During last decade carcinomas of pancreas and biliary system were diagnosed in 176 patients. There ages ranged from 18 to 76 years. Normaly the diagnosis was made by the use of ultrasonography, computed tomography, endoscopic retrograde cholangiopancreatography and angiography. Tumor was located at pancreas head in 94, at body and tail in 34, at main biliary duct in 24, at large duodenal nipple in 18, at gallbladder in 6. Patients with pancreas head and main biliary duct cancer were always manifested as jaundice (84%) and hepatomegaly, otherwise patients with body and tail cancer frequently presented by abdominal pain.

In most of all patients the diagnosis was histologically confirmed by either operation or autopsy.

26% of patients with pancreas cancer were underwent tumor resection. 36 patients with cancer of pancreas head were underwent Whipple's operation. We emphasized for two point during this procedure: didn't removed a pylorus and adjusted outer drainage of pancreatic juice.

The other patients underwent palliative interventions, 13% of them - explorative laporotomy.

Postoperative mortality was 16%.

**BT020** CARCINOMA OF THE GALL BLADDER: CONSIDERATION OF 63 CASES

L. Cimino, D. Iafrancesco, S. Ballatore,
M. Amati, M. Pizzo. Osp San Giacomo,
Div di Chirurgia, USL, Roma, Italia

Carcinoma of the gallbladder is a rare tumour, usually discovered at an advance stage. It is one of the abdominal neoplasia with the most serious prognosis because of its intrinsic invasiveness and the delay in which it is diagnosed. The etiology is unknown although risk factors are taken into consideration: lithiasis (but 1/4 of the neoplasia originate from alithiasic gall bladders), chronic phlogosis, chronic use of some drugs (antidiabetics, diasitics, antihypertensives etc.), ulcerous colitis, several substances used in industry.

Takabayasei and others have discovered a possible connection between insufficient sulphoconjugation of the lithocolic acid normally present in bile and cancer of the gall bladder. Our experience, matured in 15 years (1972-1987) has enabled us to gather 63 cases of cancer of the gall bladder (41 women and 22 men - average age 65.5 years). Our series has brought out some particularly discouraging facts, as 57 cases were at the 5th stage, so the surgical approach was restricted to palliative operations or to a simple exploratory laporotomy plus biopsy. Only 2 cases were at the 1st stage with post-operative histologic diagnosis. 4 cases at the 3rd stage, in which cholecystectomy was performed, a cuneiform resection of the liver and a lymphectomy of the hepatoduodenal peduncle.

Of the 41 patients followed up:
Stage I - 1 patients living to 18 months
Stage III - out of 3 patients, 3 living to 6 months (1 living to 12 months, 0 to 18 months)
Stage V - out of 37 patients, 12 living to 6 months, 8 to 12 months and 0 to 18 months.
Overall mortality rate out of 41 patients: 61% at 6 months, 75% at 12 months, 97.5% at 18 months.
Cancer of the gall bladder, therefore, at a higher stage than the 2nd does not offer possibilities of surgical radicality.
References:
Koo J et al. Carcinoma of the gallbladder. Brit J Surg 68 (1981) 161

BT021

### PANCREATIC ABSCESS

L Cimino, D Iafrancesco, S Ballatore
M Amati, M Pizzo
Ospital S Giacomo, Div di Chirurgia, USL
Roma, Italia

Pancreatic abscess is a rare complication of acute pancreatitis occurring in 1% to 4% of the cases of acute pancreatitis. Pancreatic abscess, if untreated, has a 100% mortality rate which drops to 50% - 30 with an early diagnosis and rapid surgical treatment. In the period 1976-1988 we observed 14 cases of pancreatic abscess out of a total of 209 cases of acute pancreatitis, of which 58 were necrotic-haemorrhagic. The overall mortality rate was 28.5%. Biliary disease was responsible in 4 cases, alcohol in 2, surgical traumas in 4, peptic ulcer in 2 cases, the etiology remaining unknown in the last 2. The cultural test was sterile in 14.2% of cases. In 13 cases closed drainage with tubes and lamina was used, and only in one case suction drainage according to Redon. Post-operative complications arose in 5 cases: 3 re-operations for recurrence of the abscess collection, 1 case of stress ulcer treated with gastric resection, 1 fecal in the right colon treated wtih a hemilectomy of the right colon.

In 1984 we put into practice the following therapeutic protocol: naso-gastric probe, TPN, continuous infusion of somatostatin for 12 days, H2 antagonists, wide-spectrum antibiotics, early laparotomy with drainage of the cavity of the abscess, possible reanimation therapy. We did not note any difference in the mortaility rate, compared to international surveys, between closed and open drainage (laparostomy) of the abscess cavity, as long as an accurate debridement of the abscess cavity was performed.

To conclude, we consider that is is not so much the type of drainage used which modifies the course of the abscess as the very early diagnosis, the accurate localisation of the abscess (ecography, CT), immediate operation, and the continuous and adequate therapy of support and reanimation.

References:
Aranha GV, Prinz RA, Greenlee HB.Pancreatic abscess, an unresolved surgical problem. Am J Surg 1982; 144: 534-538

**BT022** BILIARY LITHIASIS IN YOUNG PATIENTS

J.C.U. Coelho, I.F. Azzolini, T.M. Zanier

F.H. Greca, C.E. Pozzobon, G.V. Artigas

Federal University of Paraná, Curitiba,

Paraná, Brazil

It was performed a retrospective study of 50 patients with cholelithiasis and age between 15and 30 years who had been subjected to surgical treatment. Ten patients (20%) were male and 40 (80%) female. Only one patient had hemolytic anemia. The most frequent symptoms were abdominal pain, nauseas and vomiting. A total of 26 patients had acute cholecystitis (52%) and 24 chronic cholecystitis (48%). The choledocholithiasis incidence was 20%. Postoperative complications occurred only in two patients (4%) and there was no mortality. The high incidency of acute cholecystitis observed in present study is possibly due to diagnosis delay.

BT023

### LIVER SEGMENTECTOMY FOR HCC IN CIRRHOSIS.

D.F. D'Amico, N. Bassi, U. Tedeschi, P. Boccagni, G. Ambrosino, M. Cionfoli.
2nd Dpt of Surgery - University of padova - Italy.

The Authors show in this movie the technique of liver segmentectomy in a patient with cirrhosis (CHILD A G.).

Two small hepatocarcinomas were present in the VIth and VIIth segment of the liver and the treatment of choise was a right lateral sectoriectomy.

Diagnosis making is herein discussed with particular regard to the use of lipiodol, CT scan, angiography and intraoperative U.S.

The surgical procedure was performed directly through the parenchima previous Pringle maneuver.

BT024

### THE KASABACH-MERRITT SYNDROME IN TWO PATIENTS WITH GIANT HEMANGIOMA

D.F. D'Amico, N. Bassi, U. Tedeschi, P. Boccagni, A. Brolese, F. Mangano, V. Terribile, M. Cionfoli, G. Ambrosino.

2nd Dept. of Surgery - University of Padova - Italy.

Of the sixty patients undergoing surgery for space-occupying lesions in the liver from 1984 to 89, five had a giant hemangioma and two of them experienced the Kasabach-Merritt syndrome.

In this rare syndrome (only 155 cases reported up to now), a hemangioma, mostly of the skin and sometimes of the viscera, is associated with thombocytopenia, hypofibrinogenemia, deficiency of coagulation factors II, V, VII, VIII, X and elevated levels od FDP.

Both patients presented with enormous hepatomegaly and laboratory findings included the above-mentioned hematologic changes. However, platelet count, fibrinogen and FDP levels reverted promptly to normal following removal of the hemangioma via a right extended hepatectomy and a right hepatectomy, respectively.

Pathologic diagnosis of both specimens revealed cavernous hemangioma.

The Authors point out the importance of diagnostic procedures such as ultrasonography, technetium-99m scintigraphy, CT, laparoscopy, FNAB for the work-up of this lesion and particularly focus upon the inconsistency of the angiographic study which showed an avascular lesion in both cases.

**BT025** SURGICAL MANAGEMENT OF PANCREATIC PSEUDOCYSTS.

Kr.Daskalakis, T. Mavromatis, E.Anagnostou, C.Kardaras, D.H.Kintzonidis.

"Evangelismos" Hospital, Athens
GREECE

In patients with pancreatic pseudocysts elective operation is performed if the pseudocyst is biger than 4cm and after unsuccessful conservative treatment for 6 weeks.
The choice of operation depends on the size, nature, situation and type of cyst, as well as the general condition of the patient.
Internal drainage of the pseudocyst is prefered.
In the 3rd Surgical Department of "EVAGELISMOS" Medical Center, 25 patients with pancreatic pseudocysts were operated on the last 7 years (1983 to 1989). There were 14 women and 11 men. The age of these patients ranged between 25 to 65 years.
The cause of the pseudocyst was acute biliary pancreatitis in 18 patients, trauma in 2 and unknown in 5.
Five patients with spontaneous regression of the pseudocyst are not included in this study.
Internal drainage was performed in 20 patients: in 4 patients a cysto-gastrostomy and in 16 a cysto-jejunostomy using a Roux-en-Y jejunal loop was performed.
External drainage of the cyst was done surgically in 3 patients because of infection. From these 3 patients, 2 reoperated on after a few months, using a Roux-en-y jejunal loop and the other one has had spontaneous healing of the remaining cyst and the pancreatocutaneous fistulla.
Two distal pancreatectomies without splenectomy were performed.
There were no postoperative deaths in this series.
The postoperative complications, the choice of operation and the follow-up of the patients are discussed.
It is concluded that the drainage using a Roux-en-y jejunal loop, is the teatment of choice.

### BT026 MINOR HEPATIC RESECTIONS IN PATIENTS WITH LIVER CIRRHOSIS.

De Sena G., Darretta G., Sallusto A., Romano V., Cogliolo P.
INSTITUTE OF GENERAL SURGERY (Chief: Prof. G.Zannini).
II FACULTY OF MEDICINE AND SURGERY - UNIVERSITY OF NAPLES (ITALY)

The authors present some cases of minor hepatic resections for hepatocarcinoma on cirrhosis. The technique adopted is by trans-parenchymal way. The vascular exclusion of the liver is seldom indicated. On the basis of a correct diagnostic and therapeutic protocol, the surgeon chooses the kind of resection to perform before operating time. The final evaluation is reached with the peroperative US-graphy. Peroperative blood-loss is the highest risk which in the cirrhotic patient represents the real surgical problem. Selecting the patients for hepatic resection, we therefore excluded those having a high grade of portal hypertension. From the technical point of view, the resection in a cirrhotic patient is performed by clamp-fracture with the Kelly clamps. Vascular and biliar peduncles are tied with slow-absorbable suture. In 6 years, the authors have observed 133 patients with hepatocarcinoma, 112 of these, had a hepatocarcinoma on cirrhosis (84,2%) and 21 on sane liver (15,7%). 26 patients underwent a hepatic resection. 17 had a hepatocarcinoma on cirrhosis (Resection Rate 15%) and 9 on sane liver (R.R. 42%). Surgical mortality is 15% (4 cases) of which 3 for hepatocarcinoma on cirrhosis (17%) and 1 on sane liver (11%).

### BT027 IATROGENIC PATHOLOGIES OF EXTRALIVER BILE DUCTS

L. A. Ender, A. I. Lobakov
Department of Clinical Surgery, Moniki
Moscow, USSR

We have the experience of treating 167 patients with iatrogen extraliver bile duct pathologies (EBD) due to cholecystectomy. The analysis of our material showed that EBD injuries more often than not remain uncovered during surgery, and after operation a number of complications arose demanding complicated rehabilitation and reconstructive operations. The factors contributing to EBD injuries are: organizational, tactical and technical mistakes. Cholagogue surgical correction method selection depends on the extent of the injury and location, as well as on the period of trauma. While restoring EBD integrity in 'fresh' trauma, good results are obtained in plastic ducts on trans-liver drainage, according to Pradery-Smith's method. While performing reconstructive operations, best results are achieved by forming different bilio-digestive anastomoses.

**BT028** SELECTIVE SHUNT VERSUS NONSHUNT SURGERY FOR MANAGEMENT OF BOTH SCHISTOSOMAL AND NONSCHISTOSOMAL VARICEAL BLEEDERS

F A Ezzat, K M Abu-Elmagd, M A Aly
O M Fathy, N A El-Ghawlby, A M El-Fiky
M H El-Barbary
Gastroenterology Unit, Department of Surgery, Mansoura University School of Medicine, Mansoura, Egypt

This clinical study included 219 (Child A/B) consecutive variceal bleeders. Electively, 123 had distal splenorenal shunt (DSRS) and 96 splenectomy and gastroesophageal devascularization (S&GD). Liver pathology was documented in 73% of patients, with schistosomal fibrosis in 41% and nonalcoholic cirrhosis or mixed pattern (fibrosis and cirrhosis) in 59%.

The surgical groups were similar preoperatively with a mean follow up of $82 \pm 13$ and $78 \pm 18$ months respectively (range: 60-120). The operative mortality was low (3.3% for DSRS and 3.1% for S&GD). Rebleeding occurred more frequently after S&GD (27%) compared to DSRS (5.7%, $P<0.05$). Sclerotherapy salvaged 65% of S&GD rebleeders. Encephalopathy developed significantly ($P<0.05$) more after DSRS (18.7%) than S&GD (7.3%), with no significant difference among the current survivors. The difference in overall rebleeding and encephalopathy rates between procedures was statistically related to patients with cirrhosis and mixed lesions ($P<0.05$). DSRS significantly reduced the endoscopic variceal size more than S&GD ($P<0.05$). Prograde portal perfusion was documented in 94% of patients in each group with a variable distinct pattern of portaprival flow. Cumulative survival was similar with 80% for DSRS and 79% for S&GD (+ sclerosis in 23%). Hepatic cell failure was the cause of death in 46% and 50% respectively. However in the schistosomal patients, survival was better after DSRS (90%) than S&GD (75%) with no difference among the cirrhotic and mixed group (DSRS 73%, S&GD 72%).

In conclusion:

1 Both DSRS & S&GD have low operative mortality
2 DSRS is superior to S&GD in the schistosomal patients
3 S&GD backed up by endosclerosis for rebleeding is a good surgical alternative to selective shunt in the nonalcoholic cirrhotic and mixed population.

**BT029** URGENT OR ELECTIVE OPERATION IN THE TREATMENT OF THE ACUTE CHOLECYSTITIS

J. Faller, I. Sugár, P. Ondrejka

Surgical Department of Semmelweis
University Medical School
Budapest-Hungary

In the treatment of the acute cholecystitis the urgent or so called early-operation is gaining more and more preference.

At our department we have preferred the urgent operative treatment of acute cholecystitis to the conservative therapy in the course of recent years.

The experiences of two consecutive two year period were compared. In the period of conservative treatment (first group) 27 patients were admitted with this diagnosis and were treated in conservative way. Out of them in 3 cases urgent operation was necessary within 2-4 days after the beginning of the conservative treatment because of the progression of the illness coming in spite of the treatment. 24 patient recovered without an operation. 15 out of them returned at the appointed time in a so-called "á froid" condition and underwent an operation. In 4 cases out of them an empyemic gallbladder were removed surprisingly (without any clinical or laboratory sign). 2 of them returned in jaundice, 4 suffered from regular cramps between the two hospitalisations.

In the past two-year period 31 patients were admitted with the same diagnosis. 26 patients underwent an urgent operation within 3-24 hours after their admission (second group). The beginning of their complaints was not farther than 72 hours. Besides the patients mentioned above there were 5 others with whom the beginning of inflammation couldn't be determined, that is why conservative treatment was carried out.

We compared the complications and the finnancial effects of the two different methods.

The time of the hospital-stay and being on sick list was the half in the first group as in the second. The same refers to expenses of medicaments which is 20-40 % higher in the second group. The number of the minor wound complications were twice as much in the second group as in the first one.

### BT030 TREATMENT OF RECURRENT AND RETAINED BILE DUCT STONES. TECHNIQUES COMPARED.

R.Fornaro, G.Parodi, E.Belcastro, R.Ferraris

1st Surgical Clinic
University of Genoa - Italy

The results of the treatment of recurrent and retained bile duct stones in 136 patients observed from 1978 to 1987 are reported.

The overall success rates were 87,1% for endoscopic papillotomy, 86,2% for common bile duct exploration and 87,2% for common bile duct exploration with drainage procedure.

In regard to the success of these procedures were evaluated the diameter of common bile duct and the number of stones in the duct.

Although our results do not offer elements of certainty, they would suggest that the following indications are valid.

Endoscopic papillosphincterotomy is major improvement in elderly patients with one or a few stones (success rates 92,3%).

When operative exploration is advisable, we choose common duct exploration; a choledocal T-tube is used in patients with normal sphincterial function and with thin walled ducts and daring suture.

The addition of a biliary-intestinal drainage (choledochoduodenostomy or choledocojejunostomy) provides better results in patients with a dilated bile duct (success rates 93,3%) or with multiple stones (success rates 93,7%).

When the apparatus of Oddi has lost its function, caused by an irreversible inflammatory process, transduodenal papillosphincterotomy is justified.

BT031 THE MIRIZZI SYNDROME

C. Fotiadis, J. Gogas, S. Gardikis,
M. Safioleas, D. Iliopoulos, M. Sechas,
Gr. Skalkeas
2nd Dept of Propedeutic Surgery, Athens
University, Greece

The aim of this study was a discriminating presentation of five cases of Mirizzi syndrome which were treated in our clinic in the last ten years.

This syndrome refers to obstructive jaundice caused by external pressure of the extrahepatic bile ducts due to various causes.

In our cases sizable gallstones in the gallbladder and especially in Hartman's pouch caused obstructive jaundice.

The above cases were treated surgically with excellent results.

The technical, diagnostic and therapeutic approach of the syndrome is discussed.

References:

Evert J,Lubbers C. World J Surg 1983; 780-785
Starling J. Surgery 1980; 88: 737
Christoph D.Becker. AJR ,1984; 143:591-596
Patrick Montefuso. Arch Surg 1983; 118: 1221-1223
Thomas C.Bowel. HPD Surgery 1988;1: 67-76.

BT032

### RIGHT HEPATECTOMY FOR SOLITARY NON-PARASITIC LIVER CYST

A. Fraschini, S. Monetti, R. Riva,
G. Magistretti, M. Arrigo, V. Pizzolato
Chirurgia I, Ospedale S. Antonio, Gallarate
Italy

Solitary non parasitic cyst of the liver is a rare disease, occuring at all ages, more frequently in the fourth to sixth decades of life, with a female to male ratio of 4-5 to 1, involving the right lobe twice as often as the left. Cysts less than 8 to 10 cm rarely cause symptoms and may be detected during ultrasounds or CT scan examinations, or at laparotomy. Symptoms are usually those of an upper abdominal mass, with fullness, nausea and occasional vomiting. Complications of the cysts are rare, and may include haemorrage, rupture or jaundice.

CASE REPORT : a 56-year-old woman was admitted to our Department of Surgery on November 1989 with a four-year-history of solitary non-parasitic liver cyst. The cyst, detected during an ultrasound examination in the sixth segment of the liver, had in 1986 a diameter of 2.5 cm. In 1987 the cyst was asymptomatic with a diameter of 4 cm. A sudden widening of the upper abdomen, associated to a fullness sensation began in September 1989. On November 1989 ultrasound examination and CT scan showed that the cyst was completely filling the right lobe,with a round-oval shape and a maximum diameter of 24cm, compressing the IVC and pushing the aorta to the left.A right hepatectomy was made using a right subcostal incision extended to a phrenothoracotomy. The cyst was filled with 4.5 liters of clear fluid. A small portion of the cystic wall fixed to the IVC had to be left in place. Uneventful postoperative course. The patient left the Hospital 14 days after surgery. Histology confirmed the diagnosis.

Mayor hepatic surgery for cystic disease is erroneusly thought to be a too big operation for a benign disease. In fact, mortality rates are less than 5 percent in large series of hepatic resections. Moreover, resective surgery has a lower morbidity rate than non radical surgery.

**BT033** RADICAL SURGERY FOR HYDATID CYST OF THE LIVER

A Fraschini,G Taborelli,M Arrigo,G Giani

Chirurgia I,Ospedale S.Antonio, Gallarate
ITALY

In recent years,surgery for hydatid cyst of the liver has shown a clear trend towards radicality. Total pericystectomy and limited hepatic resection have become the most used operations in many centers.

Mayor hepatic resections have been only occasionally performed.[1-2]

In our Department of Surgery, all the 25 cases of echinococcal liver disease operated since 1974 have been treated with radical surgery. Twenty-four total and one subtotal pericystectomies in 21 patients, three atypical resections and one right hepatectomy were made.
There was no mortality and a low morbidity. We found one biliary fistula, one haemorrage and three wound infections, all treated conservatively. Another infection, of the residual cavity, was successfully treated with percutaneous drainage. Mean postoperative hospital stay was 12 days. At present, no cases of recurrent disease have been found. The very good results observed, clearly suggest that there is no place, except in very particular cases, for the non radical surgery in the treatment of hydatid cyst of the liver. This because non radical surgery has higher morbidity and recurrence rates, with similar mortality.

1) G Gozzetti,A Cavallari, R Bellusci, A Mazziotti, B Nardo, E De Raffele, A Galasso. First Congress of Ital. Chapter. World Assoc Hep Panc Bil Surgery ESSERRE ED. Bologna 1988 pag 15-18.
2) G Belli, G Romano, A Monaco, V D'Ambrosio,LM Santangelo.
First Congress of Italian Chapter- World Association of Hepato Pancreato-Biliary Surgery. ESSERRE ED. Bologna 1988 pag 533-536.

### BT034 "OPEN TREATMENT" FOR ACUTE NECROTIZING PANCREATITIS

A. Fraschini, G.Carcano, L. Dominioni,
S. Monetti, R. Dionigi, G. Taborelli
Chirurgia I, Ospedale S. Antonio, Gallarate
Italy

Mortality rate for acute necrotizing pancreatitis is still very high, despite the great progress of the intensive care, especially in the very last years. This high mortality rate is frequently caused by severe sepsis, still present despite extensive debridement, peritoneal lavage and drainage have contributed in reducing complications.

The open treatment of acute necrotizing pancreatitis, with daily reexploration and lavage of the open abdomen, is a method proposed by several Authors in recent years. We have used this method in three patients with histologically confirmed acute necrotizing pancreatitis. The open treatment was made in all three cases as second operation. A severe generalized sepsis was present, at 2, 3 and 5 weeks after the first operation. The sepsis was due to multiple large collections of pus in the peritoneal cavity and in the retroperitoneum. Careful and wide debridement of these areas and some necrosectomies were made, a peritoneal lavage was done and multiple drainages inserted. The abdominal wall was temporarily closed with a plastic zipper suture (ETHIZIP°).

The patients were sent to the Intensive Care Unit, where daily exploration with zipper opening and debridement of pancreatic necrotic tissue, fibrinous adhesions and inflammatory secretions, was made. When systemic and local signs of sepsis had disappeared, the zipper was removed and the abdominal wall closed with one-layer interrupted nylon stitches (on 9th,11th and 12th postoperative day). All three patients survived. The mean stay in Intensive Care Unit was 14 days, and postoperative hospital stay was 34 days.

In the severe intra-abdominal sepsis the open treatment with zipper permits a better drainage of the abdomen, multiple reexplorations and lavages of the peritoneal cavity. The use of the zipper avoids evisceration and reduces the need of respiratory support.

### BT035 CELL KINETICS AND CELLULAR MEMBRANE FUNCTION OF RAT LIVER FOLLOWING PORTAL BRANCH LIGATION

K.Fujita, J.Tanaka, J.Tamura, M. Yoshida, T.Kasamatsu, S.Arii, T.Tobe
First Department of Surgery, Kyoto University School of Medicine, Japan.

It is well known that regeneration of nonligated lobe following portal branch (PB) ligation occurs as well as after partial hepatectomy. This phenomenon is applied to clinical field such as transhepatic PB embolization before major hepatic resection. The present study was conducted to investigate cell kinetics and plasma membrane fluidity of hepatocytes after PB ligation. Male Wistar rats weighing 200-250g were used. Ligation of PB corresponding to about 70% liver volume was performed, followed by liver samplings in a given time course. For cell kinetics, after a pulse labelling by 5-bromo-2'-deoxyuridine (BrdU), hepatocytes were isolated by collagenase perfusion method via inferior vena cava. And then, the isolated nuclei were used for flow cytometric analysis using two-color staining of anti-BrdU antibody and propidium iodide. Liver plasma membranes were isolated by centrifugation and fluidity was estimated by measuring fluorescence polarization using 1,6-diphenyl-1, 3, 5-hexatriene as a probe dye. Results; The 2C:4C ratios changed from 0.4 of control to 1.2, 2.2, 0.7, 0.3 in ligated lobe, by contrast, in nonligated lobe, the ratios were 1.2, 0.6, 0.3, 0.4 on 1, 3, 7 and 16 days after ligation. Whereas, proportion of BrdU positive nuclei (S-phased cells) appeared in 5.4, 5.5, 14.7, 2.1% in ligated and 10.2, 9.3, 16.7, 4.4% in nonligated lobe in the given time course. Membrane polarization changed from 0.185 of control to 0.202, 0.212, 0.232 in ligated and 0.165, 0.171, 0.176 in nonligated lobe on 1, 2, 3 days, followed by rapid normalization on 5 days. Above results suggest that the ligated lobe having decreased membrane function atrophies together with restoring process, while the nonligated lobe gradually regenerates.

**BT036** PTFE SMALL DIAMETER PORTOCAVAL "H" GRAFT IN THE TREATMENT OF VARICEAL BLEEDING. TECHNIQUE AND LONG TERM RESULTS.

JL Garcia Sabrido, JR Polo, E Valdecantos, J Calleja, A Quintans, R Bañares, G Clemente, A Fernadez-Pacheco.
Hospital General "Gregorio Marañon" Madrid. Spain

Since 1982 39 corrhotic patients ( 30 emergency and 9 elective ) have been operated on using a portocaval laterolateral shunt by "H" interposition of a small diameter ( 10 and 8 mm ) ringed PTFE graft.
This video shows all technical aspects of the differents steps of the operation: dissection of the vascular structures, interposition of a short and small diameter segment of ringed PTFE graft, vascular anastomosis, taking of portal pressures and comprobation of the shunt patency by means of echo-doppler studies and transfemoral portography.We don't use portal collateral ligation to complete this operation.
The analysis of the present serie shows that the operative mortality rate ( in hospital mortality) was 0 % in elective patients and 16 % in emergency setting. The portal liver perfusion was proved in 72 % of the cases. The cumulative survival rate was 50 % at 3 years and 40 % at five years and 27 % at seven years. The encephalopaty rate was 18 %. There was 2 early graft thrombosis. The long term patency of the shunt was 95 %.

**BT037** PANCREATIC FISTULAS FOLLOWING SURGERY NECROTIZING PANCREATITIS.

P.Hernandez, JL Garcia Sabrido, JR Polo, LG Bayón, M.Lasala, J. Calleja. A. Quintans, J.Ferreiroa.

Surgery of patients with Necrotizing Pancreatitis(NP) is often followed by many complications. The postop-perative pancreatic fistula (PF) is one of the most frecuent. Since 1982 we have rewieved 42 patients operated on because of NP. Surgical treatment was based on sucesive necrosectomies and zipper laparostomy. The abdomen was closed when granulation tissue developed in the retroperitoneum. Two or three drains were placed in pancreatic area to control the infected zone. In 17 patients(41%) postoperative PF,defined as presence of persistent fluid with more than 1000 U of amylase during one week,was detected. The cornerstone of treatment in PF was a complete drainage of pancreatic juice , total parenteral nutrition and intravenous infusion of somatostatin. The mean duration of the fistulae was 2.9 weeks. The spontaneous closing after medical treatment occurred in 14 patients(82.3%).Two patients died(11.7%),one after closing of the fistula.Two patients underwent surgical procedure because a chronic fistula. We conclude that PF is a frecuent complication following surgery of NP,but in the majority of cases the closing of the fistula can be achieved by consevative medical treatment.

BT038 AN UNUSUAL INDICATION OF ENDOSCOPIC PAPILLOTOMY

M Gergely+, Cs Csonka+, Z Seress++
County Hospital
+ 1st Department of Surgery
++ 2nd Medical Department
Szolnok, Hungary

The era of cold-light flexible endoscopy basically changed the surgical diagnosis. As a next step, operative endoscopy opened up new vistas. Routine endoscopic papillotomy (EP) is an everyday tool practically all over the world. In the following case EP was successfully used for an unusual and probably unique indication.

On the 9th of May 1989, we operated upon a 33 year old male patient for a large echinococcal cyst, which was invading and occupying the entire right lobe of the liver. The removal of the cyst practically corresponded to a right lobectomy. After the removal of the gallbladder and after division and ligation of the main anatomic structures of the right lobe, bile leakage was observed on the remaining biliary tract and was meticulously closed by atraumatic stitches. On the 6th postoperative day a considerable bile discharge developed through the drain. Though the patient did well, was not jaundiced at all, and even had normal stools, the fistula proved to be resistant to all conservative efforts. On the 38th postoperative day we requested a papillotomy. The ERCP was easily performed, and an endoscopic papillotomy was carried out. Though the contrast material did not show any leakage, from the next day following the papillotomy the biliary fistula abruptly closed, and the patient was discharged home. In the course of the follow-up his physical examinations, lab tests and ultrasonography were perfectly normal, and corresponded to the operation.

We believe, that decrease of the biliary pressure to a subnormal level by division of the sphincter mechanism was the cause of the immediate success, so we believe in similar cases endoscopic intervention could be the method of choice, but much earlier than we decided.

BT039 EARLY CHOLECYSTECTOMY FOR ACUTE CHOLECYSTITIS; INFLUENCE OF DURATION OF SYMPTOMS

F.H.A.M. Hoppenbrouwer, D.J. Gouma.
University Hospital Maastricht, The Netherlands.

Cholecystectomy is still the treatment of choice for acute cholecystitis. The timing of surgery, early or delayed, is however controversial and also depending on duration of symptoms (48 hours). The aim of this study was therefore to evaluate the results of early cholecystectomy for acute cholecystitis and the influence of duration of symptoms on morbidity and mortality. In a three year period 122 patients (F: 69; M 53) underwent "urgent" cholecystectomy which was 24% of the patients surgically treated for gallstones. The mean age was 64 years (range 29-101 year). Two patients ( 88;89 years) died respectively 24 and 26 days postoperative from cardiac complications. Morbidity according to the duration of symptoms is summarized in Table I and no difference between groups was found.

| Duration of symptoms | number | Specific compl. biliary | wound inf. | card/pulm compl | mortality |
|---|---|---|---|---|---|
| < 48 hours | 64 | 4 | 6 | 7 | 0 |
| > 48 hours <7 days | 43 | 7 | 3 | 5 | 1 |
| > 7 days | 15 | 3 | 2 | 3 | 1 |
| Total | 122 | 14 | 11 | 15 | 2 |

In conclusion early cholecystectomy is a relative safe procedure especially for patients under the age of 80. The morbidity is not significantly increased with duration of symptoms (48 hours versus 2-7 days) and early cholecystectomy can also be considered for patients which, symtoms for more than 48 hours.

**BT040** SURGICAL MANAGEMENT OF CHOLEDOCHAL CYSTS.

G.Gozzetti, A.Principe, M.Spangaro, A.Mazziotti
2° Dept. of Surgery, University of Bologna - Italy

Cysts of the extrahepatic biliary tract are usually detected during childhood, but even in adult life they are a problem to manage.

Since symptoms are aspecific, diagnosis was made only at laparatomy whenever if surgical indication was advanced.

Modern diagnostic such as routine ultrasound studies, ERCP, PTC, CT scan, consent an accurate diagnosis and surgery can be programmed to avoid the complications of untreated cysts such as lithiasis and malignant degeneration.

The Authors present a small series of six biliary cysts in adult patient, classified (according to Todani and Watenabe classification) as three type I, two type IV and one type V. In five patients stones were present in the cyst and in one case histology revealed a carcinoma in the posterior wall of the cyst.

This videotape present shows the management of two type Ia cases, one complicated by lithiasis and the other by cancer.

Cholangiography and intraoperative echography consent a very suggestive iconography.

The procedure consists in the complete removal of the cyst and according to us always be done wherever the lesion is located. Reconstruction is carried out with a bilio-digestive derivation with a wide anastomosis on normal tissue at superior biliary confluence level to avoid complications and consent the passage of eventual intrahepatic stones.

**BT041** INFLAMMATORY PSEUDOTUMOR OF THE LIVER : CASE REPORT.

F. GRAUSO; L. ANTOGNOLI; A. PICCIUTO; L. ZANELLA CAVALLERO; R. PASQUALI LASAGNI; N. CAMPIONI

Dept. of Surgical Oncology - "Regina Elena" Cancer Institute - Rome - Italy

The AA. report on a rare case of inflammatory pseudotumor of the liver observed in 1989 on an overall casistic of 717 liver tumors ( 563 primitive or methastatic and 154 benignant neoplasms ) treated between 1980 and 1989 at the 2nd Dept. of Surgical Oncology of the "Regina Elena" Cancer Institute of Rome (Italy).

Analysis has been made on etiopathogenesis and results of surgical therapy looking for common point with the other few cases reported in Literature.

BT042 SURGICAL TREATMENT OF PURULENT COMPLICATIONS OF THE LOCAL-SPREAD PANCREONECROSIS

E Grigoriev, V Park, S Kolmakov
Institute of Surgery, Irkutsk, USSR

32 patients with the local-spread purulent pancreonecrosis complications were examined. In 14 cases this disease developed after hard pancreas injury (with the injury of the pancreatic duct in 9 cases) and as a result of cholelithiasis in 18 cases.

In most cases retroperitoneal phlegmon was observed to spread in the left and in 4 cases in the right pericolitis fat; in 2 cases the total lesion of paranephric fat was observed on the left side.

In 17 cases distal resection of the pancreas was implemented (in 2 of them there was a subtotal left-side resection).

15 patients were subjected to polyphocal irrigation-evacuation drainage of paranephric and pericolitis spaces.

In all cases the extensive necrosequestrectomy was conducted during the operation. The obligatory operative component was either external or internal drainage of biliary ducts.

9 patients died in the group under examination (28.12%).

The mortality was mainly caused by generalised intraperitoneal infection and peritoneal sepsis.

### BT043 ULTRASOUND DIAGNOSIS AND CHOLELITHIASIS.

G.Grunblatt; G.Quintero; M.León.

Hospital M.Perez Carreño, Cirugía III

Caracas, Venezuela.

Over a period of three years, 1986-1988, we studied 60 patients from our Surgical Emergency Room. These patients had no previous diagnosis of biliary pathology. However, upon admission, after a through clinical examination and laboratory evaluation, we diagnosed them as "biliary emergencies". An abdominal ultrasound was performed, with special emphasis upon the gall bladder and adyacent structures. This procedure was done by associates of the Gastroenterology Department, none by their residents.

All sixty patients were taken to the opreating room within 24 hours. Intra operatively, the ultrasound diagnosis proved to be correct in 55 cases (91.6%); and according to the report of the Surgical Pathologist, the diagnostic by ultrasound was correct in 51 cases (85%).

This is a small series, and we are accustomed to read results of much larger series with higher percentages of accuracy; however,we do not always have this facility at our public hospital, and when we do , it does decrease the level of uncertainty when operating an emergency patient.

BT044

### BILE PROOF SIDE TO SIDE CHOLEDOCHODUODENOSTOMY (CDD)

M.N. Gyras,K. Karassavas,A. Angelis,N. Sias,
K. Tsolias,A. Papathanassiou and H. Louis

The Asclepeion Red Cross Hospital,
Voula,Athens,Greece

In this paper we describe the operative technique we used in 100 selective cases over the last 4 years.

100 patients were operated upon for benign obstruction in the common bile duct.There was a male-female ratio of 1:2 and the age of the patients varied between 90 and 48,the mean age being 68.5 years.

We used 3.0 vicryl stitches,6 stitches all in one layer.The width of the cut on the common bile duct is 1.5 cm. and that of the duodenum about 0.7 cm.The "bite" of the stitch on the duodenum does not include the mucosa of the duodenum-a point we think is very important.We take every precaution to avoid placing any tension on the anastomosis.Through a stab wound we drain the area with a soft flat rubber drain.

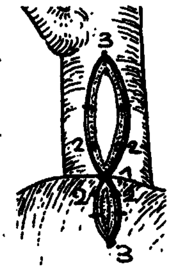

Out of the 100 patients only 4 had any perceptible leakage of bile,and this for only 2 days;we were able to remove the drain on the 3rd day.In the remaining 96 patients the drain was absolutely dry.The mean stay at the hospital was 6 days, and there were no deaths or postoperative complications of any kind

The patients were followed up and questioned about their feelings.92 are well and free of any symptoms and 8 have mild atypical dyspeptic symptoms .No postoperative cholangitis was reported.Intravenous cholangiography,ultrasound studies and a barium meal in these 8 cases showed no pathology to account for their mild symptoms.

We are satisfied with the results of our technique,which is fast,simple and,most important bile proof.

Baker A. Ann. R. Col. Surg. Eng. 1987 V. 68. 253-257

**BT045**

### ENDOSCOPIC MANAGEMENT OF COMMON DUCT STONES WITHOUT CHOLECYSTECTOMY - 5 YEAR FOLLOW-UP

J.Hill, D.F. Martin, D.E.F. Tweedle

Departments of Surgery and Radiology, Withington Hospital, Manchester M20 8LR. UK

Category 13.

There have been numerous and conflicting reports of the short term follow-up of patients undergoing endoscopic choledocholithotomy (EC) with their gall bladder in situ. We have followed 91 patients (36 men, 55 women, mean age 76 years) for up to 8 years (minimum 5 years) who underwent EC between January 1980 and December 1984. Other medical conditions were present in 36 patients; most commonly previous myocardial infarction or stroke (19), malignancy (7) and diabetes mellitus (5).

The duct was cleared in 79 patients (87%). Five had elective cholecystectomy performed at the referring hospital. Of the remaining 74 patients, 5 have been lost to follow-up leaving 69 patients followed up for a minimum of five years. The deaths and patients requiring cholecystectomy for complications or symptoms related to the gall bladder is shown below for each of the five years.

| Follow-Up (Months) | Number of Deaths | Cumulative Deaths % | Number requiring svmptomatic cholecystectomy |
|---|---|---|---|
| 1- 12 | 13 | 18.8% | 4 |
| 13- 24 | 4 | 24.6% | 0 |
| 25- 36 | 7 | 34.8% | 0 |
| 37- 48 | 8 | 46.3% | 0 |
| 49- 60 | 7 | 56.5% | 1 |
| Total | 39 | 56.5% | 5 (7.2%) |

The causes of death were myocardial infarction or heart failure in 12, malignancy 10, respiratory failure 4, stroke 4, others 8. No deaths were attributable to gall stones. Only 5 (7.2%) required symptomatic cholecystectomy. 26 patients remained alive and well with their gall bladder in situ at 5 years. Mean additional follow-up of these 26 patients beyond 5 years is 17.5 months. Two of these patients have died from unassociated causes and none have symptoms related to the gall bladder.

In conclusion, elective cholecystectomy is not warranted in elderly patients with symptomatic common bile duct stones if the common duct can be cleared of stones by endoscope sphincterotomy.

**BT046** SIMULTANEOUS PANCREATICODUODENAL/ RENAL TRANSPLANTATION

U T Hopt, M Busing
Department of Surgery, University of Tubingen, FRG

The operative technique of pancreatic transplantation is still a matter of debate. In respect to the management of exocrine pancreatic secretion, the bladder drainage technique seems to be most reliable. On the other hand, however, the donor operation as well as the recipient operation seem to be more complicated with this new technique. Combined pancreaticoduodenal/renal transplantation with bladder drainage in the modification of Corry has been performed in 27 type I diabetics with end stage renal disease at the Department of Surgery, University of Tübingen. The perioperative complication rate was very low. Long term graft function rate of the kidney as well as the pancreas is excellent. In the first part of the video the donor operation is shown. After dissection of the celiac axis, the superior mesenteric artery and the hepatoduodenal ligament, the whole pancreas and the spleen are removed en bloc. Splenectomy, final dissection of the vessels and preparation of the blind short duodenal segment are performed during bench table surgery. Finally, revascularisation of the pancreas, the anastomosis between the duodenal segment and the urinary bladder and the simultaneous kidney transplantation are shown.

BT047 THE TECHNIQUE OF PANCREATICO-DUODENECTOMY (LESSONS LEARNED WITH 176 PATIENTS)

J. Howard
Medical College of Ohio, Toledo, Ohio, USA

Personally having performed 176 Whipple resections of the pancreas, the author's technique has gradually been modified. The changes have centered around more radical lymph node dissections in patients with pancreatic cancer. Following the experience of Japanese surgeons, nodal dissection is carried out along the celiac artery, superior mesenteric vessels, all vessels of the hepatic hilum, the inferior vena cava and the abdominal aorta. Evaluation of long term survival is underway.

Meticulous anastomosis of the pancreatic duct, per se, to a small opening in the Roux Y has been performed over a small polyethylene catheter which is brought to the skin for drainage. In the 32 patients in which this technique has been utilized, there has been no postoperative fistula.

**BT048** PARTIAL PANCREATIC DUCT OCCLUSION WITH PROLAMINE FOR PREVENTION OF PANCREATIC FISTULA AFTER DISTAL PANCREATECTOMY

T.Itoh, Y.Idezuki, T.Konishi, K.Shimada, Y.Nomura, K.Shimomura, Y.Ishizaki
Second Department of Surgery, Faculty of Medicine, University of Tokyo, Tokyo,Japan.

In order to prevent pancreatic fistula after distal pancreatectomy, we deviced a new method, that is, partial occlusion of the main pancreatic duct and its small ductules with prolamine.

After division of distal pancreas, partial pancreatic duct was occluded with 0.2 to 0.3 ml prolamine. The main pancreatic duct was ligated, and then, the divided cut surface of the pancreas was closed interrupted sutures. This method was performed in 40 cases of gastric malignancies ( prolamine-group ). This prolamine-group was clinically compared with other 30 cases without prolamine injection ( Non-prolamine-group ).

Regarding the incidence of postoperative pancreatic fistula, prolamine-group(7.5%) was significantly lower than that of non-prolamine-group(36.7%). In prolamine-group, postoperative hyper-amylasemia appeared in all cases. Localized and transient pancreatitis must have developed in accordance with the part of pancreatic duct occlusion. No operative complications due to prolamine injection was occured. This technique of prolamine-occlusion, which has advantages of safety and simplicity, is considered useful for the prevention of pancreatic fistula.

BT049

### THE SURGICAL SIGNIFICANCE OF THE OCCLUSION OF THE SPLENIC VEIN, CONSEQUENTLY ENLARGED SPLENIC ARTERY CAUSED BY CHRONIC PANCREATITIS OR CARCINOMA OF THE PANCREAS

F Jakab, Z Ráth, O Eigner, Gy Illés
I Sugár
Semmelweis Medical University
Departments of Surgery, Internal
Medicine & Pathology, St. John's
Hospital, Budapest, Hungary

The most common cause of occlusion of the splenic vein is pancreatic disease such as chronic pancreatitis or carcinoma of the pancreas. The chronic pancreatitis and the carcinoma of the pancreas may involve not only the splenic vein but also eventually the splenic artery. Therefore the clinical features of occlusion of the splenic vein may be profoundly altered according to the nature of the underlying causes. In an attempt to clarify the pathophysiologic findings and hemodynamic mechanism of occlusion, the splenic vein associated with chronic pancreatitis or carcinoma of the pancreas, five patients were selected from our past experience.

Upon analyzing the clinical course of these patients three consecutive phases may be distinguished:

Phase 1 is the insidious or latent phase: The splenic vein is partially occluded and gastric varices has not developed. The splenic artery is already and has become wide and tortuous. Development of ascites is the characteristic clinical symptom.

Phase 2 is the collateral developing phase: The splenic vein is completely occluded, the splenic artery is abnormally wide and tortuous, resulting in marked varices and splenomegaly and massive gastrointestinal bleeding.

Phase 3 is the vanishing phase: The occlusion of the splenic artery is superimposed on the occlusion of the splenic vein causing gastric varices to vanish and the enlarged spleen to shrink.

The five patients are demonstrated, representing all of these phases by angiography, CT, ultrasound, intraoperative findings and finally by histological evidences.

BT050 ACUTE NECROSING PANCREATITIS

I M Jekić, M Lj Jekić
Surgical Service, Clinical Hospital
Centre, Zemun-Belgrade, Yugoslavia

The authors describe the procedure which in their opinion should be adopted in cases of acute necrosing pancreatitis.

They base their conclusions on 14 cases which they report, drawing attention to the clinical and biological signs which arouse suspicion of pancreatric necrosis and therefore indicate operation.

As regards the choice of operation, they prefer sequestrectomy of the necrotic tissue, associated with one of the standard methods of biliary decompression, to formal resection of the pancreas.

The mortality, nevertheless, is high, exceeding 50%. The morbidity, which is also high, is mainly due to pancreatic fistulae, which as a rule heal spontaneously.

References:
Bockus H L, Kalser M H, Roth J L A, Bogoch A L, Stein G
Clinical features of acute inflammation of the pancreas. A M A Arch Int Med, 96: 308, 1985

BT051 **CLINICAL ASPECTS OF PYOGENIC AND AMEBIC ABSCESSES OF THE LIVER**

M. Kaburagi, H. Kogure, Y. Tajima
Department of Surgery, Dokkyo University
School of Medicine, Tochigi, Japan.

In spite of marked improvement in diagnosis, pyogenic or amebic abscess of the liver still offers poor prognosis. We have analyzed the records of 12 patients with liver abscess in terms of diagnosis and treatment.

Over a 12-year period since 1978, 12 patients with liver abscess were admitted in our department. Eleven were pyogenic and one amebic. There were 9 men and 3 women. Their average age of the patients was 51.3 years. Diagnosis was correctly made in 11 cases (91.7%). The source of infection was biliary in 6( malignant in 3 cases ), cryptogenic in 3, appendicitis, iatrogenic and amebic in one respectively. The abscess was confined to the right lobe in 9 patients, the left lobe in one and both lobes in 2. Eight were single and 4 were multiple. The commonest presenting symptoms were fever, abdominal pain and hepatomegaly. Bacteriological data were available in 9 patients. Positive cultures were obtained in 7 cases. The organisms identified were Escherichia coli, klebsiella, serratia and so on.

Treatment included percutaneous abscess drainage in 7 cases, surgical drainage in 5, left hepatic lobectomy in one and antibiotics alone in one. All patients with benign diseases are currently alive and well except one. Three patients died of malignancy within 95 days after initial treatment.

In conclusion, early diagnosis and appropriate treatment which involves the use of percutaneous drainage and/or transperitoneal surgical drainage with broad spectrum antibiotics remain the prerequisites for cure.

**BT052** GLUCOSE TOLERANCE AND ULTRASTRUCTURAL CHANGES OF SEGEMENTAL AUTOTRANSPLANTED PANCREAS IN DOGS

H. Kaji, K. Inoue, M. Yun,

*O. Midorikawa, T. Tobe

1st Department of Surgery and

*1st Department of Pathology,

Faculty of Medicine

Kyoto University, Kyoto, Japan

This study was conducted to clarify the relationship between ultrastructural changes of islets of Langerhans and glucose tolerance in the segemental autotransplanted pancreas in dogs. After total pancreatectomy, splenic lobe of the pancreas was transplanted to the left iliac fossa with vascular anastomosis. Pancreatic duct was opened to the peritoneal cavity. At 3, 5 and 7 weeks after transplantation, intravenous glucose tolerance test (IVGTT), insulin response test (IRI).and open biopsy of the transplanted pancreas were performed in the same dogs for ultrasruractural exmination.

IVGTT revealed that at 5 or 7 weeks after transplantation, insulin re^ponse had tendency to be delayed, and biphasic reaction was observed, in spite of the slight deterioration of glucose tolerance. Fibrosis and destruction of exocrine pancreas was seen in the post 5 or 7 weeks transplanted pancreas by light microscopy. Electron microscopical examination demonstrated that islets of Langerhans were invaded by the collagen bandle with remarkable diminution of granules of B-cells. Whereas granules of A-cells were well maintained even at 7 weeks after transplantarion.

In conclusion, this study suggests that slight deterioration of glucose toleranse and low insulin response after segmental pancreatic autotransplantation might be ascribed to the fibrosis of pancreas, decrease of granules of B-cells, and preservation of granules of A-cells.

BT053 **DIAGNOSTIC AND THERAPEUTIC APPROACH OF VERNER-MORRISON SYNDROME. ON OCCASION OF ONE CASE**

**A. Kakouris, S. Karentzos**
Surg.Clinic Hosp.of "Patisia" and
1st Surg.Unit Gen.Hosp."Asclepieion",Athens

The description of Verner-Morrison syndrome was published in 1958. Since then no more than 90-100 new cases have been published. Although the entity is thought to be rare, we believe that there is a real difficulty to understand and diagnose that. Some times the oncoming sickeness is misdiagnosed as ischemic colitis, various types of inflammatory enteritis or malabsorption disease.

Our case was a female 57 years old with history of sudden onset of severe diarrhea, colic pain experienced in the lower abdoment and loss of body weight. Laboratory tests, afterwards, gave us severe hypokalemia and elevated serum vasoactive Intestinal Peptid (V.I.P). A tumor in the tail of pancreas was discovered by means of U.S., C.T. and E.R.C.P. A left pancreatectomy and splenectomy was performed by the second of the authors. The recovery was aneventfull (histopathology, radioimmunoassay and anosoimmunoassay of the tumor will be discussed in detail).

**Conclusions:** Verner-Morrison syndrome is a rare or difficult to diagnose entity. Since diarrhea as "pancreatic cholera", achlorhydria, hypokalemia and hypercalcemia is indicated; the possible diagnosis must be bear in mind. Elevated titles of vasoactive Intestinal Polypeptide (VIP) in serum, U.S. and C.T. can confirm the diagnosis. Left pancreatectomy is the operation of choise in case a single adenoma is founded in the body or tail of the pancreas, while near total pancreatectomy must be performed in the case of multiple tumors, if it is possible. In case of malignancy (40%), a paliative therapy be means of antiprostaglandine drugs may be of value.

### BT054 ASSESSMENT OF BILE REGURGITATION OF HUMAN IN EXTRAHEPATIC OBSTRUCTIVE JAUNDICE

C-G Ker, J-S Chen, K-T Lee, P-C Sheen
Division of Hepatobiliary Surgery
Kaohsiung Medical College Hospital, Taiwan, ROC

A clinical investigation with 1% of bromosulfaphthalein sodium (BSP) was conducted selectively by injection through a choledochoscope with the pressure of 50 cm $H_2O$ into the intrahepatic cuct of patients with severe obstructive jaundice. After 5 minutes, the existence of BSP was detected in the peripheral vascular system. In severe obstructive jaundice, the concentration of BSP ranged from 6.2% to 11.5% and less than 1% in non-jaundiced patients. In addition, a contrast media, Urographin (45%) was given by intracholedochal injection into the same branch and a series of cholangiograms were taken. This contrast media was then found in the parenchyma and vessels, and then disappeared. Such a finding may indicate the existence of a direct communication between the circulatory blood system and the biliary system in severe obstructive jaundice.

Another thiryt-seven patients were studied on the anatomic routes of bile regurgitation by electron microscopy. The aim was to identify the relationship between the clinical results and pathway of bile regurgitation. These patients were classified into 3 groups; recovered, delayed, and fatal. Transcellular, paracellular, direct communication, necrotic hepatocyte and ruptured ductule pathway were found. In the recovered group, the transcellullar pathway was most frequent with an incidence of 54.17%, and a direct communication pathway was found to be 54.55% in the fatal group with a significant difference between that of the recovered and delayed group. Long-duration bile duct obstruction created bile regurgitation by way of direct communication between the canaliculi and Disse's space, and usually with a poor prognosis, since the serum bilirubin reached the high level of 22.65 mg%. Therefore, it is better to relieve the intrabiliary pressure as early as possible to prevent the jaundice from producing the irreversible change of hepatic canaliculi and Disse's space.

**BT055** A VERY RARE CASE OF LOWER COMMON DUCT STRICTURE WITHOUT BILE DUCT INJURY DUE TO ABDOMINAL BLUNT TRAUMA

I.KONISHI, T.HIRONO, N.TAKAYANAGI
TOYAMA CITY HOSPITAL,TOYAMA,JAPAN

We experienced a very rare case of lower common duct stricture without bile duct injury due to abdominal blunt trauma. A 40-year-old man run into traffic trouble and get a slightly abdominal bruise. After 1 week, he visited our hospital with the chief complaint of jaundice. Ultrasonography and computed tomography were carried out directly after admission, and revealed lower common duct obstruction with no space occupying lesion in the pancreas head. Tapering-type obstruction just as the pancreas head cancer was found in the lower common duct on PTCD cholangiography, but not found bile duct injury. Furthermore, microscopic examination of the bile duct on ERCP showed no malignant cell. Neither superselective gastroduodenal arteriography nor portography showed an arterial encasement or tumor staining in the pancreas or bile duct. Laparotomy revealed neither any abdominal bleeding nor particular findings of trauma or neoplasm. Therefore, judging the condition to be malignant neoplasm, pancreaticoduodenectomy was done. Histopathological examination of the resected tissues revealed a collagen fiber increase, inframatory cell infiltration and hemosiderosis arround the localized lower common duct only, and revealed no significant findings in the duct wall nor pancreas tissue.

**BT056** OPERATIVE TREATMENT OF ACUTE NECROTIC PANCREATITIS UNTER THE OPERATIVE PROCEDURE "WIDE DRAINAGE OF THE ANTERIOR SURFACE OF THE PANCREAS TO THE FREE PERITONEAL CAVITY"

A.Koussidis,M.Apsokardou
Department of Surgery General Hospital Chios

The goal of this operative technic is to treat the acute pancreatitis.This operative technic includes:The suction of ascites,we then surgicly free the two curvatures of stomach(greater and lesser)from the pyloric sphincter to the mid of the stomach body.After that we go on and separate surgicly the posterior wall of the stomach from the anterior surface of the pancreas.Lastly we wash very carefuly and very well the peritoneal cavity with normal saline.If there is lithiasis of the gallbladder we go on and we do Cholecystectomy.If we find no stones in gallbladder we do Cholecystostomy.On coexistance of jaundice we on with the exploration of Choledochus duct.At the end we drained the suphepatic region(Winslow Foramen) (Koussidis 1988).

Patients and methods:We have operated under this operative procedure eleven patients aged from 42-88 year old. Eight of them were women and three were men.All patients had massive necrotic pancreatic and peripancreatic lesions and ascites.

Results:Ten of the patients postoperative were in good health.They dismissed from the hospital on the seventh to towenty second postoperative day.The eleventh patient died on the fourth postoperative day,from massive pulmonary embolism.This patient was operated on the phase of anuria which continued after the operation.

Conclusion:Patients with acute necrotic pancreatitis can be cured with a single operation,if they go under the operated procedure which we have illustrated above and if they will be operated the right time.

References:

A.Koussidis.Acta Chirurgica Hellenica 1988:60:189

BT057 THE FORMATION OF A NEW COMMON BILE DUCT WHEN THE COMMON BILE DUCT IS NECROTIZED:FROM NECROTIC CHOLECYSTITIS-CHOLANGITIS OR IT IS OBSTRUCTED FROM SCLEROSIVE CHOLANGITIS.

A.Koussidis,M.Apsokardou.
Department of Surgery General Hospital of Chios.

The common bile duct may be necritized from necrotic Cholecystitis or Cholangitis.The common bile duct also may be obstructed from sclerosive Cholangitis.The above pathologies cause obstructive jaundice.The up today theraputic operative approch is the choledochojejunostomy as in Roux-en-Y.(Braasch and Rossi 1985).Althrough this operative procedure is been used,this may not be able,to be done all the times because the tissue of common bile duct and the tissue of jejunum are very inflammated and in sclerosive cholangitis the truck may lead to stricture formation.Because of the above we have succefully introduced a new operative approch.We have made up a new truck of common bile duct.

Technique proccedure:We first do a transduodenal sphincterotomy.Then we Follow up the common bile duct to the common hepatic duct.After this is done we cut the anterior wall of all common bile duct.Then in that cut we put a T tube(Kehr).Such as one side of it to be introduced in to the common hepatic duct and the other side is indroduced into the jejunum througt the ampulla of Vater. Then we close up the cut of jejunum and later we cover up the anterior side of T tube with epiploic tissue. (Meissner K,Meiser G.1987).After three months we remove the T tube.We have operated two patients under this operative treatment.One 36 years old woman who had necrotic Cholecystitis-Cholangitis and a man 74 years old who had obstruction from sclerosive Cholangitis.Both the woman four years later,she is doing well and the man a year later is very healthy and very active.

References:
Braasch J,Rossi R.Surgical Clin.of North America 1985: 65:274
Meissner K,Meiser G.Chirurg 1987:58:154

### BT058 OUTCOME OF PANCREATIC DUCTAL CARCINOMA

H.A. Laffaye, E.J. Tracey, J.M. Wilson and G. Reynoso. Yale University and Norwalk Hospital, Norwalk CT, USA

The purpose of the study was to determine the outcome of patients with ductal carcinoma of the pancreas.

One hundred and forty-one consecutive patients with histologically proven ductal carcinoma of the pancreas were studied. The period extended from January 1978 through December 1987. Follow-up was 99.3%.

The 65 men and 76 women had a median age of 70 years (range 41-93) at the time of diagnosis. One hundred twenty eight tumors were located in the head and 13 in the body and tail.

Forty-two patients with a median age of 69 years had no surgery. The longest survivor lived 11 months, the median being 2 months. Eight patients (median age 77) were treated with biliary stents. Median survival was 2 months and the longest 13 months.

Seventy-eight patients (55.3%) underwent a bypass. Their median age was 71 and their median survival 5 months; the longest being 40 months. Three patients are alive at 40, 16 and 13 months.

Thirteen patients (9.2%) with a median age of 63, underwent resection. One patient is alive with disease 17 months after surgery. Median survival was 12 months; the longest being 40 months.

There were no 5-year survivors. One year survival for all patients was 21.9%; 2 years 5.6% and 3 years 3.5%. For those resected, survival was 61.5%, 23.1% and 15.4%, respectively.

The median age (63) for those undergoing resection was lower than the entire cohort (70). The 12-month median survival obtained for those resected was significantly longer than for those not resected, 2, 2, and 5 months ($p = 0.05$).

Pancreatic ductal carcinoma is a highly lethal clinical entity. Most patients have advanced disease at the time of diagnosis, with a resectability rate of approximately 10%. Resection, if performed with an acceptable mortality rate, appears to offer better palliation than either by-pass or stenting. The only pathological feature of apparent significance in the 5 patients surviving more than 3 years was the well-differentiated histology of the tumor.

**BT059** HISTOPATHOLOGICAL STUDY OF THE GALLBLADDER AFTER LIGATION OF THE HEPATIC ARTERY IN DOGS

LEKAKOS N., BLIOURAS N., TZARDIS P., RESTOS S., KEKIS B.

JANION GENERAL HOSPITAL-A.M.C. ATHENS,GR.

There is a coutroversy between investigators about routine cholocystectomy after hepatic artery ligation in man.

The purpose of this study is to evaluate the macroscopic and microscopic changes of the gallbladder, following hepatic artery ligation in dogs.

In 18 dogs under general anesthesia the arterial blood supply to the liver was obliterated by complete excision of the hepatic artery. The animals were reoperated between the 8th and the 20th postop day and the gallbladder removed and studied macroscopically and by histological examination.

The histological changes of the gallbladder wall in 15 out of 17 dogs, were those of acute cholocystitis.

We conclude that simultaneous cholocystectomy is not essential in dogs undergoing hepatic artery ligation.

BT060 ACUTE ALCOHOLIC NECRO-HEMORRHAGIC PANCREATITIS

L.S. Leonardi; F. Callejas Neto; J.C. Pareja; E.A. Chaim

Unicamp Medical School, Campinas-SP, Brazil

The authors studied 20 male patients submitted to surgery for acute alcoholic necro-hemorrhagic pancreatitis during the period 1980-1989. Their age ranged from 22 to 75 years, an average of 33 years.

Seven patients (35%) were operated during the early phase (first 4 days) with a mortality rate of 71.4%. No bacteriological growth was observed in the peripancreatic fluid

In the middle phase (4 to 15 days) six patients (30%) were operated for treatment of pancreatic necrotic tissue, with a mortality rate of 83%. Bacteriological growth in the peripancreatic fluid was observed in 83% of the cases.

In the late phase (after 15 days) seven patients (35%) were operated with a mortality rate of 42% and Bacteriological growth in the peripancreatic fluid was observed in 85% of the cases.

The conclusion arrived at by the authors was that the mortality rate in patients submitted to surgery for acute alcoholic necro-hemorrhagic pancreatitis is very high, with an overall mortality rate of 65%, and that these appears to be no relationship between the mortality rate and infection of necrotic pancreatic and peripancreatic tissue, especially in the early phase.

The study also concludes that surgery in the late phase presents the lowest mortality rates, in spite of the higher incidence of infection of necrotic pancreatic and peripancreatic tissue.

### BT061 PERITONEOVENOUS SHUNT IN THE MANAGEMENT OF ASCITES AND ASSOCIATED COAGULOPATHY

G. Lesti, R. Massari, M.C. Strusi,
A. Pierfelice, V. Beltrami
Istituto di Clinica Chirurgica,Chieti,I

41 patients, suffering from hepatic cirrhosis with massive aterile ascitic fluid not respondent to a dietetic and diuretic treatment, without recurring gastroesophageal haemorrage and advanced hepatorenal syndrome underwent the positioning of peritoneovenous shunt of Le Veen, under local anaesthesia. Before positioning the shunt, all the patients underwent 2 or 3 paracentesis with removal of about 80% of ascitic liquid which was replaced by physiological solution in the order of 50% of the amount of the fluid removed. A patients'thromboelastogram was always obtained and compared with another one performed after addition of ascitic liquid in the ratio 3:1 to the patients'blood; an abnormal variation of tromboelastogram width after addition of this liquid made necessary further diluitions of ascitic fluid, until the results were normal. Such technic standard was used in order to prevent an important complication of the peritoneovenous shunt that is the post shunt coagulopathy. This complication is liable to follow the infusion of ascitic liquid in the circulatory system. In fact recent studies showed that ascitic fluid contains a plasminogen activator factor which, in contact with the blood, causes a defibrination syndrome; this factor is inhibited by epsilonaminocaproic acid. The most common complication observed after the positioning of the shunt was its occlusion expecially at the venous extremity (8 cases) and at the valve (9 cases). Of the 41 patients, 12 have died: of which 3 during the immediate post-operative period (PSC, hepatic coma, oesophageal haemorrage) and the remaining 9 some months after the procedure (hepatic coma, oesophageal haemorrage, peritonitis).

BT062 MAGNETIC RESONANCE ANGIOGRAPHY: A NEW TECHNIQUE FOR ASSESSING SHUNT PATENCY

W D Lewis (1), R L Jenkins (1)
J P Finn (2), R R Edelman (3)
H E Longmaid (2), R A Kane (2)
Departments of Surgery (1) and Radiology (2), New England Deaconess Hospital and Department of Radiology, Beth Israel Hospital (3), Boston, MA 02215, USA

Current methods of imaging porto-systemic shunts have drawbacks. Duplex ultrasound (DUS) is effective in porto-caval shunts, but frequently fails to visualize spleno-renal anastomoses. CT scanning is unreliable and spin-echo MR imaging is complicated by respiratory and flow-induced phase effects. We compared the findings on sequential two-dimensional magnetic resonance angiography (MRA) to those on DUS in 2 patients with porto-caval and 3 patients with spleno-rental shunts. All patients were clinically stable. For MRA studies, single-slice flow-compensated images were acquired in multiple planes and these were post-processed to form 3-dimensional projection angiograms. Graphic information on flow direction was derived on MR by presaturation bolus tracking. MR studies were carried out on an IT imaging system and DUS examinations were performed on a computed sonography unit with color and pulsed Doppler capability. Patency and flow direction in the shunts was established in all 5 patients on MRA. The anastomoses were clearly visualized in all cases and free rotation of the 3-dimensional images optimized viewing angle. Both porto-caval shunts were visualized on DUS, but the spleno-renal anastomoses were obscured by bowel gas. We conclude that MRA is a robust and effective technique for assessment of shunt patency and flow directionality. For these purposes, it may prove to be the method of choice.

### BT063 EXPANDABLE METALLIC STENTS FOR POST-LIVER TRANSPLANT BILIARY STRICTURES

R.R. Lopez, C.W. Pinson, J. Pennings,
E.B. Keeffe, K. Ivancev
Oregon Health Sciences University
Portland, Oregon, USA

Both anastomotic and non-anastomotic biliary strictures following liver transplantation are frequently encountered. Surgical revision is well accepted for anastomotic strictures. These strictures can also be dilated with balloon catheters; however, restenosis is common. The use of permanent biliary endoprosthesis reported for benign and malignant biliary obstruction has not been reported following liver transplantation.

A patient who developed a choledochocholedochostomy anastomotic stricture following liver transplantation was balloon dilated at 1, 6, and 8 months post-operatively. The stricture recurred and the choledochocholedochostomy was converted to a choledocho-jejunostomy. Subsequently, the hepatic artery thrombosed. He presented to our institution 4 months later with hyperbili-rubinemia and cholangitis. Cholangiography revealed severe stenosis of the biliary-enteric anastomosis, proximal dilatation of the common hepatic duct which contained multiple stones, and segmental stenosis of the left hepatic duct. Using a percutaneous approach, the strictures were dilated and the stones manipulated into the intestine. Modified Gianturco self-expanding metallic stents were then placed across both the intrahepatic and anastomotic strictures. The patient became asymptomatic and hepatic enzymes and serum bilirubin returned to normal. Cholangiography 3 months later demonstrated wide patency of the previously stenotic segments, no stent migration, and no new strictures. The patient remains asymptomatic with normal laboratories and has had no complications at 6 months.

Modified Gianturco stents may provide long-term relief for post-transplant anastomotic and non-anastomotic biliary strictures and their pathologic sequelae.

### BT064 PARTIAL ARTERIALIZATION OF THE PORTAL VEIN ON THE DEARTERIALIZED LIVER

K. Maeda, K. Ueno, I. Miyazaki
The Second Department of Surgery, Kanazawa University School of Medicine, Kanazawa, Japan

We compared the influence of hepatic arterial obstruction on the hepatic circulation and tissue metabolism between animals with and without partial arterialization of the portal vein. Mature mongrel dogs were divided into four groups: a group in which the collaterals to the liver were obstructed and the hepatic artery was dissected (hepatic artery ligated group); two groups in which a bypass was grafted between the femoral artery and portal vein, and blood was sent by a Biopump at a rate of 100 or 200 ml/min(100ml/min and 200 ml/min portal arterialization groups); and a group in which laparotomy alone was performed (sham operation group).

The hepatic artery ligated group showed CO accumulation and and acidosis in hepatic venous blood, reduction of oxygen supply to 60-70% of normal level, increase of oxygen consumption and marked increase of enzyme escape from the liver.

In the portal arterialization groups, sufficient oxygenation of portal blood was noted, and the oxygen demand and supply and tissue metabolism were kept approximately normal.

The optimum flow rate for partial arterialization of the portal vein seemed to be about 100 ml/min (approximately equal to the flow rate through the hepatic artery). At the flow rate of 200 ml/min, the original portal blood flow was reduced, leading to portal hypertension and elevation in GOT and GPT.

These results indicate that ligation and dissection of the hepatic artery during obstruction of collaterals to the liver markedly affects the liver function, and that partial arterialization of the portal vein preserves the liver function during and in the early period after dissection of the hepatic artery.

### BT065 DETERMINATION OF PATIENTS OF HIGH RISK BY CT SCANNING IN ACUTE PANCREATITIS

W. Maier, M. Buechler, R. Roscher
Departments of Radiology and Surgery
of the University, Ulm, FRG

In 83 patients with proved acute pancreatitis the Computed Tomographic (CT) parameters of the disease (enhancement defects on dynamic CT, density and volume of peripancreatic infiltration) were correlated with clinical parameters (mortality rate, incidence of complications, duration of hospitalisation) and the Early Prognostic Signs of RANSON.
The outcome of the disease could be predicted very precisely using the CT parameters of acute pancreatitis. A CT score system was able to separate those patients with a severe clinical course from those with a uncomplicated one. Our CT score system and RANSON's system of prognostic signs showed a nearly linear correlation. Interestingly, the extrapancreatic CT parameters of acute pancreatitis were mor acurate in predicting the outcome than the intrapancreatic ones.
In summary, CT is capable at an early stage to differentiate between patients who run an uncomplicated course of acute pancreatitis and those who are of high risk.

BT066

### LIVER BIOPSY
### ITS DIAGNOSTIC EVALUATION IN LIVER IN JAUNDICE IN 100 CONSECUTIVE CASES

R Malik, R Gondal, T K Malik
Maulana Azad Medical College and
G B Pant Hospitals, New Delhi, India

Needle biopsy of the liver is done as a routine in the investigation of cases of cholestasis. With the introduction of the Menghini technique described by Morris et al, the procedure is considered fairly safe. At times it becomes extensively difficult to differentiate the medical cause of jaundice from the surgical cause on clinical grounds alone. In such situations liver biopsy may prove very helpful and obviate the need for ERCP or percutaneous cholangiography. Histomorphology of the liver in 100 consecutive cases of jaundice was studied. The parameters evaluated were lobular architecture, morphology of liver cells, Kupffer cells, degree and extent of cholestasis, severity and type of inflammatory infiltrate and changes in the portal triads.

In the communication, we report our experience of the diagnostic value of liver biopsy in differentiating medical from surgical cause of jaundice. We also give the spectrum of disease pattern encountered in distinguishing various forms of extra-hepatic biliary cholestatis.

References:
Morris J S et al (1975). Percutaneous liver biopsy in patients with large bile duct obstruction. Gastroenterology 68, 750-754.

BT067

### COMPARATIVE EVALUATION OF CHOLEDOCHODUODENOSTOMY AND TRANSDUODENAL SPHINCTEROPLASTY IN BILIARY DECOMPRESSION

T K Malik, P K Jain, P Bawa, R Malik
Department of Surgery, Maulana Azad
Medical College, New Delhi, India

Both choledochoduodenostomy (CDD) and transduodenal sphincteroplasty are valuable for choledocholithiasis and papillary stenosis (Rutledge 1976). In this study 50 patients were subjected to these procedures (25 patients in each group). Age ranged from 17 years to 70 years with female/male ratio of 2.5/1. Choledocholithiasis was encourntered in 80% of patients. Wound infection in two patients (8%) and residual stone in one patient (4%) were observed in TSP group. Residual stone was flushed by saline irrigation for one week through T-tube. In CDD group wound infection in two patients (8%) and prolonged biliary leak in one patient (4%) were seen. No other complications or mortality were observed in either group. Liver function tests at the end of one month were observed in all patients of both groups. Barium cholangiography showed free reflux with prompt emptying in all patients with CDD while in 80% of patients with TSP at the end of one month. All patients in TSP group showed free flow of dye, unaffected by I/V morphine on the 10th post-operative day. Follow up period ranged from four months to three years. In this series both the procedures were found to be equally safe and effective methods of drainage of benign biliary tract obstructions.

References:

Thomas C G, Nicholson C P, Owen J. Ann Surg. 173(6): 845, 1971

BT068 SURGERY DURING SEVERE FORMS OF CHRONIC PANCREATITIS

J.Manniste, M.Eivin, P.Alas, A.Maesalu
Republican Hospital, Tallinn, ESTONIA

A comprehensive cytolitic process of the exocrine and endocrine system, on the interstitial tissues of the pancreas induce the transformations of the ductal systems which causes some clinical syndroms, which need elective surgery in major cases. In 197 cases during the last 14 years the following methods were used in the sugery of the severe forms of chronic pancreatitis with clinical syndroms (dolorous, obstruction of the biliary duct or digestive canal, comprehensive destructions, arrosive bleeding or adenoms). Resection of pancreas in 73 cases (whipple operation - 11; distal resection - 17, among them 9 cases preserving the spleen; body resection - 2 with blombing the tail part of duct, and 2 with preserving of duodenum; pseudocysts, adenoms, paraganglioms et c. are resected in 41 cases). Pancreatodigestive anastomosis in different modifications was used in 34 cases, among them the $CO_2$-laser was used in 6 cases. Operations of neighbour organs with removing of pathological syndroms in 47 cases. Operations on the pancreas by secondary (biliary) pancreatitis were performed in 53 cases. Mortality rate in all groups was 5,5% (11 cases). We discovered the unfavourable criterion of prognosis allowing estimating the outcome for 5 years and farther. There are diabetes, diarrea, injury of liver, alcoholabuses, calcifications of gland tissue and malignancy. The surgery of chronic pancreatitis is considered as an emergency measure. During surgery it si necessary to by ready to perform radical operations, if during an operation a malignant tumour is discovered.

### BT069 SOME CRITERIA OF THE PROGNOSIS OF BILIARY PANCREATITIS

J.Manniste, H.Poola, M.Eivin,

A.Viiklepp, Tallinn.

An analysis of early and late results of surgical treatment of 230 patients with biliary pancreatitis in presented. Mechanical jaundice was observed in 90 patients (39.1 per cent). Surgery on biliary ducts has its risks and adverse side effects which determine prognostical criteria for survival and for certain complications. Euristic prognosis for survival in biliary pancreatitis is made on the basis of timely corrigation of pathological changes in the bile ducts and pancreas. Besides operations on the biliary tract, surgical interventions on the pancreas were carried out in 31 patients (13.5 per cent). Immediate postoperative fatality in the secondary pancreatitis with multilocated and comprehensive sequestration of pancreas was 9.1 per cent. Late complications, requiring repetitive surgical interventions (suppuration, recurrent sequestrations, arrosive bleedings, etc.) occurred in 7.3 per cent of cases.

Criteria for prognosis in biliary pancreatitis are as follows:

1 duration of jaundice period (hepatic lesions);

2 the presence of infection (bacteriobilia);

3 tissue regeneration disturbances during postoperative period (dysproteinaemia);

4 grave tissue lesions in the pancreas (diabetes).

### BT070 RESECTION OF HEPATOCELLULAR CARCINOMA IN CIRRHOTIC LIVER THROUGH A RIGHT THORACOTOMY

C.MARGARIT, J.BALSELLS, E.MURIO,
JL. LAZARO,R. CHARCQ,I DIAZ
Hospital G.Vall d'Hebron.Barcelona.Spain

We present a Video of a 74 years old male diagnosed of hepatocellular carcinoma by means of ultrasound examination, FNA cytology and elevated alphafetoprotein. He had been diagnosed of alcoholic liver cirrhosis 20 years before and during the last 2 years he suffered several episodes of ascites. We decided to use a thoracic approach because he presented several abdominal scars from previus laparotomies for cholecistectomy, bile duct exploration and apendectomy and the tumor was located in the dome of right lobe.

A right posterolateral thoracotomy through the sixth intercostal space was performed and the abdominal cavity was entered through the right diaphragm. Mobilization of the right lobe was done and exploration of the cirrhotic liver could not detect the tumor. A well limited hepatoma was localised in segments IV and VIII by means of the intraoperative ultrasound examination and resected under guidance of the US. The median hepatic vein was dissected and several branches ligated. Hemostasis was achieved with sutures, Tissucol and collagen. An aspirative drainage was placed under the diaphragm and another in the thorax. The postoperative course was uneventful and the patient is alive and well without evidence of tumor recurrence nine months after the operation.

The pathology report showed a well differenciated hepatocellular carcinoma without vascular invasion and safe surgical margins. Moderated active liver cirrhosis.

BT071

### DISTAL SPLENO-RENAL SHUNT (WARREN'S SHUNT)

C.MARGARIT, R. CHARCO, JL. LAZARO, E. MURIO
J.BALSELLS, I DIAZ
Hospital G. Vall d'Hebron.Barcelona Spain

We present a Video of a 67 years old male diagnosed 10 years earlier of liver cirrhosis during a cholecystectomy. He presented two episodes of esophageal variceal bleeding treated by sclerotherapy. An elective spleno-renal shunt operation was indicated. A subcostal bilateral incision was performed. Adhesions from previous operation were taken down. The splenic vein was approached through the transverse mesocolon and dissected from the pancreas by ligating several pancreatic branches. The retroperitoneum was entered and the left renal vein was dissected. A termino-lateral spleno-renal shunt was fashioned with a double running Prolene suture. The operation was completed with a disconnection from the portal territory by ligating the gastroepiploic vessels, coronary vein and section of the great omentum. The postoperative course was uneventful and the patient is alive and well 10 months after the operation.

**BT072** RIGHT HEPATIC TRISEGMENTECTOMY FOR METASTASIC NEUROBLASTOMA

C.MARGARIT,JL.LAZARO,R.CHARCO,J.BALSELLS
E.MURIO,I.DIAZ,C.CORTES
Hospital G.Vall d'Hebron.Barcelona.Spain

We present a Video of a 21 years old male inderwent a laparotomy for abdominal tumor in april 1988.A huge intraperitoneal tumor sticked to the great amentum was found and resected.At the same time a tumor in the right lobe of the liver was found.Pathology report was undifferenciated sarcoma.The patient was treated with chemotherapy during 9 months with partial response at the begining.In march 1989 an increase in the size of the liver metastases was observed in spite of the chemotherapy.Other metastases outside the liver were ruled out by means of thoracic,cranial and abdominal CT scans. A surgical resection of the liver metastases was proposed and accepted by the patient.

A right subcostal incision with a midline extension was performed.A huge tumor occupying the right lobe and segment IV of the liver was found,a small nodule was present also inthe inferior face of the left lateral segment. No other evidence of tumor was found in the abdominal cavity.First to all the hepatic hilum was dissected, ligating the right hepatic artery, portal vein and bile duct.A firm tumor adhesion to the right diaphragm obliged to perform a right thoracotomy and to resect part of the diaphragm.Then, the right hepatic vein as well as other accesory hepatic veins were dissected and ligated.A right hepatic trisegmentectomy was performed, transecting the liver perenchima one cm. to the right of the falciform ligament.The hemostasis was achieved with sutures, electrocautery and Tissucol. Then we turned attention to the 3 cm. nodule localted in the left lateral segment that was resected. Finally the gastroduodenal artery was dissected and a catheter was placed connected to a subcutaneous reservoir for later regional chemotherapy.

The specimen showed a huge necrotic tumor occupying all the right hepatic lobe with a peripheral nodule in segment IV.

Teh pathology report: indifferenciated amlignant tumor of possible neuroectodermalorigin ,consistent with neuroblastoma.

BT073

### ACUTE NECROTIZING PANCREATITIS - REPEATED ABDOMINAL LAVAGE IN THE INTENSIVE CARE UNIT ULTILISING A MERSILENE MESH AND A ZIP

N.I. Markham
Department of Surgery,
Prince of Wales Hospital,
Chinese University of Hong Kong

The management of acute necrotizing pancreatitis is difficult and whilst the basics of supportive care are not controversial, the techniques of managing the septic abdomen are the subject of much debate. We wanted to assess the feasibility and results of daily peritoneal lavage, performed on the Intensive Care Unit (ITU), with access to the abdominal cavity through a mesh and zip.

We have now treated 8 patients with acute necrotizing pancreatitis in this way. All presented with severe attacks necessitating laparotomy, at which time gross infection, inflammatory exudate or necrotic debris were removed from the peritoneal cavity. No attempt was made to close the abdomen - a mersilene mesh incorporating a standard domestic non-metal zip was sewn to the skin and the patient returned to the ITU. No drains were placed. Daily laparotomies were performed thereafter, unzipping the mesh, allowing a thorough lavage using 2-3 litres of warm saline. General anaesthesia was not required, the patients usually only needing intravenous sedation and analgesia. The procedure was repeated until the peritoneal cavity was considered clean and the mesh and zip were then removed about 5 days later. Wounds did not require suturing but closed by a combination of granulation and scarring.

Two patients died of multi-system failure and a further succumbed to an uncontrolled haemorrhage from an erosion into the inferior vena cava. The remaining 5 patients all eventually made a full recovery.

We found the procedures very convenient and they were well tolerated by the patients who were easier to manage as a daily check on the intra-abdominal infection status could be made. Most would certainly have required further formal laparotomies had this technique not been adopted, so thus we saw none of the traditional problems of continually resutured wounds such as tension, necrosis or dehiscence.

We believe that with careful patient selection this particular technique has much to commend it - it is safe, well tolerated and provides a daily convenient means of assessment and an opportunity for removal of infective material as it accumulates.

**BT074** ***PALLIATIVE PROCEDURES FOR BILE DUCT CANCER***

*L.A.Marzano, * R.Lobello, A.Porcelli, H.Cocucci,M.d'Ajello and L.Zarrilli.*
*Univ.Napoli,II Fac., Endocrinochirurgia e * Serv.Spec.Fis.App.Dig.*

*In the last 10 yrs. 28 patients, 18 men and 10 women, ranging in age from 35 to 78 yrs. (mean 57 yrs) received palliative procedures: i.e., biliary enteric bypass (18 patients), percutaneous biliary prothesis (6 patients) and endoscopic retrograde drainage (4 patients). The preoperative assessment was made by U.S. and TC scan, E.R.C.P. and celiac and superior mesenteric arteriography. The sites of the hilar tumours had been shown preoperatively to correspond to Bismuth and Corlette's (1975) type II in 14 cases, type III in 8 cases, and type IV in 6 cases. In our group, 18 patients were undertaken for bypass surgery, and successfull biliary decompression was achieved in 80 % . The post-operative complications were cholangitis (1 patient) and temporary bile leak (3 patients) with a postoperative morbidity of 22.2 %. The survival rate was 11.5 months after surgery. Only 1 patient died in the first 2 months post operatively (operative mortality rate 5.5 %). No support by a transanastomotic drain was used. The rest of the patients were treated by percutaneous transhepatic endoprosthesis (6 patients) or endoscopic retrograde drainage (4 patients). In the first group the relief of obstructive jaundice was achieved in all patients and the mean survival was 5.8 months. Postoperative morbidity was 50 % (3 patients). In the last four patients, endoscopic intubation was utilized. No morbidity rate was taken, but the mean of survival time, decreased to 3.4 months.Therefore, compared to percutaneous transhepatic prosthesis and endoscopic retrograde drainage, surgical bypass offers a long lasting relief of jaundice, with a low mortality rate, less post-operative complication, and a better survival rate.*

BT075 **EXTENDED RADICAL OPERATION FOR CANCER OF THE BILIARY TRACT OBSTRUCTING THE HEPATIC HILUS**

T. Matsumoto, T. Imabori, H. Hasegawa,
G. Kaburaki, T. Nagakawa, I. Konishi, K. Ueno,
T. Ota, Republic Ishikawa Matto Central
Hospital. Kanazawa Univ, Matto, Japan.

Cancer of the bile duct and gall bladder that obstructs the hepatic hilus is often invasive, and its radical operation is generally very difficult. At our institution, laparotomy was performed in 13 patients with cancer of the biliary tract obstructing the hepatic hilus namely 10 with bile duct cancer and 3 with gall bladder cancer, between April, 1983 and December, 1989. Resection of the lesion was possible in 7 patients with a resection rate of 53.8%. The surgical procedure was extended left lobectomy in 2, hilar hepatectomy in 2, pancreatoduodenectomy in 1 with middle bile duct cancer invading the upper bile duct, and extended cholecystectomy with pancreatoduodenectomy in 2 with gall bladder cancer invading the hepatic hilus.

Concerning the remote results of the resected 7 patients, five has survived from 5 years and 4 months, to 5 months, but two died 11 months or one month after operation. Histopathological findings of lymph nodes were negative for metastasis in all patients with cancer of the upper bile duct or the hepatic duct, but metastases to mesoduodenal lymph nodes, lymph nodes posterior to the head of the pancreas, and those around the common hepatic artery were observed in some patients with middle to lower bile duct cancer or gall bladder cancer.

On the basis of these results, we currently perform the active radical operation. In this study, we will present clinicopathologically the choice of operative procedures on cancer of the biliary tract obstructing the hepatic hilus.

BT076 BILIARY TREE CHANGES IN SCHISTOSOMAL HEPATIC FIBROSIS STUDY USING DIRECT ENDOSCOPIC RETROGRADE CHOLANGIOGRAPHY

F. Mekky, O. Shafey, A. Abdel-Moeti, M. Rashed, A. Ghanem. Faculty of Medicine, Alexandria University, Egypt

This study of 21 patients with schistosomal hepatic fibrosis revealed the following: Flatulent dyspepsia was a distressing symptom in 95%. Ultrasonography and laboratory tests showed no characteristic patterns. Gall stones were detected in 4.8%, dilated gall bladder in 38% and it was thick walled and contained biliary mud in 28.6%. Histologic study of liver biopsies demonstrated: bile duct proliferation 62%, cirrhotic changes: 52%, histologic cholestasis: 47.6%, bile duct epithelial pseudostratification: 14.3%, excessive periductal fibrosis: 14.3%, lymphoid follicles: 9.5%, bile duct destruction and invasion with macrophages: 4.8%. Endoscopic retrograde cholangiography showed three characteristic patterns: 1) Filling of normal extrahepatic ducts with minimal intrahepatic filling in 19%. 2) Filling of normal extrahepatic ducts with intrahepatic attenuation and less branching up to pruning in 28.6%. 3) Filling of attenuated intrahepatic duct with irregular walls and less branching in 52.4%.

BT077 OPERATIVE CHOLEDOCHOSCOPY

R.K. Tompkins, O. Shafey, F. Mekky,
H. Hassab. Alexandria University
Hospital, Egypt

The advent of the choledochoscope allowed closer examination of the bile duct with better diagnostic and therapeutic yield of CBD exploration. Operative choledochoscopy integrates with traditional CBD exploration adding to its accuracy and reducing the rate of retained stones and overlooked soft tissue lesions in the bile ducts. We performed this procedure in 18 patients in whom CBD explortion was indicated, 11 with bile duct obstruction, 2 for bile duct stones, 3 for pancreatic stones and 2 for ampullary stenosis. We had positive findings in all cases; 7 patients had adenocarcinoma of the extrahepatic bile ducts and biopsies were taken under vision, 2 had benign papillary tumours in the hepatic ducts and 1 had fibrosing pancreatitis. 3 patients had bile duct stones and 3 had pancreatic stones. The 2 patients with papillary stenosis, one had patulous CBD with sludge and the other had benign shaggy frondy material removed successfully.

There was one failure in a case of missed stone (5.5%) but no morbidity or mortality was recorded.

Choledoscopy proved to be useful in visualization of lesions, observing their nature and extent, providing adequate biopsy material. We found that it should be used whenever CBD exploration is indicated.

### BT078 HAZARDS OF CHOLECYSTECTOMY IN PATIENTS WITH SCHISTOSOMAL HEPATIC FIBROSIS; MEDICOSURGICAL ASPECTS

F. Mekky, A. Abdel-Moety, M. Rasheed, D.M. Baymoumi, M.Y.T. Rashed, A.M F. Ghanem. Faculty of Medicine, Alexandria University, Egypt

Fifteen patients with chronic calcular cholecystitis associated with schistosomal hepatic fibrosis have been studied regarding the intra-operative difficulties and hazards, as well as the immediate postoperative complications. Eight males and seven females with a mean age of 39.7 years were included. Thorough clinical examination as well as laboratory investigations - liver profile and bleeding and coagulation times were done. Real time ultrasonography was done for all patients. Removed gall bladders were submitted to postoperative histopathological examination. The commonest operative and postoperative hazard was bleeding due to dissection of adhesions around the gall bladder, especially its bed; this was encountered in 60% of patients. Mean volume of blood loss was 718 ml in eight patients with advanced periportal fibrosis, while it was 250 ml in seven patients with mild degree of fibrosis. The commonest immediate postoperative hazard was also bleeding from the gall bladder bed found in 60%. Deep wound infection occurred in 13.3% and ascites accumulated in 6.62% of the patients. Cholecystectomy in patients with hepatic schistosomiasis is not without hazards.

BT079 PERCUTANEOUS CHOLEDOCHOSCOPIC REMOVAL OF RETAINED COMMON BILE DUCT STONES VIA THE T-TUBE TRACT

D.Menzies and R.W.Motson, Colchester General Hospital, Colchester, UK.

After exploration of the common bile duct the incidence of retained stones found on T-tube cholangiography may be as high as 10%. Accepted methods of removal of such stones include endoscopic sphincterotomy and Dormia basket extraction under fluoroscopic control (Burhenne technique). Both methods require some degree of expertise and may not be freely available in all hospitals. We present a simple method of stone extraction under direct vision that may be performed by general surgeons under simple sedation.

Four patients were referred with retained stones on T-tube cholangiography. The T-tube was left in-situ for six weeks without morbidity. Under intravenous sedation a flexible choledochoscope was inserted down the T-tube tract. Stones were removed under direct vision with a Dormia basket. Common bile duct clearance was achieved in each case in 1 - 4 sittings and confirmed with tube cholangiography the next day. Between procedures the tract patency was maintained by insertion of a Foley catheter. No complication from the procedures occurred.

Percutaneous choledochoscopic removal of retained common bile duct stones is an acceptable alternative to recognised methods of stone removal.

**BT080** SURGICAL PROCEDURES OF BENIGN BILIARY STRICTURES. LONGTERM RESULTS .

M. Gioffrè, G. Giacobbe, A. Cogliandolo, B. Bonfiglio
B. Micali
General Surgery, University of Messina, Italy

The surgical treatment of benign biliary strictures (BBS) would seem still to play the most satisfactory role,even if the advent of non-operative techniques- e.g. percutaneous transhepatic balloon dilatation- has yielded early good results in only selected cases (1). Operative repair of BBS,however,remains a difficult problem considering the controversies in the long term results (2).
This study was undertaken to identify the most suitable surgical technique of bile duct strictures.

Materials and Methods. Fifteen patients with BBS (8 males and 7 females,ranging in age 37 to 78 years) treated between 1978 and 1987 were reviewed in this report. The stricture etiology was gallbladder inflammation by cholelithiasis (N°5),II type of Mirizzi syndrome (N°4),chronic pancreatitis (N°8),iatrogenic injuries (N°3).Surgical procedures are reported in the table below.Longterm results (mean follow-up 5 years) were assessed by clinical, laboratory and imaging studies.

| | | | |
|---|---|---|---|
| Hepaticojejunostomy | N° 6 | Choledochoduodenostomy | N° 6 |
| Cholecystectomy + T tube | N° 2 | Cholecystectomy + cystic drain | N° 1 |

Results. Operative morbidity was 13.3%.There were no operative deaths.Successful results were recorded in 11 cases (73.4%),Fair results in 3 (20%),poor in 1 (6.6%). Failure was observed in one patient undergone choledochoduodenostomy.Fair results included choledochoduodenostomy (N°3).

Conclusions.The surgical approach of BBS would appear to be the first choice treatment in order to carefully manage strictures and associated disorders as jaundice and inflammation.Several surgical procedures can be carried out,however the most accurate technique to avoid unpleasant consequences of a later stricture is required. From this point of view Roux-en-Y biliary enteric diversion has been the most successful in our series with an overall success rate of 100%.Choledochoduodenostomy is widely applicable,however it has yielded longterm complications.The beneficial use of T-tube stent is controversial,however when it was left in thin-walled bile duct excellent results were recorded.

References

1) Gallacher D.J. et al Radiology 156:625-9, 1985.

2) Genest D.F. et al Surgery 99:409-13, 1986.

**BT081** DOUBLE SYNCHRONOUS LIVER AND KIDNEY TRANSPLANTATION

E. MORENO; I. GARCIA; I. GONZALEZ-P; R. GOMEZ; C. LOINAZ; P. VORWARD; H. BONET
Hospital 12 de Octubre. Madrid.Spain

A patient suffering during four year chronic renal failure treated by haemodialysis was operated by a kidney transplant. Severe rejection occurred the graft removed and haemodialysis continue. Seven and a half years later postnecrotic liver cirrhosis and liver failure was diagnosed. A Liver & Kidney transplantation from a single donor was performed. The patient has a good renal and liver function three years later. This film shows the two operations step by step and the fail result.

### BT082 TOTAL CYSTECTOMY AND BILIARY-DUODENAL INTERPOSITION OF A JEJUNAL LOOP IN CONGENITAL CHOLEDOCHAL CYST.

E. MORENO; M. HIDALGO; I. GARCIA; A. CALLE;

J. IBAÑEZ; P. VORWALD; H. BONET

Hospital 12 de Octubre. Madrid. Spain

A two years old child, suffering from cholangitis, jaudice and digestive disturbances was diagnosed of choledochal cyst, was treated by total removal of the cyst (one block) and one isolated jejunal loop was used in the reconstruction, interposed between the hepatic ducts confluence and the duodenum.

### BT083 ASSOCIATION OF COMMON BILE DUCT STONES AND SILK LIGATURES IN BILIARY SURGERY

A. Mystakidis, R. Lygeraki, A. Lainas,
G. Velmahos.
Sotiria General Hospital, Athens, Greece.

Although it has been well known that non-absorbable suture material, used for ligation of the cystic duct, may find its way into the bile ducts and act as a nidus for stone formation, surgeons are still using such ligatures. This can occasionally result in serious postoperative problems that one could very easily avoid.

Purpose of this paper is to present three additional cases, where silk sutures used at cholecystectomy resulted in common bile duct stone formation.

On the basis of the above observations the use of absorbable sutures is strongly recommended for cystic duct and cystic artery ligation (Mackie D.B. et al 1973, Larmi T. and Silvennoinen E. 1968). In our Department Polyglactin sutures are used for ligations in the vicinity of bile ducts, although it is known that even absorbable sutures may form a nidus for subsequent common duct stones (Fink D.L., Budd D.C. 1983). Recurrent stones should always be examined for the possible presence of non-absorbable sutures in them.

References:

Mackie D.B., Haynes S., May R.E. Brit J Surg 1973; 60:23-4.
Larmi T., Silvennoinen E. Acta Chir Scand 1968; 134:82-4.
Fink D.L., Budd D.C. Int Surg 1983; 68:151-2.

**BT084** CLINICOPATHOLOGICAL EVALUATION OF LONG-TERM SURVIVORS TREATED FOR CANCER OF THE PANCREATIC HEAD

T.Nagakawa, K.Ueno, T.Ohta,
H.Kobayashi, Y.Nakano, T.Nakamura,
M.Kadoya, M.Kayahara, I.Konishi,
T.Matsumoto, I.Miyazaki, Kanazawa
University, Kanazawa, Japan

At our department, we have gradually extended the area of dissection in patients with pancreatic cancer since the end of 1973. With these efforts 8 patients survived for 3 years or more, and the 5-year survival rates in patients with cancer of the pancreatic head who tolerated macroscopic curative resection became 36.5%. In this study, the clinical data on these 8 patients were analyzed.

Based on the results of evaluation in our patients, the preconditions at present of long survival in patients with cancer of the pancreatic head seem to be 1) so(negative serosal invasion), 2) rpo(negative invasion to the retroperitoneal tissues) or ew(-)(negative invasion in excisional wedge) even if rpe(exposed invasion to the retroperitoneal tissues), 3) no lymph node metastasis or metastasis limited to the $n_1$ group. Especially, though 4 of the 8 patients (50%) had rpe, no cancer invasion was observed in the exposed peripancreatic surfaces, demonstrating histologically curative resection.

This seems to have achieved by extended operation that attaches importance to extensive retroperitoneal dissection and resection of the nerve plexus of the pancreatic head.

References:

Nagakawa T, et al. Jap. J. Surg. 1989; 19: 5.

Nagakawa T, et al. New Trends in Gastroenterology 1987; 205.

Nagakawa T, et al. Jap. J. Surg. 1982; 12: 3.

BT085 ROLE OF FREE RADICALS IN MOUSE ACUTE PANCREATITIS

– A noble free radical scavenger, CV-3611, attenuate the motality –

A. Nonaka, T. Manabe, T. Kyogoku,
K. Tamura, T. Tobe
Kyoto University, Kyoto, Japan

The therapeutic effects of a new synthetic scavenger made from an ascorbic acid derivative, CV-3611 (AAD) on a CDE-diet induced acute pancreatitis in mice were evaluated and compared with those of superoxide dismutase (SOD). The survival rate was observed in three groups; No treatment (N), Pretreatment (P), and Treatment (T) by the ascorbic acid derivative and by SOD.

The changes of three serum enzyme levels (amylase, lipase, elastase-I) were also measured in three groups by ascorbic acid derivative administration. Changes of plasma and pancreatic tissue concentration of AAD in normal mice were also measured by high performance liquid chromatography (HPLC) following the time course. Plasma concentration of AAD reached a peak level of 0.54 ± 0.09 $\mu$ g/ml at 1 hour and gradually decreased in 10 hours after subcutaneous administration. Pancreatic tissue concentration of AAD reached a maximum level of 425 ± 33 ng/g · tissue at 3 hours and returned to near the non-detectable zone in 12 hours. The survival rate was significantly increased in the Treatment group ($P<0.02$) by the administration of AAD. The administration of SOD had no significant effect on the survival rate. The increases of three serum enzymes, amylase, lipase, and elastase-I, were significantly reduced at 48 hours in both the Pretreatment and the Treatment groups with AAD. These results indicate that a new synthetic scavenger (AAD), which has been proven to pass through the cell membrane and to have a long half life in plasma and tissue, revealed a remarkable therapeutic effect on the development of acute pancreatitis. These results also suggest that oxygen derived free radicals might play an important role in the development of acute pancreatitis.

**BT086** *A RETROSPECTIVE STUDY OF 36 PATIENTS WITH CHOLANGITIS TREATED AT THE UH,KL*

*S.M. Noori, University Hospital, Kuala Lumpur, Malaysia.*

*This is a retrospective study of 36 out of 73 cases of cholangitis seen at the University Hospital, Kuala Lumpur, between 1980 - 1986. 21 were male and 15 female. The majority were above 40 years of age (17 above 60 years, 12 above 40 years) and most were from the low socio-economic group.*

*In 25 patients, jaundice, pain in right upper abdomen and chill were the chief complaints, 16 giving history of previous biliary surgery. The majority were seen within the first week of onset of the disease.*

*On admission, most patients were generally satisfactory but were found to be jaundiced with abdominal tenderness and fever of between 37 - 38°C. The liver was tender and palpable in 15 patients.*

*Investigations revealed raised globulins in 22 patients, alkaline phosphatase in 32, SGPT in 20 and SGOT in 20. The prothrombin times were normal in 26 cases and 6 cases showed impaired renal function. Blood culture was positie in 12 cases and bile culture in 16 cases.*

*The most useful diagnostic procedure used was ultrasonography, done on 29 patients with positive results in 20 cases. P.T.C. gave the diagnosis in 10 out of 11 patients. E.R.C.P. was useful in 3 out of 10 cases.*

*Nineteen patients underwent surgery, 12 treated conservatively, 5 refused operation and further hospital treatment. 13 were operated on emergency bases and 6 electiely. Cholecystectomy was done on 10 patients, C.B.D. exploration in 17 cases and T-tube insertion in 14 cases. Stones were found in 13 cases. 2 patients had liver malignancies.*

*Six patients developed wound infection, 4 had residual stone, 3 developed renal failure, 1 had D.I.V.C. and 1 acute pancreatitis. There was 1 death.*

### BT087 SONOGRAPHY OR CHOLANGIOGRAPHY IN DIAGNOSIS OF BILIARY TREE STONES

A. Noussias, D. Papadimitriou,
A. Tsironis. Municipal Hospital,
Athens, Greece

Ultrasonography is a bloodless and reliable procedure for the checking of cholelithiasis and jaundice, but its value declines in the presence of gas in the duodenum or the localization of stones in the distal part of the choledochal duct (Goldberg 1974). Intravenous cholangiography provides an excellent diagnostic method for imaging the biliary tree anatomy, the presence of stones and any morphologic or functional disturbance caused by them. Limitation of the method is the presence of jaundice (Mujahed 1974, Eckelberg 1970). ERCP is indicated on jaundice or suspicion of pancreatic disease.

During a four year period (1986-1989) 800 patients were operated on for calculi of the biliary tree. The pre-operative diagnosis was established with cholangiography and ultrasound imaging. The purpose of our study was to compare the diagnostic value of these two procedures in reference to the operative findings.

We concluded that the combination of these two methods, when it is possible to be performed, establishes the correct pre-operative diagnosis in a high rate (99%). Cholangiography seems to be more effective with a precision 97%. Ultrasonography gave false positive and false negative results in a rate 6.7%.

References

Goldberg B, Harris K & Brooker W: Radiology, 111,405-409,1974.
Mujahed Z: Radiology, 112:297-298,1974.
Eckelberg M D, Carlson H C & McIlrath D C: A.J.R, 110,235-239,1970

**BT088** A STUDY OF FLUOROURACIL, EPIRUBICIN AND MITOMYCIN-C COMBINATION CHEMOTHERAPY IN PATIENTS WITH UNRESECTABLE MEASURABLE PANCREATIC ADENOCARCINOMA.

C. Gennatas, D. Panoussopoulos, D. Dandoubas, J. Kouvaris.
University of Athens, Athens, Greece

Thirty-two patients with advanced measurable pancreatic adenocarcinoma were treated with a combination chemotherapy regime. Median age was 58 years. Twenty-three patients were male and 9 female. None had previous chemotherapy or radiation therapy. Treatment included Fluorouracil 600mg/$m^2$ I.V. days 1,8,29 and 36. Epirubicin 40 mg/$m^2$ I.V. day 1 and 29 and Mitomycin-c 10 mc/$m^2$ I.V. day 1. Cycles were repeated every 8 weeks. Partial responses have been noted in 7 patients (21,8%). Median survival from diagnosis was 8.2 months for responders and 5.5 months for non-responders. Toxicity included diarrhea (12%), myelosuppression (45%) and alopecia (82%). Pancreatic adenocarcinoma responds poorly to standard chemotherapy. New, active agents are definitely needed.

<u>References</u>:

Robustelli Della Cuna G, Genari L, Pellegrini A. Cancer of the digestive tract. In: Bonadonna G- Robustelli Della Cuna G (eds). Handbood of Medical Oncology, Masson, Milano, 1988.

Wils J, Bleibens H, Blijham G et al. Eur. J. Cancer. Clin. Oncol. 1985; 21:191.

BT089

### GALL STONE ILEUS

D. Papadimitriou, G. Gennadios,
A. Noussias, A. Tsironis
Municipal Hospital, Athens, Greece

Although an infrequent disease, gall stone ileus accounts for a significant percentage of bowel obstruction in elderly women, who also often had co-existant medical problems (Day & Marks 1975; Deitz 1986).

Plain films showing air in the bile ducts and a stone changing position may help in the difficult and unclear clinical diagnosis (Ringler 1941). The proper treatment is enterolithotomy alone (Kvist 1979) or the one stage procedure (Van Lindingham 1982).

Between 1978 and 1989 we accepted 11 cases of gall stone ileus. 72% were women and 90% more than 70 years of age. In 72% the stone was visualized and in 45% air was detected in the bile ducts; 62% underwent enterolithotomy alone and 38% one stage procedure. We have no ileus recurrence but 18.2% wound infections. The mortality rate was 9.9%.

Our findings suggest that the one stage procedure is preferred when the general condition and the local findings allow.

References

Day E A, Marks C: Am J Surg 1975:129:552.
Deitz M D et al: Am J Surg 1986:151:572.
Ringler L G et al: JAMA 1941:117:1753.
Kvist E:Acta Chir Scand 1979:145:101.
Van Lindingham S B, Broders C W: Surg Cl N Am:1982:62:241.

### BT090 CHOLEDOCHODUODENOSTOMY AS ROUTINE PROCEDURE FOR BENIGN BILIARY OBSTRUCTION

L. Papastamatiou, L. Lapidakis, J. Verigos
G. Kalantzis. Department of Surgery,
Apostle Paul Hospital, Athens, Greece

Although endoscopic papillotomy, transduodenal papilloplasty and several types of bilio-enteric anastomoses are today in use for benign biliary obstruction, early and especially late results of these procedures reveal a wide spectrum of complications.

Side to side choledochoduodenostomy (CDS) is well documented as a procedure of choice in elderly patients, despite old objections related to the myth of ascending cholangiitis and the sump syndrome. the fact that lege artis constructed CDS (2,5 cm stoma, absorbable one layer suture) results no early or late complications, led to the use of this type of biliary bypass in young patients and independently of their age.

Twenty-eight patients under the age of 60 (18-57) underwent CDS from January 1986-December 1989. Surgical indications were: Cholecysto+choledocholithiasis, thick walled common bile duct, narrowed distal choledochus and/or ampullary stenosis in 19 patients. Recurrent secondary or primary choledocholithiasis in 4 patients. Intrahepatic rupture of hydatid cysts in 2 patients. Ampullary stricture following endoscopic sphincterotomy in 2 and transduodenal papilloplasty in 1 patient. There was no mortality observed. An anastomotic leakage dried up within 4 days. All patients were free of abdominal symptoms, cholangiitis, chronic antral gastritis or pancreatitis during follow-up period (6-42 months). X-ray examinations one year postoperatively showed a satisfactory retrograde duodeno-choledochal passage in 22 patients. No stricture of the anastomosis was observed.

It is concluded that the lege artis choledochoduodenostomy is a safe and relatively easy procedure with long term satisfactory function, with no mortality, low morbidity rates and prevention of choledochal obliteration.

**BT091** NECROTIZING PANCREATITIS: TO IRRIGATE OR NOT THE PERITONEAL CAVITY AFTER NECROSECTOMY

S.Papavramidis, G. Goutzamanis,
I. Vogiatzis, O. Gamvros,
A. Aidonopoulos
Dept. of Surgery III, AHEPA Hospital
Thessaloniki, Greece.

Between January 1981 and December 1989, 22 patients required surgery for acute necrotizing pancreatitis. Eighteen of them showed extended intra and extra pancreatic necrosis with severe peritoneal involvement and the remaining 4 had a limited pancreatic necrosis only. Necrosectomy, cholocystostomy or choledochostomy, wide communication between lesser sac and abdominal cavity as well as intraoperative peritoneal lavage was followed by Postoperative Peritoneal Lavage (PPL) with Ringer's solution via 4 draining Argyle(R) No.32 tubes in 18 patients who developed extended necrosis. The total amount of the instilled solution ranged from 36 to 45 liters per patient and the duration of PPL was 3-4 days after operation. The criteria for application of PPL were pancreatic necrosis over 30% and diffuse peritoneal involvement of the disease in combination with shock and simple or multiple organ failure. The median Ranson's criteria score was 6.4 points for the patients who underwent PPL, and 4.5 points for the remaining. Operation was required at a median of 9.3 days after the onset of symptoms because of non-response to conservative treatment or because of acute abdomen formation. Nine of the patients developed hypovolemic or septic shock and 15 had simple or multiple organ failure. Four of the patients (18.2%) died of noncontrolled multiple organ failure dispite of surgical interventions, intensive supportive therapy and PPL. Necrosectomy is a suitable procedure for all patients with necrotizing pancreatitis. Furthermore in those who developed extended pancreatic and extrapancreatic necrosis with diffuse peritoneal development PPL seems to be a valuable contribution for their survival.

**BT092** PALLIATIVE SURGICAL TREATMENT OF PANCREATIC CANCER

C. Sperti, C. Pasquali, C. Militello
B. Bonadimani, F. Cappellazzo,
S. Pedrazzoli,
Clinica Chirurgica 1-Padua, Italy

Less than 20% of patients with pancreatic carcinoma may have a curative resection. Patients with unresectable cancer of the head of the pancreas nearly always need a palliative biliary by-pass. The role of a routinly performed prophilactic gastroenterostomy at time of initial surgical approach is still disputing.
We reviewed our experience of palliative procedures for pancreatic carcinoma in order to evaluate early and late results and the usefulness of gastroenterostomy in such patients.
From 1964 to 1988, 208 patients underwent palliative surgery for cancer of the head (157) and body and the tail (51) of the pancreas. TNM staging of the tumor was II in 31 patients, III in 36, IV in 141 (67.8 %). The most frequent symptoms were jaundice (154), pain (40) and gastric outlet obstruction (14).
154 patients (74 %) underwent biliary by-pass (28 cholecysto-enterostomies and 126 choledoco-enterostomies), associated with gastro enterostomy in 47 cases. 26 had external biliary drainage, 20 had gastro-enterostomy only and 8 other type of procedures. Overall operative morbidity and mortality were 37.5% and 18.7%.
In the group of biliary by-pass, morbidity and mortality rates were 37.3% and 15%. 12 patients required a second operation for gastric outlet obstruction, 7 in the early postoperative period and 5 in the late period (range 3-9 months) with 3 operative deaths. Median survival time was 2 months in patients who underwent cholecysto-enterostomy and 6 months after choledoco-enterostomy. Relief of jaundice was better after choledoco-enterostomy.
In the group of patients with associated biliary by pass and gastro-enterostomy, morbidity and mortality rates were 38% and 21.2%. Median survival time was 4.2 months. Delayed gastric emptying developed in 18% of patients who had gastroenterostomy at first laparotomy.
Biliary by-pass using common bile duct offers the best palliation for jaundiced patients. Gastroenterostomy should be performed on a selective basis only.

**BT093** ROLE OF RESECTIONAL SURGERY FOR PANCREATIC CANCER

C. Sperti, C. Pasquali, S. Catalini
C. Militello, V. Costantino,
F. Cappellazzo, S. Pedrazzoli,
Clinica Chirurgica 1-Padua, Italy

The management and prognosis of pancreatic duct carcinoma remains controversial. There is general agreement that radical resection offers the only chance of cure for patients with pancreatic cancer, and recently mortality and morbidity related to this procedure have been improved in many centers.
Aim of this study was to evaluate our experience of resectional procedures for pancreatic cancer and the progress in management of this tumor. Among 406 patients with pancreatic cancer observed in our Department from 1964 to 1988, 84 underwent resective surgery : 55 duodeno-pancreatectomy (DP), 13 total pancreatectomy (TP) and 16 left pancreatectomy (LP). 14 patients had vascular resection (7 DP, 6 TP, 1 LP) and 8 pylorus rreserving pancreatectomy (7 DP and 1 TP). In the last six years, 17 patients underwent subtotal duodeno-pancreatectomy (StDP) as the procedure of choice. Histologic examination showed 83 ductal carcinomas and 3 acinar cell carcinomas. Node metastases were found in 34 patients. The tumor was well differentiated in 50 cases, moderately in 24, poorly in 10. The resection was radical in 72 patients. Overall hospital mortality and morbidity were 14.3% and 42.6%. In the last 8 years hospital mortality decreased to 3% and resectability rate increased from 16.4% to 34.4%, with no improvement in rate of localized disease. 5-years actuarial survival rate was 14.5% (13.8% after DP, 0% after TP). Survival was found to be related to lynph node status ($p < 0.001$), degree of differentiation ($p < 0.05$) and radicality of resection ($p < 0.05$). Out of 17 patients who had StDP, operative mortality was 0, and median survival time was 12 months with a good quality of life. After vascular resection median survival time was 8 months.
Actually pancreatic resection can be performed with a low acceptable mortality and morbidity rate. Altough long term survival is a rare event, radical surgery is the procedure of choice for the best palliation of symptoms in patients with pancreatic cancer.

**BT094** PANCREATIC CANCER IN PATIENTS UNDER 40 YEARS OF AGE.

C. Sperti, C. Pasquali, S. Catalini,
C. Militello, R. Behboo, V. Yeroomian
S. Pedrazzoli
Clinica Chirurgica 1
University of Padua, Italy

Roughly 80% of cases of pancreatic cancer occur in patients over 60 years of age and cases under 40 years of age are extremely rare. In this study we review our experience with pancreatic carcinoma in patients under 40 years of age to determine the natural history of ductal adenocarcinoma of the pancreas in young patients. From 1964 to 1988 in our Department were observed 406 patients with pancreatic cancer; 26 (6%) of them had less than 40 years of age (19 M and 6 F, averaging 35.2, range 26-39 years). Cystadenocarcinomas and islet cells tumors were excluded. The most frequent symptoms were: jaundice (14) and abdominal pain with weight loss (11). 7 patients had history of chronic pancreatits (3 with pseudocysts) and 3 underwent laparotomy before admission in our department. 12 patients underwent explorative laparotomy only, 7 biliary bypass and 6 resective surgery (5 duodenopancreatectomy and 1 total pancreatectomy). Histology showed a well differentiated tumor in 9 patients, moderately in 5, poorly differentiated in 10 and chronic pancreatitis in 2 (false negative). Linph node metastases were found in 6 cases, liver or peritoneal spread in 12. Operative morbidity and mortality was 23 % and 11.5 % respectively. Actuarial survival rates were 11.5 % at 1 year and 0 % at 2 years, with a median survival of only 3 months.
Ductal adenocarcinoma of the pancreas in young patients is rare but is more aggressive than in older patients with an extremely poor survival. Differential diagnosis with chronic pancreatitis is particularly difficult when the tumor is in early stage and 1/5 had an histology of chronic pancreatitis before tumor spread became evident.

Study supported by CNR grant. Project "Oncology" # 88-00804.44

BT095 THE PAPILLO-ODDITIS: CLASSIFICATION AND TREATMENT.

C.Pietrantoni,A.Addari,P.DiStefano,F. Panzera,P.Simone,F.Torrelli,G.Panzera

"Umberto I" Hospital,Department of surgery,Tagliacozzo, ITALY

DEFINITION: Phlogosis of the Vater's papilla,usually secondary to benign biliary and/or pancreatic pathologies, that causes its transitory or definitive stricture with consequent partial obstacle to the flow of biliary and pancreatic secretions. CLASSIFICATION: On the basis of the Barraya's anatomical sphincteral scheme(papillary sphincter,inferior and superior sphincter of choledoch and Wirsung's duct),we divide: 1)papillitis:the stricture involves only the papillary sphincter; 2)papillo-odditis:the stricture involves the papillary and the inferior choledochal sphincter; 3)total papillo-odditis: the stricture involves tha papillary and the inferior sphincter of choledoch and Wirsung's duct. PHYSIOPATHOLOGY:In the papillitis there is a mild choledochal and pancreatic reflux;in the papillo-odditis there is a mild pancreatic and a severe biliary hypertension;in the total papillo-odditis there is a severe pancreatic and biliary hypertension. ETIOLOGY: The papillo-odditis have been recognized an outcome of the following pathologies:cholecystectomy (61.6%);papillary divulsion(18.6%);endoscopic papillotomy(5.8%);surgical papillotomy without papilloplasty(4.6%); echinococcus cyst opened in the biliary duct(5.8%);duodenal ulcer(4.6%). CASE-REPORT: In twelve years(1977-1989) we performed 608 operations of hepatobiliary surgery:87 cases (14.3%) were stenosing papillo-odditis(30 papillitis;41 papillo-odditis;16 total papillo-odditis).TREATMENT: In the papillitis we performed a papillotomy and papilloplasty;in the papillo-odditis a measured papilloinfundibolotomy and papilloplasty(38) and a total papillotomy and papilloplasty(3);in the total papillo-odditis a Wirsung's duct sphincterotomy.

**BT096** CHEMICALLY INDUCED HEPATIC DAMAGE: LIGHT AND ELECTRONMICROSCOPICAL STUDY

J. Ptashekas
Institute of Hygiene, Vilnius,
Lithuania, USSR

Particular study revealed data of light microscopical and ultrastructural analysis of hepatic parenchyma after experimental exposure to environmentally significant agricultural chemicals N-methylopirolidon and gamma-butyrolacton.

Wistar rats 220-280 gr b.w. were tube fed with a solution of the chemicals mentioned above with a dose of 1/20 LD50 for the total period of 30 days. Samples of hepatic parenchyma were taken immediately after termination and embedded in paraffin and epoxy rasins for light transmission electron microscopy followed by morphometry. Serial 5 mkm thickness paraffin sections were led-hematoxylin stained and then prepared for ultrastructural investigation to complete the morphological analysis of hepatic tissues.

Light microscopical investigation showed no difference in structure of hepatic parenchyma of control and exposed animals. Electron microscopical study in both experimental groups revealed destruction of hepatocytes microvilli, increased vacuolization level of their cytoplasma ($p<0.002$), reduced Disse space ($p<0.005$). Pretty remarkable changes were found in ultrastructure of erythrocytes within S-capillaries of hepatic parenchyma, N-methylopirolidon as well as gamma-butyrolacton circumstained appearance of complete wash out of the red blood cells matrix. Multiform skeleton of erythrocytes originated as the result of the process. In this case ultrastructural investigation appeared to be indispensable to state the blood circulation syndrome diagnosis in case of chemically induced hepatic damage.

### BT097 PANCREATIC ABSCESS (PA) AND PANCREATIC NECROSIS (PN)

Z.Puchalski,Z.Piotrowski,L.Trochimowicz,J.Snarska

Department of General Surgery,Medical Academy, Białystok, Poland

PA as a complication in acute pancreatitis occurs with a frequency of 1-10% cases. PA has become the most common cause of death in acute pancreatitis (AP). PN is defined as a diffuse bacterial inflamation of necrotic pancreatic and peripancreatic tissue without any significant bus collections. This study relating to our experience over the period 15 years is intended to shed light upon the problem of diagnosis and of the timing of surgical therapy for 33 patients with PA and 40 with PN. There were no siqnificant differences with regard to the cause of AP clear tendency with PA formation in patients from alcohol abuse. PA were located most frequently in the head and cauda of the pancreas. The diagnosis of PA and PN was based mainly of on clinical symptoms and on results of biochemical,radiological, USG and CT findings. The operative management we performed consisted in PA of wide incision of abscess and aspiration and drenage,in PN all necrotic pancreatic and peripancreatic tissues were removed.In PA group died 7 patients with repeated haemorrhage from abscess cavity, endotoxic shock and other general systemic changes. In PN group died also 7 patients with pulmonary and renal insufficiences and endotoxic shock.

BT098

### SURGICAL TREATMENT OF HILAR CHOLANGIOCARCINOMA

K. Radebold, J. Lange, J.R. Siewert
Dept. of Surgery, Techn. University Munich, W. Germany

Introduction: Even today treatment of hilar cholangiocarcinoma remains controversly:the evaluation of resectability varies from center to center;the results of an aggressive surgical approach with liver resection did not lead to establish an overall accepted standarised procedure.Treatment by drainage is discussed alternatively because of its lower morbitity and acceptable quality of life by a two-year survival of nearly one third of the patients.

Patients: Between July 1st 1982 and January 1oth 1990 24 patients with high bile duct cancer were treated operatively at the Dept. of Surgery,Technical University Munich. After exploration twelve patients (50%) underwent potentially curative resection, in 5 cases an additional liver resection became necessary to obtain tumor free margins. Reconstruction of the biliary enteric continuity was achieved by a hepaticojejunostomy Roux-en-Y. Eight anastomoses were splinted with tubes. In ten cases the tumor was unresectable because of local tumor spread, twice a palliative resection was performed.

Results: The operative mortality was 14% at 30 days. The median survival time of all 24 patients was 9,3 month (1.5-11month). Nine of the 12 patients with potentially curative resection died because of local tumor recurrence,three after recurrent septic complications.

Conclusion:Curative surgery in hilar cholangiocarcinama seems to be doubtful even in small tumors because of potentially associated tumor growing along perineural lymphatics into the third dimension at a distance from the primary; resective surgery with histologically clear margins to the proximal intrahepatic bileducts should be considered as palliative.

### BT099 PYLORUS PRESERVING OPERATION and the CARCINOMA of the AMPULLA of VATER

J. Ramos Dias, R. Moisão

Faculdade de Ciências Médicas, Lisboa

PORTUGAL

Four patients with carcinoma of the ampulla fo Vater where treated with pyloric preserving pancreatoduodenectomy without mortality or morbidity.

The AA present the fisiopatological, oncological and tecnical bases of this operation and study it's impact on the patients nutritional state.

The study was based on isotopic gastric evacuation, enterogastric reflux and GI transit examination, complemented by the determination of fecal fat and patients weight evolution.

The favorable nutricional evolution alied to the absence of complications related to the tecnique apoint the Traverso/Longmire operation as a suitable surgical procedure, with similar survival results as WHIPPLE classic operation, to deal with carcinoma of the ampulla of Vater.

### BT100 PERCUTANEOUS TRANSHEPATIC TREATMENT OF THE UNOPERABLE STENOSING NEOPLASMS OF THE BILIARY TREE

M.RAULE,R.ALEMANNO,M.MARINONI,R.ROSSI
SURGICAL PATHOLOGY ISTITUTE,MILAN,ITALY

The primitive or secondary stenosing neoplasms of the biliary tree are often observed in an advanced state,for the shortage and the a-specificity of the simptoms in the early stage.In this case the possibility of a radical operation is rather uncommon and has led to the development both surgical and alternative palliative techniques. The endoscopic and percutaneous transhepatic procedures offer a new approach to this pathology particularly in those cases where a surgical palliative diversion is not possible.This patients are old,jaundiced,at high risk and with a neoplastic diffusion that don't allow the surgical treatment. The placement of a pre-stenotic or trans-stenotic percutaneous transhepatic biliary drainage (P.T.B.D.) and the introduction of an endoprosthesis can represent the unique therapeùtical possibility.
35 unoperable cases of stenosing neoplasms of the biliary tree undergoing percutaneous procedure,selected from the series of Surgical Pathology Istitute of the Milan State University are presented.
The unoperability was assessed in 27 cases conservatively. The remaining cases underwent explorative laparotomy after P.T.B.D.
The Authors consider the results in terms of 30 and 60 days mortality,complications,and particularly quality of life,evalueted by the degree of self-sufficience. They conclude that the percutaneous techniques allow an early diagnosis both morphological and by exfoliative cytology from P.T.B.D. of malignancy, and that they are the unique extreme therapeutical procedure in those cases where any kind of operation is contrindicated.These procedures allow a reduction in the number of exfoliative laparotomy,that in these cases are often useless and harmful. In according to the type and number risk factors and to the tumor characterizations can preoperatorly be identified different classes of patients.This allow to plan the therapeutical strategy and to establish the prognosis more precisely.

### BT101 RESECTION OF A LARGE PANCREATODUODENAL TUMOR

J.G. Rothschild, M.D., R.B. Reinhold, M.D.
New England Medical Center, Boston, MA

We present the course of a 75 yr. old white male with biliary obstruction, cholangitis and gastric outlet obstruction.

One and a half years prior to presentation the patient had undergone gastrojejunostomy and choledochojejunostomy for a mass in the head of the pancreas. He was nine years status post cholecystectomy and common duct exploration with sphincteroplasty. Two months prior to admission he developed recurrent cholangitis and gastric outlet obstruction. CT revealed a large heterogeneous mass in the head of the pancreas, obstructing his bypass loop. Transhepatic drainage and TPN were initiated and he was transferred to our facility for consideration for resection.

This film presents the evaluation, operative course and pathology of this patient, highlighting the view that surgery, even if extensive, can often provide excellent palliation and occasional cures for extensive pancreato-duodenal malignancies.

**BT102** ENDOMETRIAL CYST OF THE LIVER

V.Rovati,E.Faleschini,G.Tagliabue,
F.Colturani,A.Benevento.
Cattedra di Patologia Chirurgica
Università di Milano

The presence of endometrial ectopic tissue in the liver is quite rare; only two cases are described in the international literature, and the Authors are now reporting another case they observed.(1) In a 37-year old female patient complaining of epigastric pain, the C.T. and U.S. showed a neoformation at the level of the 2nd and 3rd liver subsegment, suspected of being an echinococchus cyst owing to its peculiarities. The surgical removal of this cyst, which turned out to be adherent to the diapghragm, involved a left lateral segmentectomy. The histological examination of the surgical specimen denied the pre-surgery diagnosis and highlighted the presence of endometrial tissue in the mentioned neoformation. The patient then underwent gynaecological investigation and a pelvic ultrasonography highlighted the presence of a 4.8-cm cystic formation in the left ovary. A laparotomy and left ovariectomy were therefore performed on the patient, and the histological examination of the surgical specimen once again highlighted the presence of ectopic endometrial tissue. The etiological hypothesis of a tubal reflux of menstrual blood and blockage of endometrial cell elements between the diaphragm-peritoneal and the hepatic glissonian capsula is most likely for the liver location of endometriosis.(2,3)

References:

1) Graab A. et al.: J.Clin.Ultrasound 14:478, 1986
2) Steele R.W. et al.: Am.J.Reprod. Immunol. 6:33,1984
3) Jenkins S. et al.: Ostet.Gynecol. 67:335, 1986

**BT103** HEPATIC FOCAL NODULAR HYPERPLASIA (F.N.H.): PERSONAL EXPERIENCE

V. Rovati, E. Faleschini, G. Nervetti,
F. Colturani, G. Tagliabue
Cattedra di Patologia Chirurgica
Univ. Milano

This benign liver disease is quite rare and very little is known about it. It has not yet been demonstrated whether it is an attempt of remedial response to a parenchymal damage following a vascular arterial anomaly or real neoplasty.(1,4) What we are reasonably sure about is that it does not tend to degenerate and only very seldom gives origin to haemorrages due to spontaneous break.(1,2,3) The investigation methods, such as U.S., C.T. and angiography are often inadequate for diagnostic purposes and biopsy by thin needle does not provide sufficient quantities of tissue to carry out a differential diagnosis with the low-malignancy adenoma or adenocarcinoma.(2) The F.N.H. therefore requires a laparotomic investigation, often of a diagnostic as well as therapeutic nature. Three cases of F.N.H. have been observed in our experience: in two of them a single mass almost entirely occupied the 2nd and 3rd liver subsegment, whereas one case showed multiple masses located at the 2nd, 3rd, 4th and 5th subsegment. A correct pre-surgery diagnosis was only possible in one case. Two left lateral segmentectomies were performed in the first two cases and left lobectomy as well as sub-segmentectomy of the 5th subsegment were carried out in the third. There were no postoperative complications and the hospital course was 11 days.

References

1) Adson M.A. et al.: Surg.Clin.North Am. Vol.14 n.1: 198-216,1983
2) Ameriks J.A. et al: Arch.Surg.110:548-557,1975
3) Walt A.J. et al: Surg.Clin.North Am. Vol.10 n.2: 488-505, 1979
4) Whelan T.V. et al.: Ann.Surg. 177:150-158,1973

### BT104 STENOSIS OF THE COLON DUE TO PANCREATITIS

Sağlam A, Bilge A, Sözüer E. Resident
Erciyes University, Medical School, Kayseri, Turkey

Stenosis of the colon is an uncommon complication of pancreatitis, first reported by Forlini in 1927. In a recent study 34 published cases have been rewieved and two were reported (Lazarou and Economopoulos 1984). Since then, only one patient had been reported ın English literature (Marcello and Zegal 1983). We are representing a new case.

The patient 45-year-old woman had been admitted with complaint of epigastric pain radiating to both flanks, and vomiting. She has been given conservative therapy for acute pankreatitis diagnosed on physical examinations and laboratory findings. An epigastric mass was found on the tenth day of her hospital admission. The mass was seen as pseudokidney on ultrasonografic examination. Barium enema disclosed stenosis of the lumen and luminal irregularity of the transvers colon. At laparotomy, three weeks after admission, the transverse colon and its wall were thickened and have a rubbery consistency, and serosal surface had multiple echimotic patches.

This complication mimics colonic cancer but colonic stenosis is more commonly reversible. It is postulated that ischemia and pericolic inflamation is the cause of strictures (Hunt and Mildenhall). It would be reasonable to recommend a conservative approach in the majority of cases (Lazarou and Economopoulos 1984). In our patient abdominal mass had dissapeared in two months and pathologic changes on barium enema was improved.

References:
Forlini E. G Clin Med 1927: 8: 609.
Lazarou N, Economopoulos N. Isr J Med Sci 1984: 20:1073.
Marcello H, Zegal I. Amer J Gastroenterol 1983: 78: 28.
Hunt DR, Mildenhall P: Am J Dig Dis 1975: 20: 941.

### BT105 DIAGNOSTIC CRITERIA OF POSTOPERATIVE PANCREATITIS

N. Satoh, A. Igarashi, M. Kaburagi,
M. Tezuka, H. Kogure, Y. Tajima
Department of Surgery, Dokkyo University
School of Medicine, Tochigi, Japan.

Diagnosis of postoperative pancreatitis, particularly after abdominal surgery, is generally very difficult. Although several criteria for this diagnosis have been proposed, they are still controversial.

This study was undertaken to investigate the reliability of our diagnostic criteria of postoperative pancreatitis. These criteria composed of the following four items; 1) serum amylase levels greater than 500 DyeU/dl(normal,<220) at least once within 2 weeks postoperatively, 2) amylase isoenzyme including greater than 60% of pancreatic (p-type) isoenzyme, 3) amylase levels in the intraperitoneal drainage greater than 5,000 DyeU/dl, 4) amylase isoenzyme in the intraperitoneal drainage including greater than 80% of p-type isoenzyme.

This prospective study was performed in 188 major surgery cases excluding cases of pancreatitis. There were no cases which satisfied all of these four items. Two items(no.1 and 2), however, were both satisfied in five cases. These five cases were analyzed. There were four males and one female. Their ages ranged from 25 to 73 years, with a mean of 55.4 years. Operative procedures included total gastrectomy in two cases, cholecystectomy, partial resection of jejunum and Hartmann's operation in one respectively. Average serum amylase level was 763±332 DyeU/dl and p-type isoamylase was 91.3±5.5 %. The levels of such other serum pancreatic enzyms as lipase, trypsin and elastase 1 were elevated in all cases. As for clinical symptoms, high fever was noticed in three cases and abdominal pain in two. Diagnosis of postoperative pancreatitis was established in these five cases, in which two of the four items were satisfied.

It might be concluded that our criteria are useful for the diagnosis of postoperative pancreatitis.

### BT106 SIGNIFICANCE OF HEPATIC RESECTION FOR NON-COLORECTAL METASTASES

J. Scheele, A. Altendorf-Hofmann, R. Stangl
Department of Surgery, University Hospital, Erlangen, FRG

Hepatic resection has yielded a 20% to 40% five-year survival in colorectal metastases and is considered an accepted treatment for this condition. Surgical approach to "non-colorectal" secondaries (NCR), however, is still controversial, particularly if they derive from non-endocrine tumours. Up to now, only sporadic reports and few collected series are available (Hughes and Sugarbaker 1987).

This study includes 69 patients who underwent hepatic resection for metastatic spread from a variety of NCR-cancers between 1970 and 1988. Nine patients had endocrine tumours (carcinoid 7, gastrinoma 1, pheochromocytoma 1), and 60 had non-endocrine primaries with gastrointestinal malignancies playing a paramount role. There were 28 common hepatic resections, 15 segment-orientated resections, and 26 non-anatomical procedures. Surgery was classified potentially curative in 43 cases, whereas 11 patients demonstrated positive margins, and 15 patients had gross tumour left in place.

Among the endocrine tumours, six presented with significant symptoms that were relieved in five. Eight patients are presently alive, and one died 166 months after surgery without recurrence.

Within the non-endocrine sample median survival was 20 months following curative resection, and 9 months after palliative procedures. In the first group two of 11 patients operated prior to 1985 survived five years (cumulative survival 31 %), whereas no palliatively treated patient lived longer than 26 months. At present 17 patients are alive out of which eight are free of disease at 14 to 79 months.

These results are to preliminary to either recommend or refuse surgery in non-endocrine NCR-metastases. However, as prognostic benefit in most of the various malignancies involved is only marginal, this treatment should not be performed outside clinical trials.

References:
Hughes KS, Sugarbaker PH: in Rosenberg, Lippincott 1987
Cobourn CS, Makowa L, Langer B: 1987 Surg Gynecol Obstet 1987; 165: 239-246
Iwatsuki S, Starzl TE: Ann Surg 1988; 208: 421-434

### BT107 SPLENECTOMY FOR NOT SO FREQUENT INDICATIONS

J.M.SCHIAPPA, J.A.GONÇALVES*, J.GIRÃO

Hosp.dos Capuchos - Serviço 6 - Lisbon

H.Dist.Barreiro - Serviço de Cirurgia

PORTUGAL

Splenectomy is usually performed because of trauma, haematological diseases or during certain surgical elective procedures, as a complement to the surgical treatment.

The authors have, in their series of splenectomies, 15 cases which do not fall in the above mentioned indications and which may be considered "not so frequent" if not rare.

Certain of these 15 cases may be of controversial surgical indication, but their relative rarity makes it dificult to follow standards.

We present and discuss these 15 cases which are:

- Spleen ptosis = 2
- Local disease = 9 (Infections - Tuberculosis = 2
  Cysts - Pseudocysts = 1 / Parasitic Cysts= 2
  Aneurisms of the splenic artery = 4 )
- Controversial = 3 (Giant splenomegaly - Malaria = 1
  Kala-azar = 1
  Splenic infarction = 1)

BT108

### INFLAMMATORY RESPONSE IN THE MONITORING OF SEPTIC COMPLICATIONS IN ACUTE PANCREATITIS

U. Schöffel, M. Lausen, V. Gross,*

J. Schölmerich,* E.H. Farthmann

Departments of Surgery and *Medicine,

University of Freiburg, FRG

The degree of any inflammatory response can be evaluated by determining the activation of cellular and plasmatic systems.

In previous studies with inflammatory parameters in peritonitis, three substances turned out to reflect the severity of the inflammation with a remarkably high accuracy at an early point of the clinical course: Fibrinopeptide A as a marker of the activation of the factor XII dependent systems, the C3 split product C3a as a sign of the activation of the complement cascade, and the elastase-ã$_1$ proteinase inhibitor-complex as a marker of the activation of leukocytes.

Cut-off levels were calculated for these three parameters which predicted postoperative septic complications with a high accuracy so that an activation score from 0 to 3 was determined.

In a series of 50 consecutive patients with acute pancreatitis the use of that activation score was evaluated prospectively in the monitoring of septic complications. In the group which developed clinical sepsis (n=13) 10 patients died and 5 needed more than one operation. A score of 3 was observed in 5 patients on day 1, in 9 patients on day 3 and a score of 2 in 10 and 13 patients, respectively.

In the group with severe pancreatitis without sepsis (n=15) no patient died. A score of 3 was reached in 2 patients on day 1 and in 5 patients on day 3, with a score of 2 in 8 and 10 cases. In the group with less severe pancreatitis a score of 3 was never detectable.

Thus the occurrence of clinical sepsis could be predicted with a sensitivity of 69 % and a specificity of 86 % on the third day after admission. Grading the patients according to more than three Ranson's signs, a sensitivity of 92 % and a specificity of 59 % were found in this series.

**BT109** Pancreatic duct disruption

D.SEMBA, Y.WADA, Y.MORIOKA
First Surgical Department, Tokyo Univ
Tokyo, JAPAN

It is believed that the occurrence of pancreatic duct disruption precedes the clinical manifestation of massive pancreatic pleural effusion, and disruption is proved by Endoscopic Retrograde Pancreatography. Until now, it is not explained how duct disruption occur.

Materials and Method;

Pancreas specimens resected from 3 cases of massive pancreatic pleural effusion were investigated histopathologically.

Results;

1. In all specimens, there found no evidence of acute or chronic pancreatitis.
2. We can find only a single dilated duct with fibrosis , and this duct extended to the disruption site of the pancreas.
3. In this dilated duct, protein plugs were observed.

Comment;

It is suggested that the sequence of duct disruption may be induced with focal acute inflammation on a single branch of the duct system and protein plugs, which were elicited by alcohol ingestion.

BT110 TEN-YEARS EXPERIENCE OF PANCREATIC CYST SURGERY

A N Severtsev, E I Brekhov, I Y Kuleshov,
O M Tchekmarev
Surgical Clinic, Hospital No 51
Moscow, USSR

Despite a great number of researches in this field, surgical treatment of pancreatic cysts (PC) is still a problem. At present there is a great number of methods of surgical treatment of PCs.

From 1978 to 1988 the staff of our Clinic performed 55 pancreatic operations. There were 36 internal drainage PC operations and 12 external ones. During this period there were also 7 resections of different parts of the pancreas with the same pathology. 49 patients were operated on for chronic or acute pseudocysts, 6 patients for true pancreatic cysts. In 30 cases of drainage operations (both in external and internal drainage) the surgery was performed with already formed cyst wall which became clear from ultrasound investigation and computer scanning. Among patients with internal drainage in 18 cases laser CO2 was used at the stage of cyst wall dissection. The rest (18 patients) underwent conventional surgery (scalpel). These groups of patients were assessed according to the level of haemostasis, the number of ligatures, the duration of surgery and the results of cyst cavity reduction in the post operative period.

There were no post operative deaths. 11 patients developed severe post operative pancreatitis, which was cured therapeutically (there were patients who underwent acute pseudocyst pancreatic surgery without formed cyst wall and patients who underwent pancreato-duodenal resection). External pancreatic fistula develeped in 11 patients (all patients with external cyst drainage).

The use of CO2 laser shows the great advantage of this surgical 'instrument' in comparison with the conventional scalpel in all analysed parameters during pancreatic cyst operations.

BT111

### THE DELAYED APPROACH TO SURGERY OF GALLSTONE PANCREATITIS

M. Shalev, Z. Kaufman, B. Shpitz, A. Dinbar
Department of Surgery 'B', Meir Hospital,
Kfar Saba, Israel

A prospective study was undertaken to determine the safety of the delayed approach to surgery of gallstone pancreatitis. Thirty-four patients with gallstone pancreatitis were operated according to the delayed approach (2-7 days post pancreatitis) between the years 1984-88. Two patients had four positive signs according to Ranson's criteria; the remainder, three or fewer positive signs. The mean delay of operation after the acute phase was 8 days (range 2-13). This group was compared to 112 patients who, in the same period, underwent elective cholescystectomy due to gall-stones without clinical history of acute pancreatitis. The two groups were compared with respect to the mean postoperative hospitalization days, postoperative complications and mortality. No statistically significant difference was found between the two groups. Based on these data, it seems that the delayed approach to surgery for acute gallstone pancreatitis is as safe as an elective cholecystectomy and can therefore be recommended as a routine procedure for gallstone pancreatitis.

### BT112 ULTRASOUND DIAGNOSIS OF BILIARY DUCT CANCER

V. Shanturov, E. Chijova

Institute of Surgery, Irkutsk, USSR

16 patients with bile duct carcinoma were investigated by ultrasound in order to obtain a liver and biliary system assesment. There ages ranged from 36 to 84 years. The male to female rates was 10:6.

The sonographic findigs were: intraluminal hyperechogenic mass without acoustic shadow, bile ducts amputation, mass involving the biliary ducts with disturbance of inner contour, biliary tree dilataition, both narrow of distal and wide of proximal bile ducts and increasing the echogenicity of the liver parenchima.

Ultrasonography had considerable precision to finded the level of the obstraction in the biliary tract and to identify signs of spreadness of desease (distant metastasis, ascites).

Thereby ultrasonography is a helpful procedure to establish the preoperative diagnosis of bile duct carcinoma.

This simple and non invasive method may locate the site of biliary obstruction, outline the tumor itself and also determine the staging of the cancer.

**BT113** ELECTROHYDROLIC SHOCK WAVE LITHOTORIPSY BY PERCUTANEOUS TRANSHEPATIC CHOLANGIOFIBERSCOPY TO THE PATIENTS WITH INTRAHEPATIC GALL STONE

T. Shimizu, S. Hasegawa, Y. Tsuchiya
Shinrakuen Hospital, Niigata, JAPAN

Intrahepatic gall stones are more frequently encountered in Japan and Korea in Western countries. Occasionally, it is difficult for us to diagnose and treat the disease, and residual or recurrent stones can often occur after operation. 22 patients with intrahepatic gall stone were treated in our hospital. 2 patients were treated by papilloplasty or hepaticojejunostomy ( 1 patient suffered cholangitis frequently and 1 patient developed to secondary biliary cirrhosis ). 3 patients were operated upon hepatectomy and hepaticojejunostomy ( 2 patients occurred frequent cholangitis at postoperative period ). 5 patients were treated by hepatectomy ( 2 patients were diagnosed by remained stones and 1 patient was cached recurrence ). 12 patients were treated by the percutaneous transhepatic cholangioscopy and lithotoripsy by shock wave. Next stage of several months later, 3 patients with intrahepatic cystic dilatation remained were achieved hepatectomy and another 7 patients cured completely without operation. This two step therapy was no complications and no symptom and cholangitis of post-therapy. Now, this video illustrates the data and the techniques of percutaneous transhepatic cholangio-drainage, cholangioscopic lithotoripsy and cine-cholangiography of pre and post-therapy.

### BT114 A CLINICAL AND PATHOLOGIC ANALYSIS OF 61 RESECTED HEPATOMAS

T. Shimoyama, T. Shimizu, T. Nakagoe,
T. Hirano, T. Miura, M. Tomita
1st Department of Surgery
Nagasaki University School of Medicine
Nagasaki, Japan

The aim of the present study was to evaluate the factors affecting the patient's survival of hepatoma(HCC) associated with cirrhosis.

Clinical Materials: Sixty-one consecutive patients with HCC who underwent curative resection through 1970 were studied. Standard major hepatectomy (2 or 3 segments) was done for 15 cirrhotic patients and a limited resection than one segment for 46 cases.

Results: 1)Follow-up examination showed the major resection of cirrhotic liver promoted degeneration of the residual liver, and most of these died from hepatic falure rather than recurrence. There were significant differences in the mortality and survival rates between major hepatectomies and limited resection.
2)For small HCC(<2cm in size), ultrasound(US) had the highest detection rate(90%) compared with CT(66.7%) and angiography(55.6%).
3)Pathologically, encapsulation appears to be complete as tumor grew. Portal vein tumor thrombus(12.5%) and/or satellite tumor (31.2%) were frequent in those wigh larger than 2cm in size.
4)survival rate of the group with small HCC was far better than that of the groups with HCC larger than 2cm($p<0.05$).

Conclusion: Early detection of the tumor without portal vein thrombus and/or satellite tumor, and an adequate hepatic resection such as subsegmentectomy or segmentectomy are most important for the patient's survival.

### BT115 EXPERIMENTAL ACUTE PANCREATITIS IN DOGS

### Protective Effects of Zinc Sulphate and Aprotinin Combination

Sözüer E, Şen M, Sağlam A, Balkanlı S, Y Yeşilkaya. Resident

Erciyes University, Medical School Hospital, Kayseri, Turkey

Several therapeutic modalities, such as peritoneal lavage, cimetidine, fresh frozen plasma, prostoglandins, indomethacin, glukagon, somatostatin, beta-adrenergic agonist drugs, and aprotinin have been used in the treatment of acute pancreatitis. But the mortality rate from acute pancreatitis remains high.

Enzymatic autodigestion plays an important role in acute pancreatitis. Intrapancreatic activation of digestive enzymes such as trypsinogen can cause pancreatic damage. It was also suggested that lysosomes and lysosomal acid proteases may be involved in the pathogenesis of acute pancreatitis (Evander and Lindquist).

Zinc is an essential trace element for human and animals. Zinc has a stabilizing effect on lysosomal membranes and reduces the liberation of acid hydrolases (Chvapil and Ryan). Aprotinin is a low molecular weight protein and complexes with proteolytic enzymes and results in their inactivation.

We induced pancreatitis in Mongrel dogs by injecting otolog bile 1 ml/Kg + 20,000 U chemotyprisine into main pancreatic duct. The study was carried out pancreatitis induced 5 groups of dogs, including control and pre and postoperative Zinc Sulphate; only postoperative Zinc Sulphate; pre and postoperative Zinc Sulphate + Aprotinin; postoperative Zinc Sulphate + Aprotinin treatment groups.

We determined the degree of pancreatic damage by macroscopic and histopathological changes and biochemical investigations. In this study, therapeutic modalities after initiation of pancreatitis were ineffective. We suggest that pre and postoperative Zinc Sulphate + Aprotinin treatment gave the best results in this model.

### BT116 CHRONIC ADMINISTRATION OF URSODEOXYCHOLIC ACID DOES NOT IMPAIR THE GALLBLADDER MOVEMENT OR CHOLECYSTOKININ RELEASE IN HEALTHY SUBJECTS

S. Sumi, K. Inoue, S. Higashide, K. Takaori,

H. Minote, R. Hosotani, R. Doi, H. Kaji,

M. Yun, Y. J. Gu, K. Uchida* and T. Tobe.

First Dept. of Surgery, Faculty of Medicine and *College of Medical Technology,

Kyoto University, Sakyo-ku, Kyoto 606, Japan

Intraluminal cholic acids are reported to reduce CCK release and gallbladder (GB) contractility. This study exmined the influence of chronic administration of ursodeoxycholic acid (UDCA) on GB movement and cholecystokinin (CCK) release in healthy volunteers.
[METHODS] The following observations were performed before and after oral UDCA administation (600mg/day) for 2 weeks. GB movement was estimated by the percent change of the area on urtrasonic tomograph (pre-stimulation area as 100%). UDCA was not administered on the day of observations. 1. GB movement stimulated by exogenous CCK (caerulein 0.2 μg/kg, i.m.) was observed (n=5). 2. GB movement and CCK release stimulated by ingestion of chmically defined fatty diet (Clinimeal 89g, Eisai, Japan) were observed(n=5).
[RESULTS] 1. The duration of the contraction phase (time from the injection to the maximal contraction) was significantly ($p<0.05$) prolonged by UDCA administration (from 15±2 to 32±4 min), whereas the maximal contraction after the stimulation was not changed (from 35±7 to 31±4%). Serum total cholic acid concentration was significantly ($p<0.05$) increased (from 4.6±0.5 to 8.4±1.1 μmol/L). Fasting GB area was insignificantly increased (from 14.6±2.0 to 15.4±1.6 $cm^2$). 2. The maximal contraction after the ingestion was significantly ($P<0.05$) increased by UDCA administration (from 45±8 to 33± 7%), whereas it appered to be delayed (from 63±22 to 96±22 min). The integrated response of plasma CCK was significantly ($p<0.01$) greater after the UDCA administration (from 212±126 to 542±165 pmol-180 min/L) with a significantly ($p<0.05$) higher value at 90 min after the ingestion. Serum total cholic acid concentration was significantly ($p<0.05$) increased (from 4.3±0.4 to 9.6±1.4 μmol/L), whereas fasting GB area was insignificantly increased (from 12.6± 2.0 to 19.2±3.7 $cm^2$).
[CONCLUSION] Chronic administration of UDCA slowed but did not impair exogenously stimulated GB contraction. Endogenously stimulated CCK release and GB contraction were enhanced and appeared to be delayed by UDCA administration.

**BT117** EARLY SURGICAL TREATMENT OF ACUTE CHOLECYSTITIS: RESULTS OF A PROSPECTIVE TRIAL.

EM Targarona, F Roset, E Veloso, C Marco,
Serv. of Surgery. Hospital de Mutua de Terrassa. Terrassa. Barcelona. Spain.

The timing of surgical treatment in acute cholecystitis (AC) has been a controversial subjet for the last 20 years. The difficulty in stablishing the diagnosis preoperatively, technical difficulties and the high risk of most patients are all factors which have precluded the wide acceptance of early cholecystectomy.

AIM. The object of this study was to evaluate prospectively the applicability of an option of early surgery (Cholecystectomy within 7 days) in AC.

MATERIAL AND METHODS. Four hundred and twenty-five patients operated on for calculous biliary disease were studied prospectively. In all cases, ultrasonography (US) was performed. CA diagnoses were stablished in basis of clinical and US data. Of these, 119 experienced 122 episodes of AC (28%).

RESULTS. 8 patients were not operated due to the existence of severe risk factors, although three of these eigth (37%) suffered a new episode of AC treated by early surgery. Specificity of ultrasonography was 97% and sensivity, 93%. Of the 114 patients candidates for surgical treatment, 103 (90%) were operated on within 72 hours and 4 within 7 days. On the whole, 94% of patients were operated during the first week. No significant differences were found compared with 306 patients who underwent elective surgery for biliary lithiasis with respect to morbidity (22% vs 19%), mortality (3,4% vs 1,3%) or mean postoperative stay (12±8 days vs 13±9 days).

CONCLUSION. Surgery during the first admission is a safe and efficient treatment for AC, with morbidity and mortality not significantly higher than those observed in elective biliary surgery. Early surgery is associated with a shorter hospital stay, and avoids readmission for recurrence of the AC or late emergency surgery for poor evolution of patients under medical treatment. Thus, we recommend early cholecystectomy as the first therapeutic option in AC.

**BT118** CHOLECYSTECTOMY FOR DIFFERENT AGE GROUPS ESPECİALLY IN THE ELDERLY

TEVFİK Küçükpınar*,RECEP Çetin*,KEMAL Yandakçı*,GÜNDÜZ Tunç*,SETTAR Bostanoğlu*

(*) SSK Ankara Hospital I.Surgery Clinic Ankara,Turkey.

From the beginning of this century,the average human life has gained approximately 25 more years by means of the improvements in medical technology.So,it is guessed that in about 40-50 years in future,the elderly group will be more greater and will arrive nearly 50 milions in general population.In relation the number of operation which will be applied to that group will surely increase.The purpose of this study is to make a statement about the mortality and morbidity rates,in patients whom undergone cholecystectomy,over sixties,and to investigate what effects age has for cholecystectomies.

This study is put out in SSK Ankara Hospital,I.surgery clinic from 1985 to 1989 and involves 511 patients whom undergone cholecystectomy.389 were women(76.13%) and 122 men (23.87%).The mean age was 49(from 16 to 99).All of the diagnosis stand upon the histopathological reports taken after the surgery.

RESULTS : The patients were examined in 3 groups:

| | Postop. mortality | Morbidity | Postop. early complication |
|---|---|---|---|
| 1)Over sixties: (119 cases) | 5 (0.97 %) | 28(5.47%) | 10(1.95%) |
| 2)Between 50-60: (143 cases) | 3 (0.58 %) | 18(3.52%) | 8(1.56%) |
| 3)Under fifties: (249 cases) | 1 (0.19 %) | 10(1.95%) | 3(0.58%) |
| TOTAL (511 cases): | 9 (1.75 %) | 56(10.94%) | 21(4.10%) |

The numerical differances between the groups are found to be significantely meaningfull.(Student's "t" test)

COMMENT : It is seen that,the older a patient is at the time of cholecystectomy,the more likely it is for that patient to prevent with anacute biliery complication.Early cholecystectomy decreases the morbidity and mortality rates related with the naturel progression of cholelithiasis.Although non-operative menagement for asemptomatic or mildly semptomatic cholelithiasis has taken increasing attention,we can say that,early cholecystectomy or even a prophylactic application would prevent the morbiditiy,mortality andpostoperative complications of the gall stone disease.

### BT119 OUR ATTITUDE IN THE TREATMENT OF ACUTE PANCREATITIS

L Torde, V Arsenić, S Ratkov, M Popov

R Popov, V Palanački

Branka Radicevica 22, Zrenjanin

Yugoslavia

Vojvodina, a part of Yugoslavia in which our city is located, is known as a district with a lot of cases of acute pancreatitis; the incidence is 30 cases in 100,000 inhabitants. In the period 1983-1988 we treated 356 patients with the diagnosis of acute pancreatitits. Ranged by people there were 174 (48.9%) men and 182 (51.1%) women. The youngest patient was 21 and the eldest 83 years. 274 (82.6%) were treated conservatively and 62 (17.4%) had surgical treatment. According to the etiology, 240 patients (67.4%) had biliary calculosis, 72 (20.2%) patients had chronic alcoholism in anamnesis and 22 (12.4%) patients were in the group with unknown etiology and rare causes.

Diagnoses

- anamnesis and clinical visitation
- classic RTG diagnosis
- laboratory
- routine US
- CT was sporadically used

Patients were grouped using Hollenders' classification modified by Stulhofer in

| | |
|---|---|
| A - serous group | 283 patients (79.5%) |
| B - with focal necrosis | 30 patients ( 8.4%) |
| C - with global necrosis | 43 patients (12.1%) |

Our attitude in the treatment

- in the beginning nonoperative treatment
- surgery between the first and the third week
- in the case of biliary pancreatitis we perform cholecystectomy and insertion of T-drain
- after the third week complications are treated operatively

Survival and mortality

| | | |
|---|---|---|
| Total | survived | 334 patients (93.8%) |
| | died | 22 patients ( 6.2%) |
| In Group B and C | survived | 40 patients (64.5%) |
| | died | 22 patients (35.5%) |

Literature can be obtained by the author.

BT120 SURGERY FOR GALLSTONE PANCREATITIS

A Tsironis, D Papadimitriou
A Kikiardopoulou, G Gennadios
Municipal Hospital, Athens, Greece

Acute pancreatitis is associated in most cases in western Europe with the presence of gallstones (Durr 1979). It is also well established that elimination of biliary disease reduces the recurrence from 75% to less than 10% (Frey 1981). Ranson's signs determine the severity and disease mortality (Heiz 1985). Some authors prefer early surgery (Acosta 1978, Heiz 1985) to prevent pancreatic necrosis, whereas others advocate initial conservative treatment followed by elective surgery in the same hospitalization (Kelly 1988).

Between 1978 and 1989 we accepted 58 patients with acute gallstone pancreatitis who underwent emergency (31%) or briefly delayed operations (69%) during the same hospitalization. 17.5% had more than three Ranson's signs. Patients who had an early operation and more than three Ranson's signs had a significant increase in morbidity and mortality.

We concluded that briefly delayed operations during the same hospitalization can be performed safely after resolution of acute pancreatitis.

References:
Durr G H K. The exocrine pancreas. Saunders 1979: 352
Frey C F. Surg Cl N Am 1981: 61: 923
Heiz et al. Am J Surg 1985: 149: 371
Acosta J M et al. Surgery 1978: 83: 367
Kelly T R. Surgery 1988: 104: 603

### BT121 *IS LIVER INNOCENT IN ACUTE RENAL FAILURE DUE TO HYPERBILIRUBINEMIA?*

***Mehmet TUĞCU.Rasih YILMAZ,Gül YÜCE,

Orhan ÖZBAL***

***General Surgery and Pathology Dept's
Medical School Ege U. Izmir/TURKEY***

*Acute renal failure is a serious complication of hyperbilirubinemic state.The liver is under hemodynamic and physiopathologic pressure of obstructive lesion in those patients.The paranchimal and functional derangement of liver accompanies to destructive effects of hyperbilirubinemia.*

*In this experimental study,CDCA (choledochocaval anastomosis) model is created in 14 Mongrei dogs as described formerly by Green at all and the liver is protected against the cholestatic effects of obstruction.*

*The microscopical findings : Centrlobular fatty degeneration and mimimal cholestasis in 11 (78 %).The typical findings of biliary obstruction(Connective tissue increase,edema,proliferation of biliary canalicules,plugs in main biliary tractus,hairy degeration) were not present in any dogs except one.*

*Liver function tests were in normal ranges in preoperative and postoperative period.*

*Although this "innocent" liver,the histologic and biochemical findings of "destructed" kidney is found in 8 of this series as present in connected research.*

*These results suggest that the liver is not so responsible for destructive findings in hyperbilirubinemic state.*

BT122 THE ROLE OF SPHINCTERETOMY IN PANCREATIC SURGERY.

G. VIOLA
Istituto di Chirurgia Sperimentale
Università di Napoli

In pancreatic disease, a direct surgical approach to the ampulla of Vater, remains questionable, though the close relations with the gland.

In gallstone-related acute pancreatitis the aim of a transduodenal sphincteroplasty is to decompress both,biliary and pancreatic ducts. In our experience, since 1985, among 21 consecutive patients,14(66,6%)underwent a biliary operation within the first week after the admission. Only in one case(4,76%) impacted stone was found and sphincteroplasty was performed. Two patients(9,52%) in whom free residual stones were present, received a bilioenteric anastomosis. In all of the others operated on(52,36%), only a cholecistectomy was performed, with the aim of preventing recurrent migration of gallstones.

In chronic alcoholic pancreatitis the drainage of pancreatic duct by section of the ampullary system, can be considered also in cases presenting with most severe parenchimal diseases. Among our 39 patients operated on for chronic alcoholic pancreatitis, only 7(17,9%) underwent a retrograde duct drainage for the rarety of distal segmental obstruction.

In none of our 18 cases of biliary obstruction due to enclosement of the bile duct in a fibrotic pancreas, stricture was located in the terminal tract of choledocus.

Conclusions: Transduodenal surgical approach to the ampulla may be useful in selected partients(10,2%,in direct experience) if submitted to correct indications,deduced from a complete pre- and per- operatory study.

BT123 ORGAN PRESERVING OPERATIONS IN OBSTRUCTIVE JAUNDICE SYNDROME

I.Viyachki,P.Tokov,N.Yaramov,

T.Pozharliev,D.Viyachki

Medical academy-Sofia

Personal experience with 1337 patients presenting acute and exacerbated diseases of the hepatobiliary and pancreatic system,of which 410 complicated by obstructive jaundice,admitted for treatment in the Emergency Surgery Department-Medical Academy,is shared.
Endoscopic retrograde cholangiopancreatography is used as a diagnostic therapeutic method in 545 patients and endoscopic papillosphincterotomy-in 312 patients. Only 112 cases are subjected to operation.
Experience with endoscopic debarrassement of the extrahepatic bile ducts,performed in the pre-and post-operative period is discussed.
The operations used comprise:cholecystectomy-59,choledochoduodenoanastomosis-16,common bile duct drainage according to Kerr-18,transcystic drain-5,cholecystostomy 2,explorative laparotomy-2,choledochojejunoanastomosis plus Braun-5,choledochojejunoanastomosis with gastroenteroanastomosis plus Braun-4,cholecystogastroanastomosis-1.
Analysis of the clinical case material,and comparative assessment of the results with data from the pertinent literature,lead to a number of clinically relevant conclusions.

### BT124 POSTOPERATIVE-POSTTRAUMATIC ACUTE ACALCULOUS CHOLECYSTITIS.

P.Vrachnos,L.Papastamatiou,A.Alonistiotis,L.Sideris,E.Kapositas.
Dept.of Surgery.Apostle Paul Hosp.-KAT
ATHENS-HELLAS.

Many factors have been implicated in the pathogenesis of "post stress" cholecystitis,which is a rare pathologic entity of unclear aetiology.Since the first description(Duncan 1844) until 15 years from today,only 258 cases were reported.Recent diagnostic imaging procedures helped to detect the disease and a worldwide spectrum of another 208 cases are reported.(Cornwell 1989)

As aetiology remains obscure,pronounced biliary stasis, shock,high viscosity of bile,disturbed hormonal regulation following parenteral nutrition,massive transfusions and endotoxemia,which activate pathways to gallbladder inflammation,regional hypoperfusion in the liver and gallbladder etc.suggested to be involved.

In a 4-year period,January1986-December 1989 eight out of 22 cases of acute cholecystitis were of acalculous form(AAC).Five cases developed among intensive care unit (ICU)patients and another three among multi-injured individuals.The clinical evaluation was rather difficult in all 8 patients(5 men-3 women).The time of onset of the disease varried from 8 to 23 days after trauma and/or operation.The ICU patients were still intubated and the other three under prolonged I.V. hyperalimentation.Diagnosis was in two patients delayed and in another two a more fulminant course of the disease caused an early perforation.Emergency cholecystectomy was in all cases achieved.Two patients died because of sepsis.

It has to be pointed out that AAC is a rare phenomenon with fulminant course and high mortality,because exact diagnosis is not easy.As pain is hardly detectable in multi-injured and especially in intubated patients,fever and jaundice may be of diverse origin,lab exams are of secondary value and only repeated ultrasonography and peritoneal lavage may confirm the first clinical thought. This last has to be based on evaluation of predisposing factors rather than to clinical signs.Immediate cholecystectomy is the procedure of choice.

### BT125 INTERDISCIPLINARY MANAGEMENT IN ACUTE NECROTIZING PANCREATITIS BY OPEN PACKING

H.W. Waclawiczek, O. Boeckl

Ist Surg. Dept. and Ludwig-Boltzmann-Inst.
Landeskrankenanstalten Salzburg, Austria

Acute necrotizing pancreatitis causing peritonitis and organic failures is still a central problem in gastrointestinal surgery. The mortality ranges from 30 to 70% in the literature. Therefore the treatment requires good teamwork between surgery and intensive care from the very beginning. Since 1984 34 patients (26 males, 8 females, average age 37a) were treated in our hospital according to following therapeutical concept:

Preoperative phase: Basically we always attempted to bring the patients into the "postacute phase" of the disease (12th day after onset of disease) with optimal intensive care therapy, because then secondary complications as necroses and abscesses have to be faced. This management was successful in 25 of the 34 cases; early operation was undertaken only if organic failures of the lung (ARDS) occurred (n = 9).

Operative phase: Bicostal laparotomy - dissection of the gastrocolic ligament - opening of the retrogastric space - removal of the necroses by digitoclastic necrosectomy - lavage of the pancreatic space with physiological saline (up to 10 l) - drainage of the peripancreatic space and subphrenic spaces - no closure of the laparotomy but open packing with 5 to 9 cloths soaked in 10% povidine iodine (PVP-I).

Postoperative phase: In the following 2 to 3 weeks the pancreatic space was explored through the laparostomy daily and necroses were removed. Furthermore a continuous lavage with 5 to 10 l physiological saline was performed. For this procedure long-term respiration (average 16 days) was necessary in all cases. The preoperatively started intensive care measures were continued till the abdominal wall was closed (about 25th postop. day).

Results: By this management the lethality of acute necrotizing pancreatitis with infected necroses could be decreased to 17.6% (6/34). Before 1984 the mortality amounted to 70%.

The advantages of the open packing method are the possibility of the daily exploration. By exact necrosectomy healthy pancreatic tissue can be preserved; only 2 patients became diabetic.

### BT126 DO WE UNDERDIAGNOSE PANCREATITIS IN CHILDREN?

R Wheeler, AS Najmaldin, CD Johnson
Southampton General Hospital, Southampton
UK.

Recurrent abdominal pain in late childhood and early adolescence is a common, but poorly understood problem. Pancreatitis in this age group is unusual, but may be under-diagnosed (1,2). The aim of this prospective study was to evaluate the diagnostic yield of routine serum amylase estimation in children with recurrent abdominal pain. In addition a retrospective search was made in our records for cases of acute pancreatitis.

Our paediatric surgical unit provides a general service for all cases arising in our district (population 417,000) and specialised services for a regional population of 2,000,000. All children requiring a second or subsequent admission for abdominal pain had the serum amylase measured as part of their routine investigation. Admissions to the paediatric medical wards were included in the survey. A four year review of the records of the surgical and medical paediatric units showed no cases of acute pancreatitis.

Thirty two children, median age 8 years (range 3-16) were admitted for a second or subsequent time on 37 occasions with abdominal pain, undiagnosed after the first admission. All had the serum amylase measured on admission, 14-56 hours (mean 34 hours) after the onset of pain: The mean amylase value was 112 IU/L (range 43 -204). A final pathological diagnosis was made in 5 children: urine infection(2), pelvi-ureteric junction obstruction (1), gallstones (2). The two gallstone patients (ages 13,16) underwent cholecystectomy with no evidence of pancreatitis in either case. Of the remainder, 11 were characterised as functional, periodic syndrome or constipation. Sixteen children are not yet diagnosed. No cases of pancreatitis were seen on the paediatric medical or surgical wards during this period, when 6314 patients were admitted.

We conclude that serum amylase is of little value in the routine investigation of recurrent abdominal pain in children, and that acute pancreatitis is extremely uncommon in this age group.

1. Standfield NJ, Howard ER. 2nd World Congress on Hepato-pancreato Biliary Surgery, Amsterdam 1988, FP 104.
2. Oldham KT et al, ibid FP 105

### BT127 OBSTRUCTIVE JAUNDICE - A STUDY OF 220 PATIENTS

JD Wig, NM Gupta, SM Bose,
RN Katariya, SK Khanna

Department of Surgery,
P.G.I.M.E.R., Chandigarh, India.

Surgery in patients with obstructive jaundice is still associated with a high postoperative morbidity and a significant mortality. The purpose of this presentation is to describe our experience of the problems encountered in the management of jaundiced patients.

The case records of 220 patients with obstructive jaundice who underwent surgery at our centre during a 12 year period were reviewed. A total of 134 patients (60.9%) had a malignant cause. The biliary tree was visualized by either percutaneous transhepatic or retrograde cholangiography. Ultrasound and CT scans were performed in the later part of the study. The obstruction was caused by common duct stones (n=68) carcinoma pancreas (n=63), extrahepatic bile duct carcinoma with secondaries in porta hepatis (n=31), gall bladder carcinoma (n=25), ampulla of Vater carcinoma (n=15), stricture CBD (n=5) and miscellaneous causes like pancreatitis and tuberculosis (n=6). The overall in-hospital mortality was 14%. Renal dysfunction occured in 12%, wound infection/dehiscence in 14% and gastrointestinal bleeding in 7%.

It is concluded that surgery for obstructive jaundice is still associated with significant morbidity and mortality. Late presentation, malignant nature of disease and very high level of bilirubin had an unfavourable effect on the results of surgical treatment.

**BT128** AGGRESSIVE SURGICAL PALLIATION OF PROXIMAL BILE DUCT CANCER WITH INVOLVEMENT OF BOTH MAIN HEPATIC DUCTS

D.K.Wilker,J.R.Izbicki,H.Mandelkow,L.Schweiberer

Dept. of Surgery,University of Munich,FRG

The only curative treatment for proximal bile duct cancer with involvement of both main hepatic ducts is liver transplantation. Most patients do not fulfill the requirements for liver transplantation.Our treatment strategy in adequate cases is palliative tumor resection and reconstruction of bile passage by sutureless bilioenteric anastomosis.In 12 patients this therapeutic regimen was followed,in 5 patients it was combined with intraluminal and percutaneous radiotherapy.One patient died of cardiac failure,no patient showed life-threatening complications,although the majority of patients exhibited serious operative risk-factors.Our treatment led to an effective decompression of the biliary system in 7 patients.2 patients exhibited a delayed decrease of serum bilirubin and two patients did not have any benefit from this procedure.Analysis of tumor characteristics showed that this aggressive approach leads to effective palliation in selected cases,where the tumor mass can be reduced by surgical means to microscopic residual tumor.Lymph node metastases or involvement of other organs do not influence the effect of operation.Survival times of our series compare favourably to survival after liver transplantation.Moreover our approach offers a reasonable quality of life after operation without long term drains.

**BT129** ACUTE CHOLECYSTITIS AND ASSOCIATED BILIARY PROBLEMS.

E.Xavier da Cunha, A. Milheiro, J.A.R.Martins A.Gouveia e Melo and F.Castro Sousa.

Coimbra University Hospital, 3th Surgical Department, PORTUGAL.

Acute cholecystitis, may be associated with some important biliary problems, which can influence surgical results.

During the last 14 years, in our Department, 173 patients were operated on for acute cholecystitis and at least one biliary problem was present in 41 patients (23.7%).

These patients were mainly female (63.4%) had a mean age of 65.7 years and the presence of an associated biliary problem was suspected or diagnosed preoperatively in only 17.1% of the cases. Their mortality rate was 14.6% *versus* 2.3% observed in the remaining 132 patients with uncomplicated acute cholecystitis.

Common bile duct stones (CBD), the first major finding, was present in 25 patients (14.5%), perforation of the gallbladder in 20 patients (11.6) - six having concomitant CBD stones, and two patients had a carcinoma of gallbladder (1.2%).

Mortality of patients with acute cholecystitis and CBD stones was 12% which is six times higher than the mortality of patients with chronic cholecystitis and CBD stones (2%) and this difference is statisticaly significant ($p<0,05$). Mortality of 148 patients with acute cholecystitis without CBD stones was 4.1%.

Finaly mortality of perforated gallbladder was 10% while mortality for acute cholecystitis without gallbladder perforation (153 patients) was 4.6%.

We may conclude that the association of biliary problems, namely CBD stones or gallbladder perforation with acute cholecystitis is rather frequent. Prognosis is considerabily worsened by their presence.

### BT130 PREVENTION OF PANCREATIC FISTULA IN PANCREATICODUODENECTOMY

Xianwei Dai et al
Department of Surgery, The Third Clinical College, China Medical University
PR China

Pancreatic fistula is the most dangerous complication of pancreaticoduodenectomy. In recent 5 years, pancreaticoduodenectomies without pancreatic fistula had been performed by us for 25 cases of pancreatic cancers or carcinomas of Vater's ampulla.

Our technique was as follows: dissecting the pancreatic duct about 1 cm during transecting the pancreas; inserting a 10 cm long silicon tube with 3 or 4 side holes into the distal pancreatic duct, the diameter of the tube was similar to that of the dilated pancreatic duct; suturing the pancreatic duct orifice and ligating it to the silicon tube with a 1/0 silk; dividing the pancreatic stump about 2 cm between the pancreas and the splenic vein; making the end-to-end pancreaticoduodenostomy with two layer silk sutures by invaginating the pancreatic stump into the jejunal lumen (as a sleeve). About 8-10 cm distal to the pancreaticojejunostomy end-to-side choledochojejunostomy using a single layer suture was made, generally without T-tube drainage. About 35-40 cm distal to the second anastomosis end-to-side gastrojejunostomy was made. Sometimes a jejunostomy with a silicon catheter as 0.3 cm in diameter was made for preparing postoperative enteric nutrition. Two abdominal drains were placed under the first and the second anastomosis respectively for 8-9 days after operation.

By using the above procedure, there were no pancreatic fistula and biliary fistula in our series. The authors believe that this surgical technique could successfully prevent the dreaded complication of pancreatic fistula in pancreaticoduodenectomy by invaginating the pancreatic stump into the lumen of jejunum well.

### BT131 SURGERY FOR HYDATID DISEASE OF THE LIVER

E.Yılmaz, N.Gökok
Gazi University, Faculty of Medicine
Department of General Surgery,Ankara,Turkey

Operations for hydatid disease of the liver constituted almost 2 % of our operations..We present our experience with 44 out of 58 cases of hydatid disease of the liver which could be fully documented in the last 3 years. Two-thirds were female. Average age was 44. The most common symptom was abdominal pain (63.6 %) and the most common sign was abdominal mass (48 %) Eosinophilia, positive Weinberg and Cassoni tests, ultrasonography and CT scanning are the major tools for diagnosis. There were 67 cysts, 49 (73 %) located at the right, 18 (27 %) located at the left lobe. 12 (18 %) were complicated. The most common complication was intrabiliary rupture. After evacuation of the cyst, we managed the cyst cavity with one or more of the following procedures: omentoplasty, tube-drainage, capitonnage, partial cystectomy, cystectomy and scolicidal agent injection. Infected cases were drained, choledochotomy and internal or external drainage were performed for intrabilary ruptured cases. Tube drainage and omentoplasty did not increase mortality. The average hospital stay was 11 days. There was no operative mortality.

### BT132 DIAGNOSIS OF GALLBLADDER CARCINOMA

Keisuke Yoshida, Takeaki Shimizu, Kazuhiro Tsukada, Yoshio Shirai, Yoshiaki Tsuchiya, Katsuyuki Uchida and Terukazu Muto.
First Department of Surgery, Niigata University School of Medicine and Shinrakuen Hospital, JAPAN

For the purpose of improvement of the diagnostic rate of carcinoma of the gallbladder (GBC), the accuracy of clinical diagnosis of this disease was studied based on its macroscopic form. During the years 1982 to 1988, 251 GBCs were resected at our clinic and its facilitate institutions. These carcinomas were divided into two groups: early GBC (Nevin's stage I or II, 88 cases) and invasive GBC (Nevin's stage III or IV or V, 163 cases). Grossly, early carcinomas were protruded in 36 patients, superficial elevated in 27 patients and superficial flat in 25 patients. Out of 36 patients with protruded lesions, 29(81%) were diagnosed preoperatively by imaging technics. Seventeen patients (63%) with superficial elevated lesion were diagnosed pre or intraoperatively. Twenty four patients (96%) with superficial flat lesion were first diagnosed by pathologists after operation. Invasive carcinomas were papillary in 22 patients, nodular in 61 patients, flat infiltrating in 35 patients and early like in 45 patients. Most of patients with papillary or nodular or early like protruded lesion were well diagnosed before or during operation. Less than half of patients (42%) with flat infiltrating or early like superficial lesion were diagnosed accurately before pathological examination. The accurate detection of GBC is still difficult before operation because half of GBCs showed superficial or flat infiltrating lesions. The precise macroscopic observation of every gallbladder specimen removed should be performed.

BT133

A NEW THERAPEUTIC APPROACHES TO TREATMENT OF HIGH RISK PATIENTS IN BILIARY SURGERY

Pomelov V.S., Zhumadilov Zh.Sh.,Makarenkova R.V.,Taygulov E.A.

Vishnevsky institute of surgery, Moscow, U.S.S.R.

The treatment of acute complicated cholecystitis in patients with high risk of urgent operation is a difficult problem in biliary surgery. The prevalence of this diseases, high incidence of postoperative septic complications and high mortality rate require the further creation of more effective methods of treatment.

A new method of delivery of antibiotic to the biliary system by means of autological erythrocyte ghosts has been worked out experimantally and applied clinically in the patients with acute complicated cholecystitis and high risk of urgent operation. We infused intravenously of autological erythrocytes ghosts containing antibiotic only once every day. Clinical trial consisted of two groups of patients with acute complicated cholecystitis.
Group 1- 66 patients who received traditional conservative therapy and aminoglicosides by intravenously or intramuscularly route.
Group 2- 56 patients who received traditional conservative therapy and similar antibiotics by above stated special technique. No significant differences were noted among both groups as to sex, age and diagnosis. The results of conservative therapy of different methods are listed in table.

| Patients | No. of Patients | Age,yrs. | No.(%)of septic complications of disease | No.(%)of urgent operations | Disappearance of clinical, laboratory symptoms of disease |
|---|---|---|---|---|---|
| Group 1 | 66 | 56.8±0.2 | 29(43.9) | 19(28.8) | 9.0±0.04 days |
| Group 2 | 56 | 56.7±0.4 | 0 | 0 | 2.1±0.01 days |

It should be noted that, in patients of group 2, the amelioration of immunological and clinicals'state was more marked. The patients of group 2 after detailed examination and preoperative management of multiple system disordes were underwent an elective operation. The special pharmacokinetic investigations have shown a high and long concentration of antibiotic in the bile of patients (4-5 times) having new method of delivery of antibiotic to the biliary systems versus patients who received intravenously or intramuscularly infusion of antobiotic.

So, this new method is effective, available for clinical practice. It is also economical and can be used in biliary surgery especially in the high risk patients.

BT134

### CHANGES IN THE LEVEL OF GLUTATHIONE AND ACTIVITY OF GLUTATHION-LINKED ENZYMES IN PATIENTS WITH ACUTE COMPLICATED CHOLECYSTITIS

Zhumadilov Zh.Sh.,Korotkina R.N.,Pomelov V.S.,Karelin A.A.

Vishnevsky institute of surgery, Moscow, U.S.S.R.

Glutathione (GSH) and glutathion-linked enzymes are playing the most important role in detoxication processes and the cell protection against the oxidative stress. We have investigated the level of GSH and activity of glutathion-linked enzymes (glutathione reductase, glutathione peroxidase, glutathione-S-transferase) of erythrocytes in 30 patients with acute complicated cholecystitis and in 15 healthy subjects. Our results showed that the level of GSH and activity of glutathione reductase, glutathione peroxidase, glutathione-S-transferase in patients were reduced compared with healthy subjects (0.88±0.04 versus 1.6±0.04 nM/mg/min, 0.8+0.1 versus 1.3±0.1 nM/mg/min, 9.5+1.7 versus 11.6±1.4 nM/mg/min, 2.4±0.3 versus 4.9±0.4 nM/mg/min, respectively). Traditional conservative therapy did not resultes in increasing of the level of GSH in patients with acute complicated cholecystitis during a first week period their hospitalization in despit of amelioration of clinical patients'state.

The level of GSH was significantly (40-60 per cent) decreased in patients with peritonitis. The regressive changes of the level of glutathione and activity of glutathion-linked enzymes were more marked in patients with both preoperative and postoperative septic complication. It should be noted that the regressive changes take place long before the clinical symptoms'development of postoperative septic complications.

Temporary surgical intervention and intensive conservative therapy resulted in amelioration of patients'state. At this period the GSH level and glutathion-linked enzymes activity increased, but didn't reach the control level by the decharge from the hospital.

The obtained data showed the GSH and glutathion-linked enzymes'role in pathogenesis of surgical disease and required the further creation of special methods of their correction.

### BT135 OESOPHAGEAL VARICES IN BILIARY ATRESIA

M Davenport ER Howard

Dept. of Surgery, King's College Hospital
London

Since 1981 90 children with Extrahepatic Biliary Atresia (EHBA) have undergone flexible fibreoptic oesophagogastroduodenoscopy (OGD) in order to diagnose and treat oesophageal varices. Varices were assessed as absent or present (Grades I - III). This series was examined retrospectively to examine its natural history.

Group I (n=49) consisted of those children who had no or Grade I varices at initial examination (median age = 3.8 yrs) and served as controls.
Group II (n=41) had Grade II or III varices at initial examination.

**Results :**
The timing of the original portoenterostomy had no influence on variceal grade (median age at surgery was 70 days in both groups; range =36-147 days (GpI) and 35-210 days (GpII) : P= 0.66). One in Group I and 20 (50%) in Group II had an episode of bleeding prior to OGD. There was no difference in the incidence of cholangitis (6 (12%) in GpI and 3 (7%) in GpII). At initial endoscopy liver function indices (bilirubin, glutamyl transpeptidase and alkaline phosphatase) were all significantly higher in GpII ($P<0.05$). There was no variceal progression in Group I.

**Conclusion :**
The presence of varices in EHBA is a common finding indicative of continuing liver dysfunction. The development of varices is not predtermined by age at initial surgery and does not seem related to previous episodes of cholangitis

